# The 30^th^ Scientific Conference of the Society on NeuroImmune Pharmacology in Annapolis, Maryland, May 3^rd^ – 6^th^, 2026

**DOI:** 10.1515/nipt-2026-0006

**Published:** 2026-04-08

**Authors:** Linda Chang, Susmita Sil, Jerel Adam Fields, Allison Andrews, Servio Ramirez, Richard Noel

**Affiliations:** Department of Diagnostic Radiology and Neurology, Nuclear Medicine, and Neurology, 12264 University of Maryland School of Medicine, Baltimore, MD, USA; Department of Neurology, Johns Hopkins University School of Medicine, Baltimore, MD, USA; Department of Pharmacology and Experimental Neuroscience, University of Nebraska Medical Center, Omaha, NE, USA; Department of Psychiatry, University of California San Diego College of Medicine, Vc-health Sciences, La Jolla, CA, USA; Department of Pathology, Immunology, and Laboratory Medicine, College of Medicine, University of Florida, Gainesville, FL, USA; Basic Sciences Department, School of Medicine, Ponce Health Sciences University, Ponce, PR, USA

**Keywords:** neuro-HIV, substance use disorders, neurodegenerative disorders, neuroimmunology, pharmacology, therapeutics

## Abstract

The 30^th^ Annual Society on Neuroimmune Pharmacology (SNIP) conference will be held on May 3-6^th^ at the Graduate Hotel by Hilton in Annapolis, Maryland. This 4-day conference will present preclinical, translational, and clinical research in the intersecting fields of neuro-HIV and substance use disorders, as well as related neurodegenerative conditions. The speakers and poster presenters will share cutting-edge research funded by the National Institutes of Health. On the first day, we will have two concurrent preconference symposia. The first is “Single Cell HIV and SUD Effects on the Brain: SCORCH Consortium Progress”, with an overview and 7 presentations by investigators, highlighting the outstanding work in the Single Cell Opioid Response in the Context of HIV (SCORCH). The second concurrent symposium is “Catalyzing Interdisciplinary Research on HIV-Associated Co-occurring Conditions.” In the evening, we will have our first Poster Session with 52 abstracts by early-stage-career investigators (ECIs), including those that received the ECI travel awards. On days 2-4, in addition to a plenary talk and two memorial lectures, we will have 11 symposia, with 62 speakers (including 12 who are early-stage career investigator travel awardees), and 65 additional poster presentations. In total, the 30^th^ SNIP conference received 185 abstracts for the 70 oral presentations and 115 posters. Topics covered by these symposia and poster presentations include mechanistic and observational studies that evaluate neuronal injury and neuroinflammation associated with HIV brain infection, and how drugs of abuse, including stimulants, opioids, and cannabis, may exacerbate or mitigate neuropathogenesis. In addition, with the aging population of people with HIV and many with substance use disorders, recent work also evaluated how aging and various neurodegenerative disorders could further impact brain health. At the plenary lecture, Dr. Nora Volkow will highlight the priorities of HIV research at the National Institute on Drug Abuse (NIDA), while our banquet speaker, Dr. Avindra Nath will elucidate how viruses, particularly retroviruses, may invade the brain, infect brain cells, and adapt to the local environment for decades or mutate and possibly lead to neurodegenerative disorders. This will be an exciting conference that will continue SNIP’s emphasis on the career development of early-stage investigators.

## Introduction

The 30^th^ annual conference of Society on Neuroimmune Pharmacology (SNIP) will take place from May 3^rd^-6^th^, 2026 at the Graduate Hotel by Hilton in Annapolis, Maryland. This location was chosen for its proximity to several major universities and institutions in Maryland, Washington, DC, and the Baltimore region, as well as to the National Institutes of Health (NIH) campuses. For those who need to fly to attend the conference, three major airports are available for the conference attendees to fly into this region, including the Thurgood Marshall Baltimore Washington International (BWI) Airport in Maryland, the Ronald Reagan National (DCA) Airport in Washington DC, and the Dulles International (IAD) Airport in Northern Virginia. Annapolis is a historical city with many points of interest, including the State Capitol of Maryland, which served as the second capital of United States (Dec 1776–Feb 1777), the U.S. Naval Academy, and Chesapeake Bay waterfront, featuring historic buildings, boating, fresh seafood (like crab cakes), and a charming downtown that is all within walking distance from our Conference hotel.

SNIP scientific conferences have traditionally focused on understanding and elucidating mechanisms of how various substance use disorders and infectious diseases, particularly human immunodeficiency virus-1 (HIV-1), impact the brain. In recent years, our society has also extended the topics into how these conditions impact the aging brain and how neurodegenerative disorders further intersect with these conditions. Emerging data suggests the intersections between dormant viral elements may be related to neurodegenerative disorders. This year, we received 185 abstract submissions. We organized a preconference workshop focusing on the Single Cell Opioid Responses in the Context of HIV (SCORCH) consortium. We have Dr. Nora Volkow, NIDA Director, as our keynote speaker, in addition to two memorial lectures, 11 exciting cutting-edge symposia and 115 poster presentations. We also invited Dr. Avindra Nath, NINDS Clinical Director, as our banquet speaker. In addition, the conference will include presentations that illustrate novel techniques, biomarkers, therapeutics or preventive strategies.

## Pre-Conference Workshop - Single Cell HIV and SUD Effects on the Brain: SCORCH Consortium Progress

This workshop will include investigators from the Single Cell Opioid Responses in the Context of HIV (SCORCH) consortium, which is funded by the National Institute on Drug Abuse (NIDA)/NIH, and was established in 2020. SCORCH is an interdisciplinary effort, involving teams of investigators at ten institutions – Yale, Boston University, the Ichan School of Medicine at Mount Sinai, the University of Nebraska Medical Center, the Broad Institute, University of California San Diego, Weill Cornell Medicine, The Allen Institute, Scripps Research, as well as a Data Coordination Center (DCC) hosted at the Institute for Genome Sciences, University of Maryland Baltimore School of Medicine (UMSOM). Samples collected and curated are shared amongst researchers in the field upon proper requests. This workshop will present the existing data resources and data analytic tools available for researchers outside the consortium, as well as highlight some of the exciting ongoing research by some of the SCORCH investigators. Specifically, Dr. John Satterlee, program officer for SCORH from NIDA/NIH, and seven speakers will show recent work using single-cell techniques to evaluate the persistence of HIV in the setting of drugs of abuse (e.g., cocaine, morphine). Dr. Owen White and Seth Ament from the Data Center at UMSOM will also present the available SCORCH datasets and analysis resources.

## Concurrent Preconference Symposium: “Catalyzing InterdisciplinaryResearch on HIV-Associated Co-occurring Conditions”

This symposium is organized and co-moderated by Dr. Rao Vasudev, PhD, NIMH/NIH and Kathleen Borgmann, PhD, NIDA/NIH (Speakers to be determined)

## The main conference from Sunday (May 3rd) evening through Wednesday (May 6th) afternoon

We organized 11 symposia, 3 named lectures, a banquet lecture, and two poster sessions.

Starting on Sunday evening, we will have a reception at a poster session that will include all the abstracts that were submitted for the Early Career Investigator Awards (ECITA); see abstracts 1-52 that follow the schedule below.

On Monday (5/4/26), we organized a keynote lecture by the NIDA Director, Dr. Nora Volkow, along with a memorial lecture, five symposia and a second poster session in the evening. Dr. Volkow’s lecture will discuss the *Neuroscience Research Priorities for NIDA’s HIV Research Program*. **Symposium 1** is titled “Dopamine Neurotransmission: New Frontiers in Intersystemic Signaling and Psychostimulant Regulation” and will be co-chaired by Habibeh Khoshbouei, PhD and Peter J. Gaskill, PhD. **Symposium 2** is the “Presidential Symposium: Neuroimaging Advances and A New Mouse Model for Research in HIV and Other Brain Disorders”, chaired by Linda Chang, MD, MS., will showcase novel imaging techniques as well as a new humanized mouse model. **Symposium 3** is the ECITA Symposium, with short presentations by 12 awardees, which is co-chaired by Jerel A. Fields, PhD. and Irma “Lisa” Cisneros, PhD. This is followed by the **Adarsh and Mahendra Kumar Memorial Lecture** by Dr. Amy Janes, who will present her work on “Neuroimaging Insights into Substance Use: A Focus on Nicotine Dependence”, with an introduction by Mark D. Namba, PhD. **Symposium 4** is titled “Implications of RNA Regulation in Neuroimmunological Responses”, which is chaired by Shiden Solomon, PhD. **Symposium 5** is titled “Glial mechanisms of NeuroHIV”, which is co-chaired by Ming-Lei Guo, PhD, MD, and Shao-Jun Tang, PhD. This first full day of scientific discovery will then continue but end with the second poster session that showcases the ongoing work by the SNIP members and their collaborators.

On Tuesday (5/5/26), we organized three more symposia and a banquet lecture. The first symposium is **Symposium 6** which will focus on “Noncanonical Roles of (Endo)lysosomes in the Central Nervous System”, co-chaired by Lindsay Festa, PhD, and Yisel Cantres Rosario, PhD. This is followed by the **Bill Narayan Memorial Lecture,** to be given by Jonathan D. Geiger, PhD, who will discuss how “Lysosomes Serve Important Roles in the Pathogenesis of Neurodegenerative Diseases, as well as the Safety and Efficacy of Modern Pharmacotherapeutics.” Dr. Geiger will be introduced by Norman J. Haughey, PhD. **Symposium 7** will present work related to the “Molecular Mechanisms of RNA Viruses- Induced Neurodegeneration”, which is co-chaired by Carlos Pardo, MD, and Pankaj Seth, PhD. **Symposium 8** will describe “Molecular Signatures of HIV Pathogenesis: Viral and Host Proteins and Therapeutic Frontiers”, co-chaired by Prasun K. Datta, PhD and Santhi Gorantla, PhD. The afternoon from 4-7 pm is free time for the attendees to meet up with colleagues or to explore the points of interest in Annapolis. We then have a banquet with a lecture by Avindra Nath, MD, who will present his exciting work on “Viruses and Neurodegenerative Diseases: Discovery of the enemies within.”

On Wednesday (5/6/26), the last day of the conference, we will have 3 more symposia and the SNIP Member Business Meeting. The first symposium is **Symposium 9** and is titled “From Stigma to Science: Unpacking HIV and Cannabis Use”, co-chaired by Barkha J. Yadav-Samudrala, PhD and Sylvia Fitting, PhD. This is followed by **Symposium 10**: SNIP Member Symposium, Co-chaired by Rick Noel, PhD and Susmita Sil, PhD, will include 9 short presentations from selected abstracts (with the highest scores) submitted by the SNIP members. This is followed by the SNIP Member Business Meeting, where we will receive reports from each committee, announce the newly elected officials, and briefly introduce next year’s conference location and plans. The last symposium of the conference will be **Symposium 11**, the “Local Symposium”, co-Chaired by Yajie Liang, PhD, and Amanda Brown, Ph.D, will highlight some of the ongoing work, including both clinical and preclinical research conducted by investigators from the local Baltimore area.

## Career development for Early Career Investigators (ECIs)

SNIP is proud to have always put a strong emphasis on supporting ECIs’ career development and provide the opportunities for them to come to SNIP. Members of SNIP are dedicated to mentoring and supporting the career development of ECIs. This is exemplified by the opening night of the conference, which includes the Early Career Investigator Poster Session and Social to highlight the work of the ECIs and promote networking. We also organized a symposium for the top 12 recipients of the ECI Travel Awards (ECITA). This year, due to the hard work and leadership of our Treasurer, Dr. Allison Andrews, our Past President, Dr. Howard Gendelman, and Dr. Sabita Roy, SNIP was awarded an R13 grant from the National Institute on Drug Abuse (NIDA)/NIH. A call for ECITA abstracts was conducted in December 2025, and the selection for the ECITA awardees was led by Dr. J. Adam Fields and the ECITA committee. The NIDA R13 grant (DA063455-01) provided funds for 22 ECITA Awardees. Twelve of these awardees (both predoctoral and postdoctoral investigators) will be symposium speakers at the ECITA symposium (Symposium 3); they will additionally present their work at the Poster Session on Sunday, while ten additional awardees will also present their work at the Poster Session. In addition to the funds provided to them to cover costs for their travel and lodging, these 22 awardees also received registration waivers. Furthermore, Dr. Fields will organize a “Meet-the-Mentor Lunch” for ECIs and trainees to meet with some of our society’s most successful and established scientists. At the luncheon, trainees can learn from senior investigators, ask questions about career paths or other challenges they may be facing, and potentially form collaborations with them. These events also provide outstanding opportunities for our trainees to seek potential post-doctoral or junior faculty positions.

## Dissemination of Conference Results in SNIP’s official journal Neuroimmune Pharmacology and Therapeutics (NIPT)

All abstracts from the symposia at this conference, including those from the speakers and the poster presenters, except for a few who requested that we not publish their abstracts since they are work-in-progress, are published in this special issue of NIPT (see abstracts that follow the schedule of the conference). All speakers and poster presenters are solicited and strongly encouraged to submit the work that they presented to the society’s official journal NIPT. This is an open access journal with rapid peer reviews. All published papers can be accessed on the internet and are available on PubMed Central (PMC) if the work is supported by NIH funds, as required. Under the exceptional leadership of our Editor-In-Chief, Dr. Howard E. Gendelman, who founded this journal for SNIP 2.5 years ago, this journal will certainly achieve a high impact factor soon, as he has done for the prior affiliated journal for SNIP, Journal of Neuroimmune Pharmacology, which he also founded 20 years ago. NIPT is published quarterly by De Gruyter. We have a managing editor, Amy Sather, and 13 other senior and section editors, along with a strong editorial board, all working together to shepherd this journal into a premier translational journal for cutting-edge research in four core areas: neuroscience, immunology, pharmacology, and therapeutics. Additionally, due to the unrelenting efforts by society leadership, our editors and devoted community leaders, we acquired considerable dollars from philanthropists to allow free publication of this open-access journal (until June 2027). SNIP will also receive some returns from the publication fees when we can collect fees for the open-access articles to support the society. This is an outstanding, unique opportunity for SNIP members and beyond to have their scientific work published quickly and in an open-access format, especially for our ECIs, who may have less grant funding to support their publications.

## Next SNIP Conference

The 31^st^ annual meeting of the SNIP will convene in Fort Lauderdale (tentative), Florida, in April or May, 2027, under the stewardship of President-Elect, Dr. Servio H. Ramirez, PhD. SNIP will continue to be the predominant scientific forum advancing understanding of neuroimmune mechanisms and treatments associated with HIV, substance use disorders, and their intersections with aging and neurodegenerative brain disorders. The 31^st^ SNIP conference will embrace a theme of innovation through team science and novel methodological approaches, recognizing that bold ideas and collaborative thinking drive transformative progress across the field. With guidance from the Meetings Committee, planned symposia will showcase how interdisciplinary strategies; including artificial intelligence and machine learning, collaborative team-science frameworks, material science, and tissue-engineered platforms, can be leveraged to address complex biological and therapeutic questions. Together, these efforts reflect SNIP’s ongoing commitment to fostering cross-disciplinary collaboration, scientific excellence, and a dynamic environment in which emerging concepts and technologies converge to shape the future of neuroimmune pharmacology.

**Table:** 

**Schedule for the 30**^**th**^ **Annual Society on Neuroimmune Pharmacology (SNIP) Conference**	
**Sunday, May 3^rd^. 2026**	
**12:00 – 5:30 PM**	**Registration Open**
**1:00 - 2:45 PM** **(Ballroom)**	**Concurrent Preconference Symposium: Single Cell HIV and SUD Effects on the Brain:SCORCH Consortium Progress** **Co-Moderators:** John Satterlee, Ph.D., NIDA/NIH, MD and Howard Fox, M.D., Ph.D., University of Nebraska Medical Center, Omaha, NE John Satterlee, PhD, Program Officer, NIDA, NIH **Overview of the SCORCH Consortium** Meng Niu, PhD, Assistant Professor, University of Nebraska Medical Center, Omaha, NE **Single-Nucleus Detection of Rare HIV-Infected Cells Defines the Cellular Landscape of HIV Persistence Iin the Human Brain.** Shilpa Buch, PhD, Professor, University of Nebraska Medical Center, Omaha, NE **Single-Cell Transcriptomic Profiling of Brain Regions in SIV-Infected and Cocaine Exposed Non-Human Primates** Xioake Xu, Ph.D., Research Associate, Computer Science and Artificial Intelligence Lab, Massachusetts Institute of Technology, Cambridge, MA Single-Cell Multiomic Dissection of HIV In the Context of Substance Use Disorder Across Multiple Brain Regions Hagen Tilgner, Ph.D., Associate Professor, Weill Cornell Graduate School of Medical Sciences, New York, NY **Single-Cell Long-Read Atlas of Hippocampal SIV infection, Antiretroviral Therapy and Chronic Morphine Exposure Reveals Cell-Type-Resolved Splicing Dysregulation**
**2:45-3:00 PM** **(Atrium)**	**Coffee Break**
**3:00 – 4:30 PM** **(Ballroom)**	Alyssa Wilson, Ph.D. Assistant Professor, Ichan School of Medicine at Mount Sinai, New York, NY **Changing Transcriptional Impacts of SUD & HIV Alone Vs. In Combination On Human Ventral Midbrain Neurons & Microglia** Owen White, MD, Professor, University of Maryland School of Medicine, Maryland, Baltimore, MD **The SCORCH Consortium Data Coordinating Center.** Seth Ament, PhD, Associate Professor, University of Maryland School of Medicine, Baltimore, MD
	**A Comparative Multi-Omic and Spatial Atlas for the Interacting Effects of HIV Infection and Substance Use in the Ventral Striatum of Humans, Macaques, Rats and Mice.** **Summary Discussion & Q&As**
**1:00 – 4:30 PM**	**Concurrent Preconference Symposium: Catalyzing Interdisciplinary Research on HIV-Associated Co-occurring Conditions** **Co-Moderators:** Rao Vasudev, PhD, NIMH/NIH and Kathleen Borgmann, PhD, NIDA/NIH (Speakers to be determined)
**4:30 – 5:30 PM** **(Atrium)**	**Break**
**5:30- 7:30 PM**	**Early Career Investigator Travel Award (ECITA) Poster Session (#1-#52) and Meet & Greet** **Dinner on your own**
**Monday, May 4^th^. 2026**	
**7:00-8:00 AM** **(Atrium)**	**Breakfast**
**8:00 AM– 5:00 PM**	**Registration**
**8:00 – 8:15 AM** **(Ballroom)**	**Welcome from the President:** Linda Chang, MD, MS, University of Maryland School of Medicine, Baltimore, MD
**8:15 – 9:45 AM** **(Ballroom)**	**Symposium 1: Dopamine Neurotransmission: New Frontiers in Intersystemic Signaling and Psychostimulant Regulation** **Co-Chairs:** Habibeh Khoshbouei, PhD, Professor and Vice Chair, University of Florida School of Medicine, and Peter J. Gaskill, PhD, Associate Professor, Drexel University College of Medicine Angela Carter, PhD, Assistant Professor, University of Alabama at Birmingham, Birmingham, AL **The Microbiome Controls Dopamine Levels in Response to Amphetamines via Regulation of the Dopamine Transporter** Peter J. Gaskill, PhD, Associate Professor, Drexel University College of Medicine, Philadelphia, PA **Dopamine Dysregulates Mitochondrial Metabolism in Microglia to Increase Inflammasome Activity** Habibeh Khoshbouei, PhD, Professor, University of Florida School of Medicine, Gainesville, FL **TNF-α Inhibition Attenuates Methamphetamine-Induced Dopamine Transmission and Self-Administration** George R. Uhl, MD, PhD, Chief of Neurology at the Baltimore VA Medical Center, Professor, University of Maryland School of Medicine, Baltimore, MD **Drugging PTPRD, A Novel Target for Stimulant Use Disorders**
**9:45 – 10:00 AM** **(Atrium)**	**Break**
**10:00 -11:30 AM** **(Ballroom)**	**Symposium 2: Presidential Symposium: Neuroimaging Advances and A New Mouse Model for Research in HIV and Other Brain Disorders** **Chair:** Linda Chang, MD, MS, Professor, University of Maryland School of Medicine, Baltimore, MD Beau Ances, MD, PhD, Daniel J Brennan Endowed Professor of Neurology, Washington University in Saint Louis, Saint Louis, MO **Decoding the Heterogeneity of HIV’s Impact on The Brain: The Power of Advanced Neuroimaging** Peiying Liu, PhD, Professor, University of Maryland School of Medicine, Baltimore, MD **Cerebrovascular Reactivity in Vascular Cognitive Impairment and Dementia (VCID) and in HIV-Associated Neurocognitive Disorders (HAND)** Ajay Verma, MD, PhD, General Partner, Formation Venture Engineering, Beverly, MA 01915 CEO, Twilight Bioscience, Beverly, MA **Intrathecal Drug Delivery: Imaging Insights for Neuroimmune Pharmacology** Zhang Chen, PhD, Postdoctoral Researcher and Howard Gendelman, MD, Chair and Professor, University of Nebraska Medical Center, Omaha, NE **Immune Transformation of Humanized CD4/CCR5/C1qbp/MHC Mice Facilitates Productive HIV-1 Infection**
**11:30-12:15 PM** **(Ballroom)**	**Plenary Keynote Lecture** **Introduction of speaker:** Linda Chang, MD, MS, Professor, University of Maryland School of Medicine, Baltimore, MD Nora Volkow, MD, Director of the National Institute on Drug Abuse, NIDA/NIH, Bethesda, MD **Neuroscience Research Priorities for NIDA’s HIV Research Program**
**12:15-1:15 PM** **(Atrium)**	**Lunch Break (on your own)**
**1:15 – 2:45 PM** **(Ballroom)**	**Symposium 3 – Early Career Investigator Travel Awardees (ECITA) – Also Presented as Posters** **Co-Chairs:** Jerel A. Fields, PhD, Associate Professor, University of California at San Diego, CA; Irma “Lisa” Cisneros, PhD, Assistant Professor UTMB, Galveston, TX 1. Agarwal Yash, BS, Dept. Pharmacology and Physiology, Drexel University College of Medicine **Dopaminergic Modulation of Viral Replication In HIV-Infected IPSC-Derived Organoids Exposed to Stimulant Drugs** 2. Aitizaz Ahsan, PhD, University of Nebraska Medical Center, Omaha, NE **Hydrogen Sulfide Rescues Microglia from HIV Tat-driven Ferroptosis: Implications for HIV-Associated Neuroinflammation** 3. Zainab Al Shakarchi, MS, University of Florida College of Medicine, Gainesville, FL **Development of a New Approach Method (NAM) to examine BBB integrity and ABC Efflux Transporter Activity and Expression in the Context of HIV Antiretroviral Therapy** 4. Shaurav Bhattariai, MS, University of Nebraska Medical Center, Omaha, NE **HIV-1 drives Alzheimer’s Disease Pathologies in APP Knock-In Mice** 5. Bhaskar Birru, PhD, University of Florida College of Medicine, Gainesville, FL **Development of the First Microphysiological System for The Study of the Blood-Cerebrospinal Fluid Barrier Interface** 6. Karthick Chennakesavan, PhD, Texas A&M University, TX **Chromatin Disruption and DNA Damage in HIV-Tat + Opioid Exposure: Protective Role of Dimethyl Fumarate** 7. Rahul K. Das, PhD, SUNY Buffalo, NY **Modeling HIV-Associated Neuroinflammaging: A Microphysiological Approach to Blood Brain Barrier Dysfunction** 8. Daniela Franco, M.A., University of Maryland School of Medicine, Baltimore, MD **Impact of Social Stress on Microglia-Neuron Interactions in the Nucleus Accumbens** 9. Sandesh Kamdi, PhD, University of Maryland School of Medicine, Baltimore, MD **Behavioral studies and In-Vivo Brain MR Spectroscopy in Human Microglia NOG-hIL34 mice** 10. Surendra Kumar, PhD, Johns Hopkins School of Medicine, Baltimore, MD **OPN as a Neuroimmune Link Between CNS Inflammation and Peripheral Bone Pathology in HIV Infection** 11. JR Ramirez, BS, University of Texas Medical Branch, Galveston, TX **Tunneling Nanotubes Mediate the Intercellular Spread of HIV-Gag-vRNA in Glial Cells Through a Myosin-X–Dependent Mechanism** 12. KA Schindler, BS, University of Miami, Coral Gables, FL **Combined EcoHIV and Methamphetamine Exposure Dysregulates Neuroimmune Responses Which Drives Cognitive and Neuropsychiatric Dysfunction**
**2:45- 3:00 PM** **(Atrium)**	**Break**
**3:00 – 3:45 PM** **(Ballroom)**	**Adarsh and Mahendra Kumar Lecture** **Introduction of Speaker:** Mark D. Namba, PhD, Postdoctoral Researcher, Department of Pharmacology & Physiology, Drexel University College of Medicine, Philadelphia, PA Amy Janes, PhD, Senior Investigator, Deputy Chief, Neuroimaging Research Branch, Intramural NIDA, NIH, Baltimore, MD **Neuroimaging Insights into Substance Use: A Focus on Nicotine Dependence**
**3:45 – 5:15 PM** **(Ballroom)**	**Symposium 4: Implications of RNA Regulation in Neuroimmunological Responses** **Chair:** Shiden Solomon, PhD, Postdoctoral Researcher, University of Pennsylvania, Philadelphia, PA Shan Zha, MD, PhD, Professor, Columbia University, New York, NY **RNA-Linked DNA Damage Responses in Immune Signaling** Anna G. Orr, PhD, Associate Professor, Weill Cornell Graduate School of Medical Sciences, New York, NY **Effects of TDP-43 Dysregulation on Astrocytes and Viral Infections** Yijing Su, PhD, Assistant Professor, University of Pennsylvania, Philadelphia, PA **Global Epitranscriptomic Alterations in HIV-Induced Monocyte-Derived Macrophages** Eliseo Eugenin, PhD, Professor, University of Texas Medical Branch, Galveston, TX **Residual Viral RNA Replication within CNS Viral Reservoirs Drive Chronic Bystander Neuronal and Glial Damage**
**5:15 – 5:30 PM** **(Atrium)**	**Break**
**5:30 – 7:00 PM** **(Ballroom)**	**Symposium 5: Glial mechanisms of NeuroHIV** **Co-Chairs:** Ming-Lei Guo, PhD, MD, Associate Professor, Old Dominion University, Norfolk, VA Shao-Jun Tang, PhD, Professor and Vice Chair for Research, SUNY-Stony Brook University School of Medicine, NY Woong-Ki Kim, PhD, Associate Director for Research, Professor, Tulane University School of Medicine, New Orleans, LA **Eradicating SIV from CNS Reservoirs by Targeting CSF1R Signaling.** Lena Al-Harthi, PhD, Chair and Professor, Vice Dean of Research, Rush Medical College, Chicago, IL
	**When Astrocytes Senesce: Functional Consequences for Brain Homeostasis in HIV and Methamphetamine Co-Morbidity.** Katherine Conant, MD, Professor, Georgetown University Medical Center, Washington, DC **CCR5 Antagonists to Treat Mood and Cognitive Deficits in HIV Infected Individuals.** Mark Maurelli, MS, Predoctoral Researcher, Department of Pharmacology and Anesthesiology, SUNY-Stony Brook University, Stony Brook, NY **Glial Contribution to Pathogenesis in The Pain Neural Circuits Induced by NRTIs**
**7:00 - 9:00 PM** **(Atrium)**	**General abstracts POSTER Session (#53-#115)**
**7:30 -9:00** **Location (TBD)**	**NeuroImmune Pharmacology and Therapeutics (NIPT) Editors’ Dinner**
**Tuesday, May 5^th^, 2026**	
**7:00-8:00 AM** **(Atrium)**	**Breakfast**
**8:00 AM– 5:00 PM**	**Registration**
**8:00 – 9:30 AM** **(Ballroom)**	**Symposium 6: Noncanonical Roles of (Endo)lysosomes in the Central Nervous System** **Co-Chairs:** Lindsay Festa, PhD, Assistant Professor, Children’s Hospital of Philadelphia, Philadelphia, PA Yisel Cantres Rosario, PhD, Assistant Professor, University of Puerto Rico, San Juan, PR Sandra Maday, PhD, Associate Professor, Perelman School of Medicine University of Pennsylvania, Philadelphia, PA **TRPML1 Controls Lysosome Positioning to Shape Astrocyte Morphology** Mable Lam, PhD, Postdoctoral Fellow, Stanford University, Palo Alto, CA **Role of Exocytosis in Myelin Membrane Expansion and Plasticity.** Lindsay Festa, PhD, Assistant Professor, Children’s Hospital of Philadelphia, Philadelphia, PA **The Lysosome Is A Regulator of The Oligodendrocyte Cytoskeleton.** Andrew Arrant, PhD, Assistant Professor, University of Alabama at Birmingham, Birmingham, AL **Progranulin Exerts Neurotrophic Effects by Acting in Lysosomes of Neurons and Glia**
**9:30-10:15 AM** **(Ballroom)**	**Bill Narayan Memorial Lecture** **Introduction of Speaker:** Norman J. Haughey, PhD, Professor, Tulane University School of Medicine, New Orleans, LA Jonathan D. Geiger, PhD, Chester Fritz Distinguished Professor, Univ. of North Dakota, Grand Forks, ND **Lysosomes Serve Important Roles in the Pathogenesis of Neurodegenerative Diseases, as well as the Safety and Efficacy of Modern Pharmacotherapeutics**
**10:15 – 10:30 AM** **(Atrium)**	**Break**
**10:30—12:00 PM** **(Ballroom)**	**Symposium 7: Molecular Mechanisms of RNA Viruses Induced Neurodegeneration** **Co-Chairs**: Carlos Pardo, MD, Professor, Johns Hopkins University School of Medicine, Baltimore, MD Pankaj Seth, PhD, Senior Professor, National Brain Research Center, Manesar, India Lisa Henderson, PhD, Scientist, Section of Infections of the Nervous System, NINDS, NIH **Antisense oligonucleotides as broadly effective inhibitors of mosquito-borne flaviviruses.** Tory P. Johnson, PhD, Assistant Professor, Johns Hopkins School of Medicine, Section of Infections of the Nervous System, NINDS. NIH **KCNA10 Autoantibodies and Endothelial Injury in Post-COVID Autonomic Dysfunction.** Carlos Pardo, MD, Professor of Neurology, Johns Hopkins School of Medicine, Baltimore, MD **Role of neuroimmune factors in the pathogenesis of neuroinflammatory disorders.** Pankaj Seth, PhD, Senior Professor at National Brain Research Centre, Manesar, India **Molecular mechanisms of Zika virus induced CNS pathogenesis using 2D and 3D models of human brain cells.**
**12:00 -1:00 PM**	**Lunch on your own or Meet-the-Mentor Lunch (by invitation only)**
**1:00 – 1:45 PM**	**Discussions and Q & A with NIH Program Officers**
**1:45 – 3:15 PM** **(Ballroom)**	**Symposium 8: Molecular Signatures of HIV Pathogenesis: Viral and Host Proteins and Therapeutic Frontiers; Co-Chairs**: Prasun K. Datta, PhD, Associate Professor, Tulane University, and Tulane National Primate Research Center, Covington, LA; Santhi Gorantla, PhD, Professor, University of Nebraska Medical Center, Omaha, NE Dianne Langford, PhD, Dean and Associate Vice Chancellor, Virtua Health College of Medicine and Life Sciences at Rowan University, NJ. **Spatial proteomic signatures of HIV in the human frontal cortex are associated with neurocognitive performance.** Chandravanu (CV) Dash, PhD, Chair and Professor, Meharry Medical College, Nashville, TN
	**HIV-1 integration and capsid-binding host factors: Who, When and How!** Lori A. Emert-Sedlak, Ph.D., Associate Professor, University of Pittsburgh School of Medicine, Pittsburgh, PA **Small Molecule Inhibitors of the HIV-1 Nef Virulence Factor as a New Approach to HIV Therapy.** Demetra P. Kelenis, PhD, Post-Doctoral Fellow, Columbia University, New York, NY **HIV-1 infection Induces Vif-Driven SUMOylation of Host RNA Splicing Factors Mediating Proper Viral RNA Splicing.**
**3:15 – 6:30 PM**	**Free Time**
**6:30 – 9:00 PM** **(Ballroom)**	**Banquet** **Introduction of speaker:** Dr. Linda Chang, , MD, MS, Professor, University of Maryland School of Medicine, Baltimore, MD Avindra Nath, MD, Clinical Director, NINDS, Senior Investigator, Section of Infections of the Nervous System, Division of Neuroimmunology and Neurovirology, NINDS, NIH **Viruses and Neurodegenerative Diseases: Discovery of the Enemies Within**
**Wednesday, May 6^th^, 2026**	
**7:00 – 8:00 AM**	**Breakfast**
**8:00 – 9:30 AM** **(Ballroom)**	**Symposium 9: From Stigma to Science: Unpacking HIV and Cannabis Use** **Co-Chairs:** Barkha J. Yadav-Samudrala, PhD, Research Associate, and Sylvia Fitting, PhD, Professor, University of North Carolina, Chapel Hill, NC Samantha M. Ayoub, PhD, Postdoctoral Researcher, University of California San Diego, San Diego, CA **Delineating the Impact of Phytocannabinoid Exposure on HIV-Associated Neurocognitive Impairment: Insights from The HIV-1 Transgenic Rat Model.** Alysha Ellison, PhD, Postdoctoral Researcher, Emory University, Atlanta, GA **Real World Considerations for Cannabinoid Based Therapies During HIV** Edward P. Browne, PhD, Associate Professor, University of North Carolina, Chapel Hill, NC **Impact of Cannabis Use on the Viral Reservoir and Immune Cell Gene Expression in People with HIV on Antiretroviral Therapy.** Mahesh Mohan, DVM, MS, PhD, Professor, Texas Biomedical Research Institute, San Antonio, TX **The Gut-Brain Connection: How Phytocannabinoids Supplement HIV Treatment to Reduce Chronic Inflammation.**
**9:30 - 9:45 AM** **(Atrium)**	**Break**
**9:45 – 11:15 AM** **(Ballroom)**	**Symposium 10: SNIP Member Symposium** **Co-Chairs:** Susmita Sil, PhD, Assistant Professor, University of Nebraska, Medical Center, Omaha, NE and Richard J. Noel, PhD., Chair and Professor, Ponce Health Sciences University, Ponce, PR Amber Virdi, PhD, Dept. Microbial Pathogens and Immunity, Rush University Medical Center, Chicago, IL **HIV Suppresses Colonic B-Catenin, Alters the Microbiome, and Induces Gut Barrier Leakiness That Is Recapitulated by the Microbiome Independent of HIV and Reversed by B-Catenin Activation** Allison Andrews, PhD, Dept. Pathology, Immunology and Laboratory Medicine, University of Florida, Gainesville, FL **Chronic HIV Infection Alters Neuronal Firing and Neurovascular Coupling in Reward Pathway Relevant Areas in Awake-Behaving Animals** Mark Namba, PhD, Dept. Pharmacology and Physiology, Drexel University College of Medicine, Philadelphia, PA **EcoHIV Infection Impairs Extinction Learning and Dysregulates Corticostriatal Microglia** Xuesong Chen, PhD, Dept. Biomedical Sciences, University of North Dakota School of Medicine and Health Sciences, Grand Forks, ND **Role of Endolysosomes in SARS-CoV-2 Spike-Induced Cellular Senescence in Human Astrocytes.** Sudipta Ray, PhD, Dept. Pharmacology and Experimental Neuroscience, University of Nebraska Medical Center, Omaha, NE **HIF-1 siRNA encapsulated Extracellular Vesicle therapy protects against HIV-associated neurological deficits.** Jimmy Olusakin, PhD, Dept. Neurobiology, University of Maryland School of Medicine, Baltimore, MD **Perinatal Fentanyl Exposure Reprograms Microglial Development and Neuroimmune Signaling Across Mesocorticolimbic Circuits** David Ajasin, PhD, Dept. Internal Medicine, University of Texas Medical Branch, Galveston, TX
	**SIV/HIV-Induced Lipid Dysregulation Induce Neuroinflammation and Tissue Damage, and the Role of Pannexin-1 in Neuro-HIV** Peter Halcrow, PhD, Dept. Psychiatry, University of California San Diego, San Diego, CA **HIV-relevant Inflammatory Stimuli and Antiretroviral Therapy Exposure Induces Reactive Astrocytes Driven by Glycolysis and Resulting in the Secretion of Neurotoxic Compounds.** João Mamede, PhD, Dept. Microbial Pathogens and Immunity, Rush University Medical Center, Chicago, IL **Methamphetamine and HIV-1 Infection Activate Innate Sensing in Microglia Through the Inflammatory cGAS-STING pathway**
**11:15 AM–12:15 PM** **(Ballroom)**	**SNIP Business Meeting for Members**
**12:15 – 1:15 PM**	**Lunch on Your Own**
**1:15 – 2:45 PM** **(Ballroom)**	**Symposium 11: Local Organizing Committee Symposium** **Co-Chairs:** Yajie Liang, PhD, Assistant Professor, University of Maryland School of Medicine, Baltimore, MD Amanda Brown, PhD, Associate Professor, Johns Hopkins University School of Medicine, Baltimore, MD Ze Wang, PhD, Professor, Center for Advanced Imaging Research, University of Maryland School of Medicine, Baltimore, MD **Cerebral Perfusion as a Biomarker for Alzheimer’s Disease.** Yajie Liang, PhD, Assistant Professor, Center for Advanced Imaging Research, University of Maryland School of Medicine, Baltimore, MD **Intravital Imaging Microglia Dynamics in the Live Mouse Brain** Mary Kay Lobo, PhD, Professor, Co-Director Center for Substance Use in Pregnancy, Associate Director for Kahlert Institute for Addiction Medicine, University of Maryland School of Medicine, Baltimore, MD **Reward Circuitry Microglia-Neuron Interactions across the Lifespan in Disrupted Motivation** Alonso Heredia, PhD, Professor, Institute of Human Virology, University of Maryland School of Medicine, Baltimore, MD **Humanized Mouse Models that Enable the Development of Human Myeloid Cells: Opportunities for Targeting HIV Reservoirs in the Brain** Walter Royal, MD, Endowed Professor of Brain Science, Center for Brain Health Research, Morgan State University, Baltimore, MD **Modeling Neural Degeneration to Enhance Brain Health in HAND**
**End of Conference**	

## Concurrent Pre-Conference Symposium - Catalyzing Interdisciplinary Research on HIV-Associated Co-occurring Conditions

**Co-moderators:** Rao Vasudev, PhD NIMH/NIH; Kathleen Borgmann, PhD, NIDA/NIH (Speakers to be determined)

## Concurrent Pre-Conference Workshop - Single Cell HIV and SUD Effects on the Brain: SCORCH Consortium Progress

**Co-Moderators:** John Satterlee, Ph.D., NIDA/NIH, MD and Howard Fox, M.D., Ph.D., University of Nebraska Medical Center, Omaha, NE

**Speakers:**

John Satterlee, PhD, Health Science Administrator (Epigenetics), NIDA, NIH

Meng Niu, PhD, Assistant Professor, University of Nebraska Medical Center, Omaha, NE

Shilpa Buch, PhD, Professor, University of Nebraska Medical Center, Omaha, NE

Xiaoke Xu, PhD, Postdoctoral Associate, Massachusetts Institute of Technology, Cambridge, MA

Hagen Tilgner, Ph.D., Associate Professor, Weill Cornell Graduate School of Medical Sciences, New York, NY

Alyssa Wilson, Ph.D. Assistant Professor, Ichan School of Medicine at Mount Sinai, New York, NY

Owen White, MD, Professor, University of Maryland School of Medicine, Baltimore, MD

Seth Ament, PhD, Associate Professor, University of Maryland School of Medicine, Baltimore, MD

## Single-Nucleus Detection of Rare HIV-Infected Cells Defines the Cellular Landscape of HIV Persistence in the Human Brain

Niu, MN, PhD^1^, Cai, YC, PhD^2^, Jacobs, NJ, PhD^3^, Gabuzda, DH, PhD^4^, Corley, MC, PhD^5^, Herb, BH, PhD^6^, Volsky, DV, PhD^7^, Receveur, JR, PhD^6^, Matkhanov, DM, PhD^1^, White, OW, PhD^6^, Kluger, YK, PhD^3^, Cheng, CC, PhD^2^, Fox, HF, MD, PhD^1^; ^1^Department of Neurological Sciences, University of Nebraska Medical Center, OMAHA, NE 68198 ^2^Department of Psychiatry, University of California San Diego, La Jolla, CA 92093 ^3^Department of Pathology, Yale University, New Haven, CT 06520 ^4^Dana-Farber Cancer Institute , Harvard University, Boston, MA 02215 ^5^Department of Medicine, University of California San Diego, La Jolla, CA 92093 ^6^Institute for Genome Sciences , University of Maryland School of Medicine, Baltimore, MD 21201^7^Icahn School of Medicine at Mount Sinai, Icahn School of Medicine at Mount Sinai, New York City, NY 10029.

HIV-1 enters the central nervous system early after infection and establishes a long-lived reservoir that persists despite antiretroviral therapy. Single-cell and single-nucleus RNA sequencing enable analysis of HIV infection in the human brain, yet sensitive methods for detecting rare, infected cells remain limited. We present a scalable multi-reference framework for identifying HIV RNA–positive cells in human CNS single-nucleus RNA-seq data by integrating a modified HIV reference genome, subject-specific variant-updated references, and a comprehensive HIV strain collection. We applied this framework to 250 post-mortem brain samples from the SCORCH consortium spanning 12 brain regions and 102 donors. After screening, 48 samples comprising 559,207 high-quality nuclei were analyzed. We identified 1,939 HIV RNA–positive cells exclusively in samples from people with HIV and retained 908 high-confidence infected cells using conservative thresholds. HIV-positive cells were rare overall and enriched in cases with HIV encephalitis. Microglia represented the predominant infected population, with smaller contributions from oligodendrocytes, astrocytes, and neurons. These results establish a harmonized approach for detecting rare HIV-infected cells and identify microglia as the dominant HIV-infected population in the human brain.

Supported by NIH/NIDA (5U01DA053624-05)

## Single Cell Transcriptomic Profiling of Brain Regions in SIV-Infected and Cocaine Exposed Nonhuman Primates

Buch, S^1^, Deshetty, UM^1^, Callen, S^1^, Periyasamy, P^1^, Rana, T^2^; ^1^Department of Pharmacology and Experimental Neuroscience, University of Nebraska Medical Center, Omaha, NE 68198 ^2^Department of Cellular and Molecular Medicine, University of California San Diego, La Jolla, CA 92093.

HIV infection and cocaine abuse frequently co-occur and synergistically worsen neurocognitive impairment, yet their combined effects on brain cell-specific regulatory programs remain poorly defined. Objective: We hypothesize that cocaine alters gene expression, chromatin accessibility, and viral persistence in key brain cell types, particularly myeloid cells, thereby exacerbating SIV/HIV-associated neuropathogenesis despite suppressive cART. Methods: Using a controlled nonhuman primate model, we studied three groups of rhesus macaques: (1) uninfected controls, (2) SIV-infected animals on cART, and (3) SIV-infected animals on cART with cocaine exposure. Animals were inoculated with SIVmac251, maintained on suppressive therapy, and monitored longitudinally. At necropsy, vulnerable brain regions (anterior cingulate gyrus, putamen, amygdala, hippocampus) were processed for paired single-nucleus RNA-seq and ATAC-seq analyses. Results: We expect to achieve robust quality control metrics, high nuclei yield, accurate cell-type annotation, and cell-specific RNA-seq gene expression and networks of potential significance. Enhanced glial activation and neuroinflammatory signatures are also expected in SIV/cocaine versus SIV alone animals. Conclusion: This study will define how cocaine and SIV/cocaine interact to reshape brain regulatory landscapes and drive persistent neuropathogenesis.

Supported by National Institute on Drug Abuse (NIDA), NIH/U01DA058402.

## Single-Cell Multiomic Dissection of HIV In the Context of Substance Use Disorder Across Multiple Brain Regions

Xu, X.X., PhD^1^, Liu, Z.L., PhD^1^, Galani, K.G., PhD^1^, Linville, R.M.L., PhD^2^, James, B.T.J., PhD^1^, Mangan, R.J.M., PhD^1^, Grayson, A.G., PhD^1^, Fass, S.B.F., BS^1^, Zhang, S.Z., PhD^1^, Ho, L.-L.H., PhD^1^, Heiman, M.H., PhD^2^, Gabuzda, D.H.G., PhD^3^, Kellis, M.K., PhD^1^; ^1^Computer Science and Artificial Intelligence Lab, Massachusetts Institute of Technology, Cambridge, MA, 02139 United States. ^2^Picower Institute for Learning and Memory, Massachusetts Institute of Technology, Cambridge, MA, 02139 United States. ^3^Dana-Farber Cancer Institute, Harvard Medical School, Boston, MA, 02215 United States.

HIV infection and opioid use disorder (OUD) exert substantial effects on the CNS, yet their cell type- and region-specific impacts remain incompletely defined. We performed a large-scale single-cell multiomic analysis across four brain regions, the frontal cortex, insular cortex, putamen, and nucleus accumbens, using postmortem samples from 89 individuals stratified by HIV and OUD status. More than 3 million nuclei were profiled by single-nucleus multiomic sequencing (snMultiome, snRNA-seq+snATAC-seq), enabling integrative characterization of transcriptional and chromatin accessibility landscapes. Cells were classified into 8 major classes, 32 subclasses, and 61 subtypes, providing a high-resolution atlas of cortical and striatal cell populations. Cell-subtype-resolved transcriptomic analyses identified selectively vulnerable glial populations and revealed region-dependent alterations associated with HIV and OUD. Integrative regulatory network analyses uncovered dysregulated transcriptional programs and disrupted molecular interactions between immune and neuronal populations, highlighting coordinated changes in neuroimmune signaling. Gene co-expression network analysis further identified modules strongly associated with cognitive status in people with HIV, implicating specific pathways linked to neurodegenerative processes. Collectively, this study delineates the cellular heterogeneity and molecular circuitry underlying HIV- and OUD-associated neuropathology across multiple brain regions.

Supported by NIDA U01DA053631

## Single-Cell Long-Read Atlas of Hippocampal SIV Infection, Antiretroviral Therapy and Chronic Morphine Exposure Reveals Cell-Type-Resolved Splicing Dysregulation

Tilgner, HU, PhD^1^; J Hsu*^1^, W Hu*^1^, M Corley*^1^, S Buch*^2^, J Jarroux1, S Callen2, Y He1, C Foord1, A Joglekar1, A Pang1, S Pollard1, M Parsons^1^, S Bowler^1^, H Fox+^2^, TA Milner+^1^, LC Ndhlovu+^1^, HU Tilgner+^1^. ^1^Weill Cornell Medicine, New York, NY 10021 ^2^University of Nebraska Medical Center, University of Nebraska Medical Center, Omaha, NE 68198.

Alternative splicing generates transcriptomic diversity in the brain and shapes cell-type–specific regulatory programs. For HIV, opioid exposure, and antiretroviral therapy (ART), prior studies often focus on the gene level, leaving splicing defects unresolved. We profile single-cell isoforms from macaque hippocampus upon SIV infection with/without ART, with/without chronic morphine exposure. We find >100 splicing events dysregulated by SIV, morphine, or their combination across neurons and glia. In contrast to similar work in Alzheimer’s, SIV-induced splicing defects are of similar magnitude across neuron types and oligodendrocytes. In most brain cell types, most SIV-associated splicing dysregulation is rescued by ART. Strikingly, microglia show only a limited rescue effect. Importantly, a microglial sub-population, associated with activation of interferon-stimulated genes harbors SIV-induced splicing dysregulation. Although ART rescues many SIV-dysregulated exons, it also produces widespread ART-specific splicing changes, including cryptic exon inclusion. These RNA defects occur on exons similarly used in human hippocampi. Morphine-induced splicing changes often converge with SIV on shared exons. Our findings establish a foundation for potentially complementing ART – and highlight the importance of splicing in HIV, ART and drug research in the brain.

Supported by NIDA U01DA053625.

## Changing Transcriptional Impacts of SUD & HIV Alone Vs. In Combination on Human Ventral Midbrain Neurons & Microglia.

Wilson, AM, PhD^1^, Jacobs, MM, PhD^2^, Lambert, TY, BS^3^, Valada, A, BS^4^, Meloni, G, MS^2^, Gilmore, E, MS^2^, Murray, J, BS^2^, Morgello, S, MD^5^, Akbarian, S, MD, PhD^6^; ^1^Depts. of Neurology & Psychiatry, Icahn School of Medicine at Mount Sinai, New York, NY 10029 ^2^ Department of Neurology, Icahn School of Medicine at Mount Sinai, New York, NY 10029 ^3^Graduate Division of Biomedical Sciences, Albert Einstein College of Medicine, New York, NY 10461 ^4^MD Program, Albert Einstein College of Medicine, New York, NY 10461 ^5^Depts. of Neurology, Neuroscience, & Pathology, Molecular and Cell Based Medicine, Icahn School of Medicine at Mount Sinai, New York, NY 10029 ^6^Depts. of Psychiatry, Neuroscience, & Genetics and Genomic Sciences, Icahn School of Medicine at Mount Sinai, New York, NY 10029.

For people with HIV (PWH), substance use disorders (SUDs) are a prominent neurological risk factor, and the impacts of both conditions on dopaminergic pathways are a potential point of deleterious convergence. Here, we profile, at single-nucleus resolution, the substantia nigra (SN) transcriptomes of 90 postmortem donors in the context of chronic HIV and opioid/cocaine SUD, including 67 prospectively characterized PWH. We discuss transcriptional changes in SN neurons and microglia due to HIV, distinguishing between controlled and uncontrolled infection, and separately, to SUD; these include alterations to SN dopaminergic neuron function and altered expression of hundreds of microglial pro- and anti-inflammatory regulators. Further, with dual SUD/HIV diagnosis, we find stepwise, progressive microglial dysregulation coupled to altered SN dopaminergic and GABAergic signaling, with further progression in uncontrolled HIV (characterized by lack of viral suppression in blood). We discuss notable examples from our findings, including that in virologically suppressed donors, SUD comorbidity was associated with microglial transcriptional changes permissive for HIV infection, as well as emergence of additional transcriptional signatures consistent with selective vulnerability of SN dopamine neurons. We also report HIV-related downregulation of monoamine reuptake transporters specifically in dopaminergic neurons regardless of SUD status or viral load.

Supported by NIH U01DA053600, R61DA048207, R01NS108801, RFAG060961, U24MH100931, 75N95023C00015; ORIP S10OD026880, S10OD030463; NCATS UL1TR004419.

## The SCORCH Consortium Data Coordination Center

White, O, PhD, Chang, Linda, MD, Receveur, Joseph, P, PhD, Giglio, Michelle, Michelle, PhD, Mahurkar, Anup, A., MS, Herb, Brian, R, PhD, Lobo, Mary Kay, PhD, Ament, Seth A; ^1^Institute for Genome Sciences, University of Maryland, Baltimore School of Medicine, Baltimore, MD 21201.

We have established a data coordination center to analyze single-cell and other molecular datasets generated by the Single Cell Opioid Responses in the Context of HIV (SCORCH) consortium. This project leverages the Neuroscience Multi-Omic Data Archive (NeMO; nemoarchive.org), which hosts much of the BRAIN Initiative’s multi-omic data, and integrates several existing data management, integration, and web presentation tools developed by the University of Maryland group. The SCORCH consortium studies substance use disorders (SUD) and drug addiction, which are major threats to public health, impacting not only the millions of individuals living with SUD but also their families and communities. A central challenge in studying and treating addiction in human populations is the high prevalence of comorbid conditions, including an increased risk of human immunodeficiency virus (HIV) infection. Of the approximately 15 million people who inject drugs globally, 17% are living with HIV. Conversely, HIV itself is a risk factor for SUD: chronic pain syndromes, which are common among individuals with HIV, can lead to increased use of opioid pain medications and, in turn, elevate the risk of opioid addiction. Our team has developed a web-based data archive (scorch.igs.umaryland.edu) to search and access single-cell genomic datasets, along with a complementary data visualization and analysis platform (nemoanalytics.org). The SCORCH datasets and analysis resources will be presented.

Supported by NIH-NIDA 2UM1DA052244.

## A Comparative Multi-Omic and Spatial Atlas for The Interacting Effects of HIV Infections and Substance Use in The Ventral Striatum of Humans, Macaques, Rats, And Mice

Ament, SA, PhD; Institute for Genome Sciences, University of Maryland School of Medicine, Baltimore, MD 21201.

The nucleus accumbens (NAc) mediates rewarding properties of addictive substances and undergoes lasting changes in substance use disorders (SUDs). The NAc is structured into discrete functional domains with medium spiny neurons (MSNs) projecting topographically to downstream brain regions. However, the molecular diversity, spatial organization, and functional adaptations of MSNs in SUDs remain elusive. To address this, we constructed a comparative multi-omic and spatial atlas of the NAc in humans, macaques, rats, and mice, integrating single-cell multi-omic and spatial transcriptomic data from over one million NAc cells. Our analyses revealed deep evolutionary conservation of many MSN subtypes, as well as human- and primate-specific specializations. Projection mapping revealed previously unrecognized, topographically arranged MSN projections to brain regions regulating reward and aversion. In humans, the dynamics of MSN subtype-specific gene networks reflected complex patterns of polysubstance use that are inherent to SUDs. Comparisons to animal models enabled us to link SUD-associated gene networks to specific substances and stages of addiction. Selective manipulation of these gene networks in mice altered opioid-related behaviors, offering potential therapeutic targets. This work provides a blueprint for identifying key molecular and circuit mechanisms underlying addiction and developing targeted interventions.

Supported by NIDA/NIH - 2UM1DA052244

## Monday, May 4^th^, 2026

### Symposium 1: Dopamine Neurotransmission: New Frontiers in Intersystemic Signaling and Psychostimulant Regulation

#### The microbiome controls dopamine levels in response to amphetamines via regulation of the dopamine transporter

Carter, A, PhD, Assistant Professor, University of Alabama at Birmingham,

(Abstract is not published per author request)

#### Dopamine dysregulates mitochondrial metabolism to increase NLRP3 inflammasome activity

Gaskill, PJ, PhD^1^, Daniali, M, MS^1^, Channer, B, PhD^1^; ^1^Department of Pharmacology and Physiology, Drexel University College of Medicine, Philadelphia, PA, 19102 United States.

Despite its canonical role as a neurotransmitter, increasing evidence shows that dopamine also acts as an immunomodulator. Most immune cell types, including myeloid cells, express dopamine receptors and transporters. We and others have shown that dopamine regulates immune processes such as phagocytosis, cytokine production and inflammasome activation. However, the specific signaling mechanism(s) by which dopamine regulates these processes remains opaque. Prior data show that dopamine levels induced by stimulant use increase NF-κB activity, enhance NLRP3 inflammasome activation and lead to a greater release of inflammatory cytokines. In this study, we show that dopamine mediates these effects, at least in part, by disrupting mitochondrial activity. Specifically, acute dopamine interaction leads to mitochondrial depolarization, and over time increases fission by increasing the phosphorylation of DRP-1 and the production of mitochondrial superoxide (MitoSOX). This drives the release of mitochondrial DNA (mtDNA) into the cytoplasm, activating the cGAS-STING pathway and increasing NF-Î°B nuclear translocation. This in turn leads to the activation of the NLRP3 inflammasome, as well as promoting the release of cytokines such as IL-6. These effects are blocked by inhibition of NF-κB, cGAS, or DRP1-mediated mitochondrial fission. These findings identify a previously unrecognized mechanism through which dopamine drives mitochondrial dysfunction and cGAS-STING-dependent inflammation in human macrophages, revealing new dimensions of dopaminergic control over innate immune responses.

Supported by National Institutes on Drug Abuse

#### TNF- α Inhibition Attenuates Methamphetamine-induced Dopamine Transmission and Self-Administration

Khoshbouei, HK; Dept of Neuroscience, University of Florida, Gainesville, FL, 32611 United States.

The highly addictive psychostimulant methamphetamine increases the release of dopamine in the brain’s reward circuitry, where it also promotes the release of cytokines, including TNF-α, that contribute to neuroinflammation associated with methamphetamine abuse. Here, we found a dynamic interplay between methamphetamine and TNF- α in facilitating dopamine transmission within the ventral tegmental area (VTA) in mice. In ex vivo mouse brain slices and dopaminergic neurons, methamphetamine or TNF-α treatment increased dopamine release, intracellular Ca2+ concentrations, and the firing activity of VTA dopaminergic neurons. These effects depended on the activity of dopamine transporter (DAT) and L-type voltage-gated Ca2+ channels. Pharmacological inhibition of either DAT or TNF-α signaling mitigated these effects, suggesting that methamphetamine-induced alterations in VTA dopaminergic neurons are partially TNF-α dependent. These results underscore the role of neuroimmune signaling in modulating the dopaminergic circuitry and may inform therapeutic strategies for addressing methamphetamine addiction and its associated neuroinflammatory disorders.

Supported by NIDA

#### Drugging PTPRD, a Novel Target for Stimulant Use Disorders

Uhl, GR, MD, PhD; Dept of Neurology, University of Maryland School of Medicine, Baltimore, MD, 21201 United States.

Relapse, often following self-administration of “lapse” doses and often during the initial weeks of attempted abstinence, is a major problem that limits the efficacy of treatment for stimulant use disorders. Variation in the gene encoding the receptor type protein tyrosine phosphatase D (PTPRD) is associated with many addiction-related phenotypes and levels of PTPRD expression in postmortem brains. Mice with life-long reductions in PTPRD expression display reduced reward from stimulants, validating human genetic and motivating searches for pharmacologic PTPRD inhibitors. We have identified lead and developmental compound PTPRD inhibitors, 7-BIA pentilludin (NHB1109). Neither compound displays any activity in NIDA-EUROFINS screens of targets of currently available drugs. Pentilludin displays improved potency (690 nM) and improved specificity re the closest relative, PTPRS. Acute administration of each of these compounds reduces stimulant reward at doses that are modest fractions of the no observed adverse effect levels. Pentilludin displays no disqualifying activities in hERG, Ames or micronucleus tests, has 2-hour pharmacological half-life in rats and dogs, acceptable oral bioavailability and dose limiting reversible proteinuria (rats) and hepatotoxicity (dogs) at doses >> those that reduce amphetamine self-administration. Advancement to use in humans will allow us to assess pentilludin’s ability to reduce reward from stimulants and to evaluate the efficacy of this reward reduction in reducing relapse to stimulant use in treatment-seekers with stimulant use disorders.

Supported by NIDA

### Symposium 2: Presidential Symposium

#### Advances in Neuroimaging and A New Mouse Model for Research in HIV and Other Brain Disorders

**Chair:** Linda Chang, MD, MS, Professor, University of Maryland School of Medicine

##### Decoding the Heterogeneity of HIV’s Impact on The Brain: The Power of Advanced Neuroimaging

Ances, Beau, MD, PhD, Daniel J Brennan Endowed Professor of Neurology, Department of Neurology, Washington University in Saint Louis, Saint Louis, MO 63110.

This talk will delve into the crucial role of advanced neuroimaging techniques in identifying and evaluating changes in the brain among people with HIV (PWH). Diagnosing and managing PWH has long been challenging and need to be reexamined in the context of evolving co-morbidities, social determinants of health, and the aging process. Recent advancements in neuroimaging offer promising tools to better understand the heterogeneity among PWH. By employing machine learning algorithms on large datasets, researchers can identify distinct biological subtypes, facilitating the implementation of precision medicine. This capability is especially vital for older PWH, as advanced neuroimaging techniques can be instrumental in differentiating Alzheimer’s disease from cognitive impairments caused by HIV. By integrating imaging data with biofluid analysis and cognitive testing, this talk will help highlight specific biological subtypes. These insights will pave the way for tailored interventions, ultimately improving the quality of life for PWH

Supported by NIH.

##### Cerebrovascular Reactivity (CVR) MRI as A Biomarker for Vascular Cognitive Impairment and Dementia (VCID) and HIV-Associated Neurocognitive Disorders (HAND)

Liu, Peiying, PhD; Professor, Department of Diagnostic Radiology & Nuclear Medicine, University of Maryland School of Medicine, Baltimore, MD 21201.

Cerebral small vessel disease (SVD) related vascular contributions represent a major factor contributing to cognitive decline and dementia (VCID) in older adults. However, there has not been a validated biomarker for the diagnosis and treatment monitoring of this condition. Cerebrovascular reactivity (CVR), the ability of cerebral small vessels to dilate upon stimulus, is thought to directly reflect the physiological function of the brain microvasculature. Our single-site study in 69 subjects suggested that CVR was associated with the global cognitive function measured by the Montreal Cognitive Assessment (MoCA) after adjusting for age, sex, and education. The following multi-site validation study of a total of 264 older participants from three sites further showed that the association between CVR and global cognition was reproducible across different sites with diverse cohorts. These findings support the utility of CVR as a biomarker in future clinical trials of SVD and VCID. In a recent study, we also evaluated CVR and its association with cognition in a group of aging people with HIV (PWH) and compared the results to non-HIV elderly controls with both normal cognition and VCID. Our results show that CVR in PWHs with HIV-associated Neurocognitive Disorders (HAND) was significantly lower than that of non-HIV controls with normal cognition, but similar to that of non-HIV controls with VCID. Among PWHs with HAND, lower CVR was associated with worse MoCA score. Therefore, CVR may also be a useful biomarker to assess vascular function in PWH and HAND.

Supported by NINDS/R01NS115771, 3R01NS115771- 03S1, UH2/3 NS100588.

##### Intrathecal Drug Delivery: Imaging Insights for Neuroimmune Pharmacology.

Verma, Ajay, MD, PhD; General Partner, Formation Venture Engineering, Beverly, MA 01915^2^CEO, Twilight Bioscience, Beverly, MA 01915.

Background: Intrathecal (ITH) drug delivery bypasses the blood-brain barrier, yet spatial and cellular pharmacokinetics remain poorly understood. Characterizing ITH therapeutics distribution is critical for targeting CNS immune reservoirs in HIV, neuroinflammation, and neurodegenerative diseases. Methods: We employed multi-modal translational neuroimaging (SPECT/PET-CT, MRI, fluorescence tomography) in rodents, non-human primates, and humans to characterize ITH-administered labeled probes: small molecules, antisense oligonucleotides, proteins, and nanoparticles. Studies examined neuraxial spread, CNS penetration, cellular uptake, and peripheral clearance over hours to weeks. Results: Small molecules exhibited rapid convective spread, CSF–interstitial fluid exchange, and peripheral clearance with minimal lymphatic retention. Larger molecules (≥8 kDa) showed prolonged retention in meningeal and perivascular compartments, uptake by CD206+ macrophages, and trafficking to cervical lymph nodes. Imaging revealed a CNS-wide reticuloendothelial system engaged by ITH macromolecules, with persistent signal in meningeal lymphatics and perivascular spaces. CNS penetration occurred via per-vascular and trans-pial routes, while substantial drug remained sequestered in immune-rich meningeal niches. Conclusions: ITH delivery of large therapeutics preferentially engages meningeal lymphatics and macrophages, which are both pharmacokinetic barriers and targets. For neuroimmune pharmacology, these findings inform strategies to target CNS myeloid reservoirs and modulate inflammatory responses.

Supported by BioPharma.

##### Immune Transformation of Humanized CD4/CCR5/C1qbp/MHC Mice Facilitates Productive HIV-1 Infection

Zhang, Chen, PhD^1^, Du , XQ, MS^1^, Yeapuri, P, PhD^1^, Dash, PK, PhD^1^, Sillman, BJ, PhD^1^, Mosley, RL, PhD^1^, Edagwa, B, PhD^1^, Lloyd, K.C, PhD^2^, Gendelman, HE, MD^1^; ^1^Department of Pharmacology and Experimental Neuroscience, University of Nebraska Medical Center, Omaha, NE, 68198 United States. ^2^UC Davis Mouse Biology Program, UC Davis Medical Center, Sacramento, CA, 95817 United States.

Rodents cannot be infected with HIV as their CD4, CCR5, and CXCR4 are unique from the human proteins; rodent post-transcriptional restriction factors inhibit viral RNA export, translation, assembly, and release; and robust murine NK and CD8+ T cells clear infected cells. Transgenic mice demonstrate random transgene insertion arrest virion production. Here, we report a novel CRISPR-Cas9 knock-in (KI) mouse that sustains HIV infection through targeted insertion of humanized CD4, CCR5, and C1qbp[D106G] at their respective loci. All site-specific CRISPR KIs were confirmed using RT-qPCR and Sanger Sequencing. Genotypic and flow cytometry tests demonstrated CD4+ T-cells isolated from peripheral blood, spleen, thymocytes, and lymph nodes. Each of the cells expressed huCD4/CCR5 in their correct orientation. Ex vivo HIV-1_ADA_ (MOI = 0.1) challenge in rodent PBLs led to productive HIV-1 replication. Infectivity was affirmed after intraperitoneal HIV-1ADA (105 TCID50) challenge and infection demonstrated by plasma viral load and DNA (qPCR/ddPCR). Stained splenocytes from infected animals and formalin-fixed spleen tissues affirmed HIV-1p24 antigen expression. CD8+ T-cell depletion accelerated viral infection. This CRISPR KI mouse model enables physiologically regulated expression of HIV entry receptors and represents an advance over transgenic systems, providing a critical platform for therapeutic and vaccine research.

#### Plenary Keynote Lecture

##### Neuroscience Research Priorities for NIDA’s HIV Research Program

Volkow, N, MD ^1^; ^1^National Institute on Drug Abuse, National Institutes of Health, Bethesda, MD 20852.

Neuroscience research is central to advancing NIDA’s HIV portfolio by elucidating mechanisms at the intersection of HIV infection, substance use disorders (SUD), and brain health, while enabling data-driven strategies to improve clinical and public health outcomes. Priority areas focus on HIV–SUD comorbidity across cellular, circuit, and systems levels, including how addictive substances, antiretroviral therapy (ART), and medications for SUD interact to shape neuroimmune signaling, neuroinflammation, CNS viral persistence, and neurocognitive dysfunction. Emerging evidence highlights mitochondrial mechanisms as critical mediators of neuropathology and accelerated aging in people living with HIV (PLWH) and SUD. Accordingly, studies investigate how HIV, ART, and substances disrupt mitochondrial function, stress responses, and metabolic signaling in neural and immune cells, with the goal of identifying targetable pathways to restore cellular resilience. NIDA’s portfolio includes cohort studies of PLWH and key populations at risk for HIV, enabling longitudinal analyses of neurocognition, SUD, HIV, and comorbid infectious and psychiatric conditions. Single-cell and single-nucleus approaches enable high-resolution mapping of HIV and substance effects across brain cell types and transcriptional networks, which, when integrated with functional validation, link molecular alterations to circuit dysfunction and behavior. NIDA also prioritizes computational modeling, biomarker discovery, and translational strategies to improve CNS drug delivery. Together, these neuroscience-driven efforts aim to generate mechanistic insight and scalable interventions to reduce the burden of HIV–SUD comorbidity.

### Symposium 3 - Early Career Investigator Travel Awardee (ECITA) Symposium

#### 1. Dopaminergic Modulation of Viral Replication in HIV-Infected IPSC-Derived Organoids Exposed to Stimulant Drugs

Agarwal, Y, BS^1^, Ogbolu, V, MS^2^, Montilla, J, MS^1^, Kist, T, BS^1^, Daniali, M, BS^1^, Curley, E, BS^1^, Modica, A, BS^1^, Mohan, N, PhD^2^, Wig, P, BS^1^, Ramakrishnan, S, MS^2^, Emanuel, K, BS^3^, Niu, M, PhD^3^, Fox, HS, MD, PhD^3^, Matt, SM, PhD^1^, Qiang, L, MD, PhD^2^, Gaskill, PJ, PhD^1^; ^1^Department of Pharmacology & Physiology, Drexel University College of Medicine , Philadelphia, PA, 19102 United States. ^2^Department of Neurobiology & Anatomy, Drexel University College of Medicine, Philadelphia, PA, 19129 United States. ^3^Department of Neurological Sciences, University of Nebraska Medical Center, Omaha, NE, 68198 United States.

Nearly half of people with HIV experience neurologic symptoms due to persistent viral reservoirs in microglia and macrophages that mediate chronic neuroinflammation and synaptodendritic damage. This may be accelerated by stimulant use disorder (methamphetamine [METH] or cocaine [COC]), a prevalent condition associated with higher neuroinflammation and viral load. Our lab has shown that dopamine (DA), a neurotransmitter elevated by stimulants, increases cytokine release and HIV replication in myeloid cells in vitro, suggesting a mechanism by which stimulant use worsens neuroHIV. To test this, we developed human iPSC-derived cortical and midbrain organoids integrated with microglia to model low (cortical) and high (midbrain) dopaminergic environments. Organoid identity and microglia integration were validated using immunostaining, gene expression, and single-cell RNA sequencing. METH-induced DA release in midbrain organoids was assessed using a live-cell assay, and multi-electrode arrays revealed METH-induced neuronal activity in midbrain cultures. Organoids were exposed to HIV +/- METH or COC. HIV replication and cytokine secretion were measured in supernatants by AlphaLISA. METH and COC increased viral secretion in midbrain organoids but not cortical organoids, suggesting that stimulant-induced dopamine release enhances viral replication. CXCL10 release, correlated with p24 levels, is derived from activated astrocytes. These findings highlight the role of regional neurotransmitter dynamics in shaping neuroimmune responses to HIV and neuropsychiatric comorbidities.

Supported by NIDA (R33DA058501)

#### 2. Hydrogen Sulfide Rescues Microglia from HIV Tat-driven Ferroptosis: Implications for HIV-Associated Neuroinflammation

Ahsan, A Ul, PhD^1^, MartÃnez-Cuevas, F L, PhD^1^, Horanieh, E, MD^1^, Singh, S, PhD^1^, Deshetty, U M, PhD^1^, Periyasamy, P, PhD^1^, Buch, S, PhD^1^;

^1^Experimental Neuroscience and Pharmacology, University of Nebraska Medical Center, Omaha, NE, 68105 United States.

The HIV transactivator of transcription (Tat) protein drives neuroinflammation and neurodegeneration in NeuroHIV, largely by inducing oxidative stress. Ferroptosis, a form of iron-dependent, regulated cell death marked by lipid peroxidation, has emerged as key feature of HIV-associated neurocognitive disorders (HAND) pathogenesis. This study investigated the neuroprotective potential of hydrogen sulfide (H2S), an endogenous gasotransmitter known for its antioxidant properties, against HIV Tat-induced ferroptosis in microglial cells. Methods: BV2 microglia were pretreated with the H2S donor NaHS (100 ÂµM) before HIV Tat (100 ng/mL, 48 h) exposure. Ferroptotic hallmarks (iron, ROS, lipid peroxidation) and key markers were assessed via imaging and Western blotting. Results: NaHS significantly mitigated HIV Tat-induced cytosolic Fe2+, (ROS) production, and membrane lipid oxidation, effectively restoring redox equilibrium. Mechanistically, NaHS suppressed pro-ferroptotic mediators, including acyl-CoA synthetase long-chain family member 4(ACSL4) and 4-hydroxynonenal (4HNE, while concomitantly upregulating the antioxidant enzymes solute carrier family 7-member 11(xCT) and glutathione peroxidase 4(GPX4). Furthermore, NaHS reduced Tat-induced secretion of proinflammatory cytokines IL1β^2^, IL6 and TNFα, and reduced LDH release. Conclusion: These findings establish NaHS is a potent anti-ferroptotic agent in HIV Tat-challenged microglia. By modulating the SLC7A11-GPX4 antioxidant axis in Tat mediated ferroptosis, H2S donors could be promising adjunctive therapies in NeuroHIV.

Supported by Startup funds from Prof. Shilpa Buch

#### 3. Development of A New Approach Method (NAM) to Examine BBB Integrity and ABC Efflux Transporter Activity and Expression in The Context of HIV Antiretroviral Therapy

Al Shakarchi, Z, MS^1^; ^1^Department of Pathology, Immunology, & Laboratory Medicine, College of Medicine, University of Florida, Gainesville, FL, 32611 United States.

The blood-brain barrier (BBB) not only restricts the entry of blood solutes and xenobiotics into the CNS but also plays a critical role in clearing metabolic waste from the brain parenchyma. This clearance depends on the activity of ATP-binding cassette (ABC) efflux transporters (i.e., P-gp/ABCB1, and BCRP/ABCG2). Transporter function at the brain endothelium can be altered by various pathological and pharmacological conditions. In the context of HIV, little is known about how efflux transporter function from brain to blood is impacted by ART. Some ART drugs may adversely alter the clearance/accumulation of harmful metabolites resulting from neuronal activity. To investigate how ART could affect clearance transport, we have developed a novel approach methodology and assay to measure waste elimination via efflux transporter activity. Using DLP bioprinting, to create scaffolds containing a hollow vessel and a central compartment. Endothelialization of the vessel lumen enables BBB modeling and functional assessment of efflux-mediated waste clearance under physiologically relevant conditions. Once printed and cellularized, the central chamber is filled with a fluorescent targeted by efflux transporters (such as R123 for Pgp), and ART drugs that are introduced into the vascular compartment. The tracer accumulates as a function of time in the lumen of the vascular compartment reflecting clearance rate. This novel assay provides a platform to study ART and BBB integrity /ABC transporter function, advancing our understanding of their implications for CNS health in PLWH.

Supported by R01DA052970, R01DA058536

#### 4. HIV-1 Drives Alzheimer’s Disease Pathologies in APP Knock-in Mice

Bhattarai, S, MS^1^, Yeapuri, P, PhD^1^, Foster, EG, BS^1^, Kadry, R, MS^1^, Lu, Y^1^, Sapkota, R^1^, Kumar, M^1^, Mitra, A, PhD^2^, Pathak, H, PhD^2^, Gorantla, S, PhD^1^, Poluektova, LY, MD, PhD^1^, Mosley, RL, PhD^1^, Gendelman, HE, MD^1^;

^1^Pharmacology and Experimental Neuroscience, University of Nebraska Medical Center, Omaha, NE, 68198 United States. ^2^Pathology and Laboratory Medicine, University of Kansas Medical Center, Kansas City, KS, 66160 United States.

The prevalence of Alzheimer-like pathology is rising among people living with HIV-1, yet the mechanistic interplay between chronic viral infection and amyloid pathology remains poorly understood. A major barrier to this has been lack of suitable animal model that permits the simultaneous study of HIV neuropathogenesis and Alzheimer’s disease (AD). We developed a novel humanized AD model NOG/APPKM670,671NL/IL-34(NAIL), which supports productive HIV-1 infection and human-like microglia development. Four-month-old NAIL mice were infected with the HIV-1ADA strain, and tissue samples were collected eight weeks post-infection. We found HIV-1 infection significantly increases the amyloid (Aβ42) deposition and induces distinct transcriptional changes across neurons, astrocytes, and microglia. Spatial transcriptomics revealed largely independent Aβ and HIV-1 responses, but their combination (Aβ+HIV-1) elicited a strong synergistic transcriptional response. In neurons, TRAF2, TRAF6, ITIM3 and TRIM26 were upregulated, indicating inflammatory activation while SNAP25, STX1A, STXBP1, SYN1, DLG4 and GRINA2A were downregulated reflecting synaptic dysfunction. Astrocytes displayed increased expression of GFAP and VIM, consistent with reactive astrogliosis, and microglia showed elevated BACE1 and RTN3, with reduced LRP1 and LAMP1, indicating impaired amyloid clearance. Overall, HIV-1 infection in Aβ-rich regions drives neuroinflammation, synaptic loss, and accelerated AD-like pathology, positioning the humanized NAIL model as a unique platform to study HIV-AD comorbidity mechanisms.

Supported by NIH P01 DA028555, R01NS034239-30, RO1 AG043540, 5T32NS105594-08

#### 5. Development of The First Microphysiological System for The Study of The Blood-Cerebrospinal Fluid Barrier Interface

Birru, B, PhD, Ramirez, S H R, PhD, Andrews, A M A, PhD;

Department of Pathology, Immunology and Laboratory Medicine, University of Florida, Gainesville, FL, 32610 United States.

Although the blood-brain barrier (BBB) has received extensive attention, the BCSFB remains comparatively understudied, despite its critical role in protecting the brain from neurotoxins, pathogens, and inflammatory mediators. Dysfunction of the BCSFB has been implicated in Alzheimer’s disease, multiple sclerosis, meningitis, and a variety of neuroinflammatory and neurodegenerative states. The development of a microphysiological system or a novel approach methodology for the BCSFB is needed to study neuropathological changes and for therapeutic drug discovery. To address this gap, we engineered a custom 3D bioprinted scaffold with co-cultures of primary human choroid plexus epithelial (CPE) cells and brain microvascular endothelial cells (BMECs) coupled with a microfluidic perfusion system that mimics physiological shear stress. The scaffold architecture contains a hollow vessel structure, enabling BMECs to line the lumen while the CPE cells orient along the surface of a central compartment, recapitulating the BCSFB. Pilot optical imaging confirms successful endothelial seeding, lumen integrity, and a continuous epithelial monolayer. We further conducted barrier permeability studies to evaluate the barrier integrity of this BCSFB model. This study presents a proof-of-concept approach that demonstrates the feasibility of generating a microfluidic, 3D bioprinted construct capable of recapitulating the structural features of the human BCSFB.

Supported by Experimental Pathology Innovative grant, Department of Pathology, Immunology and Laboratory Medicine, UF.

#### 6. Chromatin Disruption and DNA Damage in HIV-Tat+ Opioid Exposure: Protective Role of Dimethyl Fumarate

Chennakesavan, K, PhD^1^, Samikkannu, M, MS^1^, Karnati, A, BS^1^, Hammond, H R, MS^2^, Eans, SO., MS^2^, Kaufman, M J, PhD^3^, McLaughlin, J P, PhD^2^, Thangavel, S, PhD^1^;

^1^Department of Pharmaceutical Science, Irma Lerma Rangel College of Pharmacy, Texas A&M University, College Station, TX, 77801 United States. ^2^Department of Pharmacodynamics, College of Pharmacy, University of Florida, Gainesville, FL, 32610 United States. ^3^McLean Imaging Center, McLean Hospital/Harvard Medical School, Belmont, MA, 02478 United States.

DNA damage, particularly double-strand breaks (DSBs), threatens genomic stability and accelerates neurodegeneration when unrepaired. HIV infection and opioid misuse are major contributors to HIV-associated neurocognitive disorders (HAND); however, the mechanisms by which they impair genomic integrity and neuroimmune balance remain unclear. This study investigates the effects of dimethyl fumarate (DMF) on DNA repair proteins (DRPs) and KAT5-mediated acetylation in the frontal cortex of HIV-iTat mice with or without morphine. iTat mice received DOX (100 mg/kg) to induce Tat expression, escalating doses of morphine (10-75 mg/kg for 4 days), and/or DMF (30 mg/kg, p.o., for 7 days). Behavioral testing (NOR), immunostaining, and molecular analysis were used to evaluate DRPs, ATM signaling, KAT5, cGAS, and inflammatory markers. iTat and Mor synergistically disrupted LOCK domains, decreased H3K9me3, and increased H3K9Ac, thereby impairing ATM activation and γH2AX signaling, which led to DSB accumulation in microglia. ERCC-2, -3, and -6 were selectively downregulated in iTat +Mor mice, indicating compromised repair fidelity. HIV-induced DNA damage activated cGAS-STING innate immune pathways. DMF restored H3K9me3, stabilized LOCK domains, reduced ATM kinase and NBS1, and suppressed KAT5-cGAS-STING and AIM2/NLRP3 signaling, while preventing behavioral deficits. Overall, these findings demonstrate that HIV-Tat and morphine synergistically impair chromatin-mediated DNA damage responses, whereas DMF mitigates genomic instability and represents a promising therapeutic candidate for HAND.

Supported by R01- DA055568 from NIH/NIDA

#### 7. Modeling HIV-Associated Neuroinflammaging: A Microphysiological Approach to Blood Brain Barrier Dysfunction

Das, RK, PhD^1^, Simlote, N, BS^1^, Aalinkeel, R, PhD^1^, Prasad, PN, PhD^2^, Schwartz, SA, MD, PhD^1^, Mahajan, SD, PhD^1^;

^1^Department of Medicine, The State University of New York at Buffalo, Buffalo, NY, 14203 United States. ^2^Institute for Lasers, Photonics and Bio-photonics, The State University of New York at Buffalo, Buffalo, NY, 14260 United States.

HIV proteins Tat and gp120 accelerate aging and neurodegeneration by promoting cellular senescence, neuroinflammaging, blood-brain barrier (BBB) disruption, and lipid metabolism dysfunction in individuals on antiretroviral therapy (ART). To model these processes in-vitro, we used a microphysiological system (MPS) that enables controlled induction of BBB aging with neuroinflammaging conditions (5 sets); control group; HIV group (HIV-1 proteins); cytokine-induced aging group treated with TNF-α, IL-1β, IFN-γ followed by H_2_O_2_ to mimic chronic neuroinflammaging; combined HIV+cytokines induced aging group to replicate the synergistic effects of viral persistence and neuroinflammaging and ART-treated group exposed to HIV+cytokines+ART (combined tenofovir, emtricitabine, dolutegravir) to assess the extent of cure in neuroinflammaging damage. Our 2D BBB model incorporates a tri-culture of human BBB cell lines, grown in a 2-compartment system separated by a 3 μm polyethylene terephthalate membrane. BBB integrity was assessed via trans-endothelial electrical resistance (TEER) and FITC-dextran permeability. Our results show that aging and HIV viral protein promotes BBB permeability by ∼35%, lowers tight junction (Claudin-5, Occludin, ZO-1) protein expression, increases senescence markers (SA-β-gal), ROS (MitoSOX), lipid peroxidation, dysregulation and glycocalyx degradation. Further, ART marginally mitigated the effects, and our MPS-BBB model effectively recapitulates the aging in human BBB model supporting studies related to human age-related neurovascular dysfunction.

Supported by 1R21AG083727-01A1

#### 8. Impact of Social Stress on Microglia-Neuron Interactions in the Nucleus Accumbens

Franco, DF, Siemsen, BS, PhD, Kumar, GK, PhD, Key, SLK, Hingmire, MH, Das, PD, Befano, KB^1^, Fox, MF, PhD, Lobo, MKL, PhD; Department of Neurobiology, University of Maryland, Baltimore, MD, 21201 United States. ^2^Department of Anesthesiology and Perioperative Medicine, Pennsylvania State University College of Medicine, Hershey, PA, 17033 United States.

Stress is a risk factor for neuropsychiatric disorders and alters neuron and myeloid structure in the nucleus accumbens (NAc), a hub for reward and motivation. Chronic stress increases peripheral cytokines in individuals with depression and disrupts CNS myeloid-neuronal communication in rodents exposed to social stress. Prior work has shown that chronic social defeat stress (CSDS) yields dendritic atrophy in NAc dopamine receptor-1 expressing medium spiny neuron (D1-MSNs) in mice displaying negative affective behavior. Since microglia facilitate neuronal dendritic adaptations after social stress, we characterized microglia and D1-MSN interactions in the NAc after CSDS. While we observed a cell-subtype reduction in microglia-D1-MSN contact in the NAc after CSDS, preliminary evidence shows that mice exposed to Chronic Witness Defeat Stress (CWDS) show an increase in microglia-D1-MSN contact in the NAc. Thus, CSDS and CWDS may alter microglia-D1-MSN contacts in opposing ways. To understand the NAc molecular landscape during social stress, we customized a Nanostring panel of neuroimmune genes. While 10 days of CSDS and CWDS yields the greatest number of differentially expressed genes, they do not overlap among CSDS males and CWDS male and female mice. These results highlight a specific time point of interest for neuron-microglia crosstalk and underscores sex-specific neuroimmune interactions. Identifying microglia mechanisms contributing to altered neuronal dendritic morphology and negative affective behaviors can identify novel therapeutic targets for stress-related disorders.

Supported by NIMH

#### 9. Behavioral Studies and In-Vivo Brain MR Spectroscopy in Human Microglia NOG-hIL34 Mice

Kamdi, S, PhD^1^, Heredia, A, PhD^1^, Ernst , T, PhD^1^, Khorshidi, F, MD^1^, Hrdlick, A, BS^1^, Cunningham, E, BS^1^, Shichman, L, MS^1^, Bernard, S, BS^1^, Walczak, P, MD, PhD^1^, Lobo, M K, MD, PhD^2^, Gorantla, S, PhD^3^, Chang, L, MD^1^; ^1^Department of Diagnostic Radiology and Nuclear Medicine, ^2^Department of Neurobiology, University of Maryland, Baltimore, MD, 21227 United States. ^3^Department of Pharmacology and Experimental Neuroscience, Center for Neurodegenerative Diseases, University of Nebraska Medical Center, Omaha, NE, 68198 United States.

Introduction: NOG (NOD/Shi-scid/IL-2RÎ^3^^null^) mice carrying human IL-34 (NOG-IL34) support reconstitution of human microglia after transplantation with CD34+ human hematopoietic stem cells (HSC-NOG-IL34) mice and are used to model human neuroimmune function. This study evaluated behavioral, immunological, and neurochemical profiles in NOG-IL34 mice. Methods: NOG wild-type (WT) mice were crossed with NOG-IL34 transgenic mice to generate WT (NOG) and heterozygous (HET; NOG-IL34) offsprings. Neonates were sub-lethally irradiated, engrafted with human CD34+ stem cells, and genotyped. At 12 -20 weeks, behavioral testing was conducted in 78 NOG-IL34 mice (38 males, 40 female) and 59 NOG mice (28 males, 31 females) using open field, novel object recognition, social interaction, rotarod, and splash test. In-vivo MR spectroscopy measured N-acetyl-aspartate, myo-inositol, choline compounds, total creatine, and glutathione. Results: Compared with NOG mice, NOG-IL34 mice showed less social interaction, more anxiety-like behavior, less exploratory drive, longer grooming latency, and sensory or motor deficits (p < 0.05). Females showed higher human CD45+ T-cell levels than males, and NOG-IL34 mice showed an age-related rise in CD45+ T cells, with high engraftment efficiency. No significant genotype, sex, or age effects were observed in MR spectroscopy. Conclusion: NOG-IL34 mice show distinct behavioral and immune changes without significant alterations in brain metabolites, supporting a role for human microglia in modulating behavior and their use in neuroimmune and behavioral research.

Supported by NIDA-DP1DA53719

#### 10. OPN as a Neuroimmune Link Between CNS Inflammation and Peripheral Bone Pathology in HIV Infection

Kumar, S, PhD^1^, Davis, E, PhD^1^, Louie, P, MS^1^, Loganathan, S, PhD^1^, Shirmamedova, A, BS^1^, Mehta, A, MS^1^, Brown, A, PhD^1^;

^1^Department of Neurology, Johns Hopkins School of Medicine, Baltimore, MD, 212187 United States.

HIV infection is linked to persistent neuroimmune activation and accelerated bone loss, yet the mechanisms connecting central nervous system (CNS) inflammation to peripheral skeletal deterioration remain unclear. Using HIV-infected humanized NSG (hu-NSG) mice, we investigated the role of Osteopontin (OPN/SPP1), a cytokine-like extracellular matrix protein with neuroimmunomodulatory properties. HIV infection induced sustained elevation of OPN in both CNS and skeletal tissues, confirmed by immunofluorescence. To reflect clinically relevant conditions, a subset of HIV-infected mice received continuous triple-drug antiretroviral therapy (ART) via medicated chow, and the impact of ART on skeletal outcomes is currently under investigation. Elevated OPN levels were associated with disrupted bone homeostasis, marked by enhanced osteoclastogenesis (increased RANK expression and TRAP-positive osteoclasts) and suppression of osteoblastic markers osteocalcin (OCN) and Osterix (OSX). MicroCT analysis revealed trends toward reduced bone volume fraction and trabecular parameters, consistent with progressive bone loss. Importantly, pharmacological inhibition of OPN using specific aptamers reduced osteoclastic activity and partially rescued the bone phenotype, demonstrating a functional role for OPN in HIV-associated skeletal degeneration. Together, these findings identify OPN as a key neuroimmune mediator linking HIV-driven CNS inflammation to peripheral bone pathology and highlight OPN-targeted strategies to mitigate inflammation-associated skeletal comorbidities in chronic HIV infection.

Supported by NIH

#### 11. Tunneling Nanotubes Mediate the Intercellular Spread Of HIV-Gag-Vrna in Glial Cells through A Myosin-X-Dependent Mechanism

Ramirez, JR, BS^1^, Valdebenito-Silva, SV, PhD^1^; ^1^Department of Neuroscience, The University of Texas Medical Branch, Galveston, TX, 77555 United States.

HIV affects 38.1 million people globally and remains a pandemic. Traditional therapeutic approaches have limited effectiveness against myeloid glial viral reservoirs, which significantly contribute to HIV-associated neurocognitive disorders (HAND), even with antiretroviral therapy (ART); thus, new molecular targets are greatly needed. Classical models explaining HIV spreading, such as the cell-free virus and virological synapse, fail to explain the rapid viral spread under conditions of low extracellular viral levels. We proposed a new model for HIV spread through Tunneling Nanotubes (TNTs), an F-actin-rich cell communication bridge. Our research has demonstrated that TNTs play a role in spreading HIV infection, showing the presence of HIV components in TNTs through proteomics data. Here, we show that HIV-Gag-vRNA, a complex protein with genomic RNA, is co-localized with Myosin-X, a motor protein that facilitates TNT transfer. Using immunofluorescent live-cell imaging, we observed HIV-Gag-vRNA complex is transferred via TNTs. Our findings indicate that HIV-infected cells can transmit viral components to non-infected cells via Myosin-X-mediated TNT transfer. These findings present a novel infection model, Myosin-X-mediated TNT transfer as a facilitator for the movement of HIV proteins. Our results showed biophysical characteristics of TNTs, including their formation time, transfer duration, and molecule release timing. This research could identify potential targets for new therapies targeting HAND.

Supported by NS137812, MH128082 and MH134761

#### 12. Combined EcoHIV and Methamphetamine Exposure Dysregulates Neuroimmune Responses Which Drives Cognitive and Neuropsychiatric Dysfunction

Schindler, KA, BS^1^, Park, M, PhD^2^, Baines, K, BS^2^, Pieklo, G, BS^2^, Torices, S, PhD^2^, Beurel, E, PhD^3^, Toborek, M, MD, PhD^2^; ^1^Medical Scientist Training Program, University of Miami, Miller School of Medicine, Miami, FL, 33136 United States. ^2^Department of Biochemistry and Molecular Biology, University of Miami, Miller School of Medicine, Miami, FL, 33136 United States. ^3^Department of Psychiatry and Behavioral Sciences, University of Miami, Miller School of Medicine, Miami, FL, 33136 United States.

People living with HIV (PLWH) experience a higher risk of cognitive dysfunction and depression despite modern antiretroviral therapy. Cognitive impairment is linked to atrophy of dopaminergic brain regions, and HIV proteins may interact directly with dopamine transporters and increase neurotoxicity. Methamphetamine (METH) misuse is associated with an increased risk of contracting HIV as well as higher viral loads in PLWH. Because METH increases dopamine signaling and neuroinflammation, and HIV induced neurocognitive impairment is linked to similar mechanisms, the need to explore their interaction is high. This study aims to investigate the hypothesis that the combination of METH exposure and ecoHIV infection leads to increased neuroinflammation, driven by dopamine dysregulation, and subsequent cognition and mood changes. Our data indicate that cognition and depression are modulated by HIV infection and short-term METH administration in mice in a sex specific manner, without synergism between the treatments. We also demonstrate increased neuroinflammation (elevated brain IL-6, NLRP3 expression) in ecoHIV infection alone, while combined exposure to METH+ecoHIV results in NLRP3-dependent immune suppression and an increase in anti-apoptotic machinery, altogether suggesting that the interaction may enhance HIV infectivity. Further, our data indicate that ecoHIV infection and METH use can dysregulate dopamine receptors and dopamine breakdown machinery. This demonstrates that dopamine modulation offers a future treatment avenue for METH/HIV induced neuropsychiatric dysfunction.

Supported by NIH awards DA059849, DA050528, MH128022, HL126559, DA060085, and NS14170

### Drs. Mahendra and Adarsh Kumar Memorial Lecture

#### Neuroimaging Insights into Substance Use: A Focus on Nicotine Dependence.

Janes, AC, PhD; Deputy Chief, Neuroimaging Research Branch, NIDA Intramural Research Program, Baltimore, MD 21224.

Nicotine dependence remains a leading cause of preventable mortality, despite declines in smoking rates and available cessation treatments. This persistence reflects heterogeneity in mechanisms driving nicotine use and relapse, including individual sensitivity to nicotine, brain responses to smoking cues, and psychiatric comorbidity. This talk first examines brain systems engaged during smoking cue reactivity, including the salience network (SN) and default mode network (DMN), highlighting how individual variability in brain responses contributes to relapse vulnerability. We then use resting-state fMRI to characterize these networks, focusing on intrinsic organization and dynamic activity patterns. Specifically, interactions between the SN and DMN are associated with clinically relevant measures. Using temporal dynamic metrics, we show that, relative to non-smokers, individuals with nicotine dependence spend more time in a mixed SN–DMN state. Further, incorporating transdiagnostic clinical measures improves characterization of individual neurobiological profiles relevant to nicotine use. Importantly, dynamic properties are sensitive to pharmacological modulation, supporting their relevance as a potential target for treatment. Nicotine also produces differential effects in individuals with versus without mental health conditions. Together, this work highlights how individual differences in brain function, clinical presentation, and psychiatric comorbidity shape addiction vulnerability and inform treatment-relevant neurobiology.

Supported by NIDA-Intramural Research Program.

### Symposium 4 - Implications of RNA Regulation in Neuroimmunological Responses

**Chair:** Shiden Solomon, PhD, University of Pennsylvania, Philadelphia,

#### Pataming Alu in The Primate Brain: Ku Suppresses RNA-Mediated Innate Immunity and Reshapes the Transcriptome.

Zha, SZ, MD, PhD, Zhang, C, PhD, Zhu, T, PhD, Yu, T, BS, Yoon, J, PhD, Lee, B, BS; Institute for Cancer Genetics, Columbia University, New York, NY 10032-3802.

The massive Alu expansion in higher primates enables novel neuronal subtypes and function, but also generates abundant dsRNA, which can activate innate immune sensors and disrupted RNA processing. Ku70 and Ku80 form the Ku heterodimer, best known for initiating non-homologous end joining (NHEJ) at DNA double-strand breaks. Although Ku can bind dsRNA, its RNA-dependent physiological role remained unclear. Ku is dispensable for murine development but essential in human cells, and its expression increases sharply ∼ 100-fold during primate evolution in parallel with Alu expansion. Here, we show that Ku acts as a primate-specific RNA regulator that suppresses RNA-mediated innate immunity and shapes the human transcriptome. Ku depletion in human cells, unlike loss of core NHEJ factors, triggers interferon and NF-κB signaling through the dsRNA sensors MDA5 and RIG-I and the adaptor MAVS, followed by activation of PKR and the OAS/RNase L pathway, leading to translational arrest and growth inhibition. Ku directly binds dsRNA stem-loops enriched in primate-specific antisense Alu elements, limiting aberrant immune activation. In parallel, Ku suppresses Alu-associated alternative splicing independently of IFN and NHEJ, affecting ∼8–10% of splicing events. Strikingly, Ku expression is lower in brain, correlating with more permissive Alu-derived splice variants critical for neuronal function. Together, these findings identify Ku as a key evolutionary adaptation that tames Alu expansion by coordinating innate immune suppression and transcriptome remodeling critical for human brain function.

Supported by NIH and National Cancer Institute.

#### Effects of TDP-43 Dysregulation on Astrocytes and Viral Infection

Orr, AG, PhD^1^; ^1^Helen and Robert Appel Alzheimer’s Disease Research Institute, Brain and Mind Research Institute, Weill Cornell Medicine, New York, NY, 10021 United States.

TDP-43 is a nuclear DNA/RNA-binding protein, and its dysregulation is a key hallmark of FTD, ALS, and other neurodegenerative disorders. TDP-43 pathology impairs brain function, but exact effects and therapeutic strategies are not clear. In disease, TDP-43 can accumulate in the cytoplasm and affect stress granule formation and interferon-linked signaling in neurons. Although astrocytes are implicated in neurodegenerative processes, how TDP-43 pathology affects astrocytes has not been defined. We found that astrocytic TDP-43 accumulation promotes aberrant chemokine signaling and induces astrocytic cell-stress pathways that alter nuclear pore proteins, nuclear membrane structure, and nucleocytoplasmic transport, and these effects are mediated by immune signaling mechanisms. Our results suggest that astrocytic stress-linked nucleocytoplasmic changes are a common feature of dementia and neuroinflammatory conditions and may occur in diverse pathologies. Our latest studies also reveal that mutant TDP-43 protects against infection by HSV-1, a prevalent neurotropic virus. This protective effect is conserved across diverse cell types and different mutant forms of TDP-43. We found that TDP-43 mutation disrupts host factors needed for viral binding and entry and prevents inflammatory responses to HSV-1 exposure, implicating TDP-43 in cell-intrinsic resistance to viral infection. Together, our findings suggest that TDP-43 pathology induces astrocytic stress-linked pathways and that TDP-43 is a crucial regulator of host-virus interactions.

Supported by the National Institutes of Health, BrightFocus Foundation, and the Alzheimer’s Association

#### Global Epitranscriptomic Alterations in HIV-Infected Monocyte-Derived Macrophages

Su, Yijing, Shi, X, MS, Wang, X, BS, Lou, J, MS, Li, P, MS, Jordan-Sciutto, K, PhD, Akay-Espinoza, C, MD; University of Pennsylvania, School of Dental Medicine, Philadelphia, PA 19104.

HIV-1 infection induces extensive transcriptional reprogramming in monocyte-derived macrophages (MDMs), yet its impact on the epitranscriptome—particularly native RNA modifications and poly(A) tail length—remains pool understood. RNA features such as poly(A) tail length and chemical base modifications, including N6-methyladenosine (m6A), 5-methylcytosine (m5C), and pseudouridine (Ψ), are key regulators of RNA stability, translation, and innate immune responses. Here, we use Oxford Nanopore direct RNA sequencing technology to profile global epitranscriptomic alterations in HIV infected MDMs. This amplification-free approach enables single-molecule analysis of native RNA, allowing direct measurement of poly(A) tail length and detection of RNA base modifications. Comparative analysis reveals widespread HIV-associated remodeling of poly(A) tail length distributions, with distinct classes of host transcripts exhibiting tail shortening or elongation following infection. Concurrently, we identify global and transcript-specific changes in m6A, m5C, and Ψ modification patterns affecting genes involved in antiviral defense, interferon signaling, RNA metabolism, and macrophage activation. Together, our analyses demonstrate coordinated reprogramming of poly(A) tail dynamics and RNA modifications during HIV infection in human MDMs and establish Nanopore direct RNA sequencing as a powerful platform for dissecting host–virus interactions at the epitranscriptomic level. Our study provides new insights into post-transcriptional mechanisms that may contribute to HIV persistence in macrophage.

Supported by NIDA.

#### Residual Viral RNA Replication within CNS Viral Reservoirs Drive Chronic Bystander Neuronal and Glial Damage

Eliseo E, PhD, Professor, Department of Neurobiology, John Sealy School of Medicine, William D. Willis, Jr., M.D., Ph.D. Professor (or Endowed Professor) in Neuroscience, The University of Texas Medical Branch (UTMB), Galveston, TX

As reported by UNAIDS in 2024, ∼40 million people worldwide live with HIV. Beyond immune compromise, HIV causes HIV-associated neurocognitive disorders (HAND). HIV infection of the CNS occurs within weeks of initial exposure when infected monocytes enter the brain and infect microglia, macrophages, and a small population of astrocytes. HIV associated chronic CNS damage has been linked to viral reservoirs, inflammation, and neurodegeneration, but the mechanisms of long-term toxicity remain unclear. Using human brain tissue from individuals with different ART durations, we found that myeloid cells (microglia/macrophages) are the main early viral reservoirs, with a smaller astrocyte population showing distinct decay dynamics. Although ART reduces reservoir size, most reservoirs retain low-level viral RNA (gag-pol) and express residual HIV proteins (p24, Tat, gp120, Nef). These proteins, even at fM–pM levels, induce lipid-mediated inflammation, metabolic dysfunction, and bystander neurotoxicity. Metabolic analyses of viral reservoirs revealed reliance on arachidonic-acid–driven inflammation. Also, we detected a critical weakness in the metabolism of these cells, relying on glutamate/glutamine to induce apoptosis. Targeting these vulnerabilities induced apoptosis in 90–95% of reservoir cells without reactivation. Our findings define the evolution of brain viral reservoirs, explain persistent CNS toxicity despite ART, and identify lipid-based inflammation and metabolic fragility as key therapeutic targets for neuroHIV and cure strategies.

### Symposium 5: Glial mechanisms of NeuroHIV

#### CSF1r Blockade During Virally Suppressed SIV Infection Decreases Neuroinflammation and Further Reduces SIV Reservoirs in The Brain

Woong-Ki Kim, PhD, Nguyen, HD, MD, PhD^1^, Siddiqui, S, PhD^1^, Ding, AK, MS^2^, Bodrog, S^2^, Zhao, M, PhD^3^, Li, Q, MD, PhD^3^, Williams, KC, PhD^2^

^1^Tulane National Biomedical Research Center, Tulane University, Covington, LA70433; ^2^Biology Department, Boston College, Chestnut Hill, MA 02467 ^3^HIV Cure and Viral Diseases Center, Wistar Institute, Philadelphia, PA 19104.

Perivascular macrophages (PVMs) are long-lived CSF1R-positive myeloid cells that serve as viral reservoirs and drivers of neuroinflammation during HIV/SIV infection. This study evaluated BLZ945, a brain-penetrant CSF1R inhibitor, during chronic SIVmac251 infection in rhesus macaques receiving antiretroviral therapy (ART). BLZ945 was administered either early or late during ART and outcomes were assessed before and after analytic treatment interruption. Both early and late BLZ945 treatment significantly reduced plasma and cerebrospinal fluid viral load compared with ART alone and lowered SIV DNA and RNA across multiple brain regions, with particularly strong effects in the occipital cortex. BLZ945 also reduced monocyte turnover and proliferation markers across all monocyte subsets, decreased brain CD163^+^ and CD68^+^ macrophages, and improved systemic inflammation as reflected by an increased lymphocyte-to-monocyte ratio. Transcriptomic analysis revealed a vascular-protective signature, including marked suppression of MEOX2 and other genes associated with endothelial dysfunction, leukocyte adhesion, and extracellular matrix remodeling. Pathway analyses indicated improved synaptic signaling and reduced immune activation, consistent with enhanced vascular stability and blood–brain barrier integrity. BLZ945 was well tolerated with no detectable toxicity. Overall, CSF1R inhibition with BLZ945 reduces CNS viral reservoirs, dampens neuroinflammation, and improves vascular homeostasis during chronic SIV infection, supporting its potential as a therapeutic strategy for HIV/SIV-associated neurological disorders.

Supported by NIMH & NINDS.

#### When Astrocytes Senesce: Functional Consequences for Brain Homeostasis in HIV and Methamphetamine Co-Morbidity

Lena Al-H, PhD; Dept of Microbial Pathogens and Immunity, Rush Medical College, Rush University, Chicago, IL, 60612 United States.

Despite effective ART, neuroHIV persists in part through chronic neuroinflammation and glial dysfunction, which may be exacerbated by methamphetamine (Meth) co-morbidity. We investigated how HIV and Meth converge on astrocyte pathways regulating CNS homeostasis. Using human iPSC-derived astrocytes, HIV infection and/or Meth exposure induced a senescence phenotype marked by increased p16INK4a with impairments across core astrocyte functions. Senescent astrocytes exhibited reduced clearance of apoptotic neurons linked to downregulation of the phagocytic receptor MEGF10, while restoration of MEGF10 rescued phagocytic capacity. HIV/Meth exposure shifted astrocyte secretomes toward proinflammatory profiles (IL-6, CCL2, CXCL1, ICAM-1), and astrocyte-conditioned media reduced neuronal markers (PSD95, NFL) and disrupted endothelial junction proteins, increasing monocyte transmigration in vitro. Mechanistically, pharmacologic activation or lentiviral expression of active Î^2^-catenin reversed senescence markers, restored MEGF10 expression, and rescued astrocyte phagocytic function. To extend these findings in vivo, we employed a humanized mouse model in which fluorescently labeled human astrocytes were xenotransplanted into NSG mice. In this chimeric system, HIV and/or Meth increased p16INK4a and reduced MEGF10 in engrafted human astrocytes, recapitulating in vitro phenotypes. Together, these data identify β-catenin suppression as a reversible regulator of astrocyte senescence and dysfunction in HIV and Meth exposure, underscoring their role in brain homeostasis.

Supported by R01DA057325

#### CCR5 Antagonists to Treat Mood and Cognitive Deficits in HIV-Infected Individuals

Conant, K, MD, Amontree, M, Alaiyed, S, Hummel, K, Greco, G; Neuroscience, Georgetown University Medical Center, Washington, DC 20007.

Despite combination antiretroviral therapy (cART), HIV+ individuals continue to experience some degree of cognitive dysfunction. In the post-cART era, chronic inflammation persists in the HIV+ brain and may contribute to disordered mood and cognition. The HIV-1 Tg26 transgenic mouse model is a well-characterized model of HIV associated neurological disorder of relevance to cART-controlled HIV-1-infected patients who lack active viral replication but suffer continuous stress from exposure to viral proteins. In addition, increased expression of pro-fibrotic molecules, including CCL5, is observed in Tg26 mice as well as humans with HIV or chronic inflammation. Herein we show that specific components of perineuronal nets (PNNs) are increased in Tg26 mice. PNNs are a specialized form of dense extracellular matrix that predominantly surround fast spiking GABA releasing inhibitory interneurons. PNNs can enhance the ability of these neurons to reduce excitatory neurotransmission and thus restrict neuroplasticity. In previous work we have shown that hippocampal PNN deposition is increased in a murine model of chronic stress, and that a therapeutic that reduces PNN levels normalizes anxiety and working memory deficits. We have also shown that CCR5 deficient mice have reduced PNN and TIMP-1 levels, as well as increased power of gamma oscillations which facilitate working memory. We will herein present background studies and new data to suggest that the safe FDA-approved CCR5 antagonist maraviroc be increasingly considered for HIV-infected patients with cognitive or mood disorders.

#### Glial Contribution to Pathogenesis in The Pain Neural Circuits Induced by NRTIs

Maurelli, M, MS^1^, Liu, X, PhD^2^, Tang, SJ, PhD^2^; ^1^Department of Pharmacology, Stony Brook University, Stony Brook, NY 11790 ^2^Department of Anesthesiology, Stony Brook University, Stony Brook, NY 11790.

To date, approximately 30.7 million people are receiving combination antiretroviral therapy (cART) to suppress human immunodeficiency virus (HIV) infection. First-line cART includes daily dosing of two separate nucleoside reverse transcriptase inhibitors (NRTIs). It has been shown that NRTIs induce mitochondrial perturbations resulting in leakage of reactive oxygen species (ROS) and the development of NRTI-induced pain in up to 60% of people living with HIV (PLWH). Due to the necessity of cART adherence, approximately 20 - 50% of PLWH are prescribed long-term opioids to manage NRTI-induced pain. It has been demonstrated that chronic NRTI and opioid use results in microglial activation and astrogliosis - a process involving the upregulation of neuronal Wnt5a secretion and activation of ROR2 on astrocytes. Upon ablation of astrocytes, knockdown of neuronal Wnt5a, knockdown of astrocytic ROR2, or antagonistic blocking of Wnt5a via Box5, NRTI and/or opioid-induced pain is, at least partially, alleviated. Our work has shown that microglial ablation, however, does not result in the same analgesic effect, indicating that microglia may be involved in other pathways contributing to pain. NRTIs and opioids have both been shown to increase ROS production within microglia. Our recent findings suggest that concurrent niacin administration decreases NRTI-induced oxidative stress within microglia, ultimately contributing to partial remediation of NRTI-induced pain. This work demonstrates a multifaceted approach in finding alternatives to opioids when treating HIV-associated pain.

Supported by R01DA057195 and R01DA062257.

## Tuesday, May 5^th^, 2026

### Symposium 6: Noncanonical roles of (endo)lysosomes in the central nervous system

**Co-Chairs:** Lindsay Festa, PhD, Assistant Professor, Children’s Hospital of Philadelphia, Philadelphia, PA

Yisel Cantres Rosario, PhD, Assistant Professor, University of Puerto Rico, San Juan, PR

#### TRPML1 Controls Lysosome Positioning to Shape Astrocyte Morphology

Maday, S, PhD; Department of Neuroscience, Perelman School of Medicine at the University of Pennsylvania, Philadelphia, PA 19104.

Lysosomes are critical for neuronal physiology and synaptic function, but their roles in astrocytes are poorly defined. Lysosome dysfunction can cause lysosomal storage diseases, including Mucolipidosis Type IV (MLIV), a neurodevelopmental and neurodegenerative disorder caused by loss-of-function mutations in MCOLN1, the gene encoding the lysosomal cation-permeable channel TRPML1. Recent cell-type-specific proteomics showed that TRPML1 knockout mice modeling MLIV exhibit the greatest protein dysregulation in astrocytes, particularly in cytoskeletal and postsynaptic pathways, suggesting astrocyte-specific vulnerability to lysosome dysfunction. Here, we define how TRPML1 influences lysosome trafficking and function in astrocytes using a neuron-astrocyte coculture system that recapitulates key aspects of astrocyte maturation and neuron-astrocyte interactions in vivo. Pharmacological activation of TRPML1 reduces lysosome motility in astrocyte branches, whereas loss of TRPML1 increases motility, identifying TRPML1 as a key regulator of lysosome dynamics in astrocytes. TRPML1-dependent lysosomal arrest requires polymerized actin which may position lysosomes at actin-enriched peripheral astrocyte processes (PAPs) that can contact synapses. In fact, modulating TRPML1 alters the phosphorylation of ERM proteins, actin-binding structural proteins enriched in PAPs, and regulates filopodial formation. These findings support a model in which TRPML1 helps dock lysosomes onto the actin cytoskeleton to influence astrocyte PAP structure and thereby modulate astrocyte morphology at synapses.

#### Roles of Exocytosis in Myelin Membrane Expansion and Plasticity

Lam, M, PhD^1^, Dehtiarov, O, BS^1^, Duan, L, BS^1^, Geraghty, AC, PhD^2^, Yalcin, B, PhD^2^, Monje, M, MD, PhD^2^, Zuchero, JB, PhD^1^; ^1^Department of Neurosurgery, Stanford University, Stanford, CA 94305 ^2^Department of Neurology and Neurological Sciences, Stanford University, Stanford, CA 94305.

Myelin accelerates conduction velocity along axons, and loss of myelin leads to cognitive deficits and physical disability. Cells that make myelin coordinate extreme feats of membrane trafficking to wrap axons and form a compact sheath. Despite its complex structure, myelin in the CNS changes in abundance and structure to adapt to new brain activity, requiring spatiotemporal coordination of membrane trafficking in myelin-forming oligodendrocytes. How does neuronal activity regulate membrane trafficking in oligodendrocytes? We previously discovered that exocytosis through the v-SNAREs VAMP2 and VAMP3 drives myelin membrane expansion during development. VAMP2/3 mediate membrane fusion at myelin sheath edges and at the innermost layer, where myelin interfaces with the axon. To determine if neuronal activity stimulates oligodendrocyte exocytosis, we co-cultured primary oligodendrocytes with active versus silenced neurons. Activity from glutamatergic neurons doubles the rate of VAMP3 exocytosis in oligodendrocytes in a calcium-dependent manner. To test how exocytosis sculpts myelin in vivo, we used optogenetic stimulation to induce activity-dependent myelination in the mouse corpus callosum while inhibiting oligodendrocyte exocytosis. We found that oligodendrocyte VAMP2/3 are required for activity-dependent sheath remodeling. Furthermore, motor learning and memory of a skilled forelimb reach task requires oligodendrocyte exocytosis. Thus, we uncover a cellular mechanism for spatiotemporal control of myelin addition that promotes neuroplasticity.

#### The Lysosome Is a Regulator of the Oligodendrocyte Cytoskeleton

Festa, LK, PhD^1^, Fandino Pachon, N, BS^2^, Grinspan, JB, PhD^1^, Jordan-Sciutto, KL, PhD^2^; ^1^Neurology, Children’s Hospital of Philadelphia, Philadelphia, PA 19104^2^Oral Medicine, University of Pennsylvania, Philadelphia, PA 19104.

Oligodendrocyte precursor cells (OPCs) and oligodendrocytes (OLs) must undergo dramatic cytoskeleton rearrangement to differentiate and produce myelin. Alterations in filamentous actin (F-actin) are highly sensitive to changes in intracellular Ca2+; however, while research has focused primarily on Ca2+ channels present on the plasma membrane, less is known about the role of intracellular Ca2+ stores such as the lysosome. The transient receptor potential mucolipin 1 (TRPML1) is the main Ca2+ exporter on the lysosome and interacts with the Rho GTPase, Rac1, a key regulator of the actin cytoskeleton. Intriguingly, inactivating mutations of TRPML1 results in the rare neurodevelopmental disorder mucolipidosis type IV, which is characterized by white matter abnormalities and hypomyelination. Taken together, we hypothesized that activation of TRPML1 is required for the actin cytoskeleton changes that underlie process extension. We found that activation of TRPML1 resulted in striking morphological alterations, including increased OL process number, length, and complexity. These changes were accompanied by a significant increase in F-actin staining intensity and F/G-actin ratio. Lastly, we demonstrated that stimulation of TRPML1 rapidly activates the Rac1/PAK pathway revealing a potential mechanism by which TRPML1 controls the OL actin cytoskeleton. Taken together, our work highlights a previously unknown function of TRPML1 in modulating OL function and has implications not only for homeostatic regulation but also disease states where lysosomal function is known to be dysregulated.

Supported by National Multiple Sclerosis Society (TA-2507-44763); NIMH (MH126773); NIMH (MH098742).

#### Progranulin Exerts Neurotrophic Effects by Acting in Lysosomes of Neurons and Glia

Arrant, A, PhD, Assistant Professor, Dept. of Neurology, University of Alabama at Birmingham, Birmingham, AL 35294.

Genetic variants of progranulin (GRN) that mildly reduce progranulin expression are associated with increased risk for Alzheimer’s and Parkinson’s diseases. GRN mutations that cause progranulin haploinsufficiency cause frontotemporal dementia. Progranulin is a secreted pro-protein that can engage in extracellular signaling before being taken up and trafficked to lysosomes, where it promotes lysosomal activity. Progranulin has neurotrophic and anti-inflammatory effects, but it is not clear if these effects are mediated by lysosomal actions or by extracellular signaling. To address this question, we generated a non-secreted, lysosome-targeted form of progranulin (L-PGRN), comprising progranulin fused to the transmembrane domain and cytosolic tail of LAMP-1. L-PGRN was trafficked to lysosomes and proteolytically processed and corrected lysosomal abnormalities in progranulin knockout cells. We then investigated whether L-PGRN maintained progranulin’s neurotrophic effects. In rat primary cortical cultures, we found that L-PGRN mimicked progranulin’s protective effects against excitotoxic doses of NMDA. This effect was cell autonomous and mediated by modulating neurons’ autophagic response to NMDA. In rat primary hippocampal cultures, we found that L-PGRN also mimicked progranulin’s pro-growth effects. This effect was non-cell autonomous and mediated by changes to factors secreted by astrocytes. These results show that progranulin acts in lysosomes to promote neuronal growth and survival, but that these effects are mediated by distinct mechanisms.

Supported by NIH R01 NS128031.

### Bill Narayan Memorial Lecture

#### Lysosomes Serve Important Roles in The Pathogenesis of Neurodegenerative Diseases, As Well As the Safety and Efficacy of Modern Pharmacotherapeutics

Geiger, J, PhD; Chester Fritz Distinguished Professor, Department of Biomedical Sciences, University of North Dakota School of Medicine and Health Sciences, Grand Forks, ND 58203.

The greater lysosomal system is composed of endolysosomes. These acidic organelles regulate physiologically important processes including autophagy and they have been implicated in the pathogenesis of diverse pathological disorders. Nevertheless, even though others and we have shown that diverse insults induce endolysosome de-acidification and cause morphological and functional features of lysosomal stress responses (LSR) that are upstream of effects on other organelles, these other organelles continue to receive far more attention. Following LSR, ferrous iron released from endolysosomes is sufficient to induce increases in cytosolic and mitochondrial iron and reactive oxygen species, mitochondrial membrane depolarization, and cell death; effects all prevented by endolysosome acidification and/or iron chelation. Thus, insult-induced effects on mitochondria may be secondary to and downstream of insult-induced effects on endolysosomes. However, effects on mitochondria can feedback and negatively affect endolysosomes because increases in reactive species can activate endolysosome redox-sensitive ion channels thus potentiating the pathological effects of insult-induced LSR. Further, 75% of licit and illicit drugs have the physico-chemical property of being weak-base drugs and because of their “ion trapping” in acidic organelles they induce LSR. Thus, unless cell biology is irrelevant, studies of insult-induced changes should include studies of the greater lysosomal system.

Supported by R01MH119000, 2RO1DA032444, P20GM139759)

### Symposium 7: Molecular mechanisms of RNA viruses induced neurodegeneration

**Co-Chairs**: Carlos Pardo, MD, Professor, Johns Hopkins University School of Medicine,

Pankaj Seth, PhD, Senior Professor, National Brain Research Center

#### Antisense Oligonucleotides as Broadly Effective Inhibitors of Mosquito-Borne Flaviviruses

Henderson, LJ, PhD^1^, Lee, ED, BS^1^, McDonald, V, MS^3^, Reed, MG^4^, Seth, P, PhD^2^, Wang, T, PhD^3^, Nath, A, MD^1^; ^1^Section of Infections of the Nervous System, National Institute of Neurological Disorders and Stroke, National Institutes of Health, Bethesda, MD 20892 ^2^Department of Cellular and Molecular Neuroscience, National Brain Research Centre, Manesar, 122052 ^3^Translational Neuroscience Center, National Institute of Neurological Disorders and Stroke, National Institutes of Health, Bethesda, MD 20892^4^ College of Arts and Sciences, The Catholic University of America, Washington, DC 20064.

Flaviviruses are an ongoing and expanding public health concern worldwide. Flaviviruses such as dengue (DENV), Zika (ZIKV) and West Nile (WNV) infect an estimated 400 million individuals per year, with DENV alone accounting for over 100 million cases. Many DENV infections are mild or asymptomatic, but some individuals develop life-threatening complications such as liver injury and encephalitis. Subsequent infections with different DENV serotypes significantly increase the risk of severe disease. There is a critical need for effective antivirals that can be quickly deployed to combat future outbreaks. Antisense oligonucleotides (ASO) are small DNA/RNA molecules that can be designed against a variety of genomic targets. Furthermore, since ASOs can be stored for extended periods and have a long half-life in vivo, they can be quickly administered to areas of need as post-exposure treatment or pre-exposure prophylaxis. We aligned over 2,000 DENV sequences representing isolates from all four serotypes to identify conserved regions of the viral genome. We then screened a panel of ASOs designed against these putative targets in DENV-infected vero cells. These studies identified a highly conserved sequence within the 3’ untranslated region (UTR) of DENV that is retained in all four serotypes. Treatment with an ASO complementary to this sequence blocked viral replication in both 2D (vero cells) and 3D (human cerebral organoid) infection models. These results suggest that our ASO-based antiviral is a promising candidate for reducing the morbidity and mortality caused by dengue.

#### KCNA10 Autoantibodies and Endothelial Injury in Post-Covid Autonomic Dysfunction

Johnson, TP, PhD^1^, De Souza, DR, MS^1^, Elkahloun, A, PhD^2^, Johnson, K, PhD^3^, Safavi, F, MD, PhD^4^, Nath, A, MD ^1^; ^1^Section of Infections of the Nervous System, National Institute of Neurological Disorders and Stroke, NIH, Bethesda, MD 20892 ^2^Microarrays and Single-cell Genomics Core Facility, National Human Genome Research Institute, NIH, Bethesda, MD 20892 ^3^Bioinformatics Section, National Institute of Neurological Disorders and Stroke, NIH, Bethesda, MD 20892^4^Neuro-Immunopathogenesis Unit, National Institute of Allergy and Infectious Diseases, NIH, Bethesda, MD 20892.

Introduction: Autonomic dysfunction, most commonly postural orthostatic tachycardia syndrome (POTS), develops in 10–25% of individuals with long-COVID and is associated with reduced cerebral blood flow and cognitive impairment. Although autoimmune mechanisms are proposed, most patients lack defined pathogenic autoantibodies, limiting targeted therapy. We sought to identify autoimmune responses in patients with new-onset or worsening autonomic dysfunction following COVID-19 vaccination. Methods and Results: Immunoblotting of pooled sera from a post-vaccine discovery cohort (n=15) revealed dominant autoreactivity to a ∼60 kD antigen enriched in brain endothelial cells. Individual screening demonstrated endothelial-reactive antibodies in 26.7% (4/15) of patients, targeting a membrane-associated antigen. Unbiased protein array profiling identified 136 enriched targets compared with healthy donors, including 13 plasma membrane proteins. KCNA10 (Kv1.8) was identified as the 60 kD endothelial antigen; 6/10 participants with autonomic dysfunction and 0/10 healthy controls demonstrated KCNA10 immunoreactivity by immunoblot and proximity ligation. Anti-KCNA10 antibodies induced complement-mediated toxicity in brain endothelial cells but not neurons, which lack KCNA10 expression. Conclusion: These data define an autoimmune biotype of autonomic dysfunction characterized by KCNA10 autoantibodies targeting brain endothelial cells. Antibody-mediated endothelial injury would predispose vulnerable regions to hypoperfusion, providing a mechanistic basis for cognitive dysfunction and syncope.

Supported by NINDS.

#### The Role of Neuroimmune Factors in The Pathogenesis of Neuroinflammatory Disorders

Pardo, CA., MD; Division of Neuroimmunology and Neurological Infections, Johns Hopkins University School of Medicine, Baltimore, MD 21212.

Acute neuroinflammatory disorders are associated not only with life-threatening conditions that often require critical care but also with long-term neurological disability and sequelae, significantly increasing the clinical and economic burden on the healthcare system. Disorders such as encephalitis, myelitis, and acute demyelinating polyneuropathies may be caused by infectious agents (e.g., viruses, bacteria) or autoimmune mechanisms. These disorders are influenced by multiple factors, including environmental conditions (e.g., urban vs. rural settings, ecosystems, vectors), circulating pathogens, population characteristics (e.g., children vs. adults), immune status (immunocompromised vs. immunocompetent), and genetic susceptibilities. Although the diagnosis of neuroinflammatory disorders has traditionally focused on infectious causes, the discovery of autoimmune and paraneoplastic etiologies has revolutionized the study of these disorders over the past two decades, revealing them as common causes in both adult and pediatric populations. The introduction of unbiased, pathogen-agnostic approaches, such as metagenomic NGS analysis, has significantly improved diagnostic accuracy in the clinical setting for patients with suspected neuroinflammatory disorders. The development of massively parallel antibody profiling techniques, including Phage-ImmunoPrecipitation Sequencing (PhIP-Seq)-based assays such as VirScan and, more recently, Molecular Indexing of Proteins by Self-Assembly (MIPSA) assays, which enable the comprehensive detection of antibodies against viral or human peptide.

Supported by Bart McLean Fund for Neuroimmunology Research and National Institutes of Health (R01-NS110112 and R01-NS123712).

#### Molecular Mechanisms of Zika Virus Induced CNS Pathogenesis Using 2D and 3D Models of Human Brain Cells

Seth, P, PhD, Kaur, Guneet, PhD, Bhagat, Reshma, PhD; Molecular and Cellular Neuroscience, BRIC-National Brain Research Centre, Gurgaon, India 122052.

Although blood brain barrier (BBB) offers protection against the invasion by pathogens, several infections of central nervous system are reported due to breaching of the barrier. Zika Virus (ZIKV) causes microcephaly as it also infiltrates the placental barrier cells and targets neural stem cells (NSCs) in developing brain. We used primary cultures of human fetal brain derived neural stem cells (hNSCs) and brain microvascular endothelial cells (hBMECs) to study the effect of Zika virus (ZIKV) Envelop (E) protein on properties of NSCs and hBMECs, separately. We elucidate that ZIKV E protein disrupts the cellular homeostasis in hBMECs, affecting BBB, and microRNA circuitry and wnt pathway in NSCs, leading to quiescence in NSC proliferation. Our data suggests ZIKV E protein triggers ER stress in hBMECs, as evidenced by upregulation of BiP and specifically activates the IRE1-XBP1 and ATF6 branches of the unfolded protein response (UPR). Further we observed increased levels of phosphorylated IRE1α, spliced XBP1 (XBP1s), and subsequent downstream pro-apoptotic genes. Using live-cell calcium imaging, we find significant increase in cytosolic calcium levels, reflecting disrupted ER calcium homeostasis as well as upregulation of autophagic marker, LC3-II. In summary our findings provide novel insights into mechanisms that lead to ZIKV E induced microcephaly in infants and its contribution in endothelial dysfunction at BBB leading to the barrier disruption.

Supported by BRIC-National Brain Research Centre, Manesar and Department of Biotechnology, New Delhi, India.

### Symposium 8: Molecular Signatures of HIV Pathogenesis: Viral and Host Proteins and Therapeutic Frontiers

**Co-Chairs**: Prasun K. Datta, PhD, Associate Professor, Tulane University, and Tulane National Primate Research Center, Santhi Gorantla, PhD, Professor, University of Nebraska Medical Center, Omaha, NE

#### Spatial Proteomic Signatures of HIV in the Human Frontal Cortex are Associated with Neurocognitive Performance

Langford, D, PhD, Hasan, Iman, BS, Tice, Caitlin, PhD; Virtua Health College of Medicine and Life Sciences at Rowan University, Rowan Virtua Schools of Medicine and Translational Biomedical Engineering and Sciences, Stratford, NJ 08084.

Chronic HIV infection continues to negatively impact the brain via persistent inflammation, microglial activation, astroglial reactivity, synaptic alterations, and metabolic dysregulation. Thus, neurocognitive impairment remains highly prevalent in individuals living with HIV despite sustained plasma viral suppression. Molecular changes are not uniform in the brain as white and grey matter, and transitional cortical regions exhibit distinct susceptibilities to inflammatory signaling, myelin disruption, synaptic injury, and neurodegenerative processes. Understanding regional specificity of HIV-associated molecular alterations is critical to address the heterogeneity of cognitive outcomes in people with HIV. Existing proteomic and metabolomic studies provide insights into the molecular disturbances accompanying HIV-associated neurocognitive disorders. However, these studies used homogenized brain tissue or biofluids, obscuring the spatial organization of pathological signals. We used the NanoString GeoMx platform with high-plex digital spatial proteomics to map protein network alterations in anatomically defined microenvironments of the HIV-infected human brain. This enabled multiplexed profiling of immune, neuronal, synaptic, proteostasis, and neurodegeneration-related proteins in precisely selected regions of interest. Integrating spatial proteomic signatures with harmonized neuropsychological testing data from the NNTC allowed the first high-resolution characterization of regional protein network alterations in HIV and their relationship to cognitive performance.

Supported by NIMH, NINDS.

#### HIV-1 Integration and Capsid-Binding Host Factors: Who, When and How!

Dash, CV PhD, Center for AIDS Health Disparities Research, Department of Microbiology, Immunology, and Physiology, School of Medicine, Meharry Medical College, Nashville, TN 37221.

HIV-1 infection is dependent on the interaction of the viral capsid with host factors. The cleavage and polyadenylation specificity factor 6 (CPSF6) is one such capsid-binding host factor, whose cellular function is to regulate mRNA processing and polyadenylation. Initial work identified a truncated form of CPSF6 blocked HIV-1 nuclear entry. However, the full-length CPSF6 promotes integration targeting into gene dense regions of the host genome. Surprisingly, the effects of CPSF6 on the HIV-1 preintegration complex (PIC) that carries out integration is unknown. To study CPSF6’s role in PIC fu, we extracted PICs from cells- depleted of CPSF6 or expressing a CPSF6 mutant that cannot bind to CA. These PICs exhibited significantly lower integration activity and addition of CPSF6 protein restored the integration activity of PICs from the mutant cells. To solidify CPSF6’s role in PIC function, we inoculated with HIV-1 particles and quantified reverse transcription, 2-long terminal repeat (LTR) circles for nuclear entry, and integration and identified integration sites. Our results showed that disrupting CPSF6-CA binding reduced HIV-1 integration without reducing reverse transcription or nuclear entry. Further, the CPSF6-CA binding deficient mutant viruses N74D and A77V showed minimal integration defects in the CPSF6 mutant cells. Disruption of CPSF6-CA binding significantly retargeted viral DNA integration into lamina-associated domains (LADs) instead of the gene-dense SPADs. These results identify a direct role for CPSF6 in HIV-1 PIC function both in vitro and in infected cells.

Supported by NIH/NIAID.

#### Small Molecule Inhibitors of the HIV-1 Nef Virulence Factor as A New Approach to HIV Therapy

Emert-Sedlak, LA PhD and Smithgall, T.E., PhD, Microbiology and Molecular Genetics, University of Pittsburgh School of Medicine, Pittsburgh, PA 15219.

Existing antiretroviral drugs do not eradicate HIV-1 from infected individuals, necessitating life-long therapy and highlighting the need for alternative therapeutic strategies. The HIV-1 Nef accessory protein is a compelling yet underexplored therapeutic target because it is essential for high-titer viral replication in vivo, enables immune evasion of HIV-infected cells, supports latency and promotes AIDS progression. Our laboratory has discovered and developed small molecules that bind directly to Nef with nanomolar potency and exhibit potent antiretroviral activity. These compounds also reverse Nef-mediated MHC-I downregulation in HIV-infected primary CD4 T cells and restore susceptibility to killing by autologous CTLs. However, these occupancy-based inhibitors spare other Nef activities, including CD4 downregulation, motivating a shift in our approach toward complete functional antagonism through targeted Nef degradation. We designed proteolysis targeting chimeras (PROTACs) by linking existing Nef-binding chemotypes to Cereblon (CRBN) E3 ligase ligands, generating bifunctional molecules that form ternary complexes with Nef and CRBN in vitro and induce Nef ubiquitylation and degradation in T cells. Nef PROTACs reverse Nef-mediated downregulation of CD4 and MHC-I from the T cell surface and suppress Nef-dependent enhancement of viral replication, providing strong proof of concept for targeted degradation. Nef degraders represent a new class of antiretroviral therapeutics with the potential to re-engage adaptive immunity and facilitate clearance of latent viral reservoirs.

Supported by NIH AI152677.

#### HIV-1 Infection Induces Vif-Driven Sumoylation of Host RNA Splicing Factors Important for Proper Viral RNA Splicing

Kelenis, DPK, MD, PhD ^1^, Johnson, JRJ, PhD ^2^, Sidoli, SS, PhD ^3^, Emery, AE, PhD ^4^, Swanstrom, RS, PhD ^4^, Hawkins, LJH, BS ^1^, Mckie, KM ^1^, Pawar, TP ^1^, Goff, SPG, PhD ^1^; ^1^Biochemistry and Molecular Biophysics, Columbia University Irving Medical Center, New York, NY 10032^2^Microbiology, Icahn School of Medicine at Mount Sinai, New York, NY 10029^3^Biochemistry, Albert Einstein College of Medicine, New York, NY 10461^4^Biochemistry and Biophysics, University of North Carolina at Chapel Hill, Chapel Hill, NC 27599.

HIV-1 utilizes host cell post-translational modifications (PTMs) to facilitate production of infectious particles. These modifications include SUMOylation, a dynamically regulated PTM involving covalent attachment of small ubiquitin-like modifiers (SUMOs) to lysine residues of target proteins. SUMOylation modulates the activity of thousands of proteins and multiple fundamental host cellular pathways, including pathways known to be exploited by HIV-1. Although the SUMOylation of several host and viral proteins in HIV-infected cells has been described, SUMOylation had not been explored by large-scale proteomics in the context of HIV infection. We performed a proteome-wide mass spectrometry (MS)-based screen to identify proteins that are SUMOylated in response to HIV-1 infection. Infection with HIV-1 led to the widespread increased SUMOylation of heterogeneous nuclear ribonucleoprotein (HNRNP) A/B family members, a family critical for the regulation of host cell RNA metabolism, including alternative splicing. Interestingly, this phenotype was driven by expression of Vif, suggesting an unexplored function for this protein. Depletion of HNRNPA/B proteins led to altered splicing of HIV-1 viral RNAs and dramatically reduced HIV-1 infectivity. We are currently utilizing non-SUMOylatable HNRNPA/B mutants to test the functional consequences of their SUMOylation on the alternative splicing of HIV-1 mRNAs. Together, our data point to a novel mechanism involving HIV-1-induced SUMOylation of host RNA splicing factors as a means to regulate HIV-1 splice variant production.

Supported by HIV RNA (CRNA) Grant# U54 AI170660, Herbert Irving Comprehensive Cancer Center (HICCC) Grant #P30CA013696, NIH 1 R01 AI178848.

### Banquet

**Introduction of speaker:** Linda Chang, MD, MS

#### Viruses and Neurodegenerative Diseases: Discovery of the Enemies Within.

Nath, A, MD, Clinical Director, NINDS, Senior Investigator, Section of Infections of the Nervous System, Section of Infections of the Nervous System, Division of Neuroimmunology and Neurovirology, NINDS, NIH, Bethesda, MD 20892.

Through the process of evolution, the human genome has acquired multiple retroviruses. Nearly 8% of the human genome is of viral origin. They have acquired multiple mutations and cannot form a complete viral particle. Scattered throughout the genome are multiple open reading frames for each of the retroviral proteins. They have been adapted to play a critical role in embryonic and brain development. However, they are mostly silenced in adults. Reactivation of these elements in adults can be detrimental and have been implicated in the pathogenesis of tumors and autoimmune disorders. We discovered that one of these viruses, HML-2, is reactivated in brain and spinal cord of patients with amyotrophic lateral sclerosis. Viral RNA and envelope protein can be detected in the blood and/or spinal fluid of these individuals. Antibody titers to the virus correlate inversely to survival. Even though there is evidence for epitope spreading for the immune response to the envelope protein in ALS. The envelope protein is neurotoxic in vitro and transgenic animals that express the envelope protein of the virus develop degeneration of motor neurons similar to ALS. We have identified CD98HC as the receptor for the envelope protein. The mechanism of reactivation of the virus and spread along anatomical pathways is poorly understood. Existing antiretroviral drugs are only partially effective against the virus. We and others are developing multiple therapeutic approaches aimed at silencing the virus along different stages of its lifecycle and at boosting the immune responses directed against it.

Supported by National Institute of Neurological Disorders and Stroke/ ZIA NS003130.

## Wednesday, May 6^th^, 2026

### Symposium 9: From Stigma to Science: Unpacking HIV and Cannabis Use

**Co-Chairs:** Barkha J. Yadav-Samudrala, PhD and Sylvia Fitting, PhD, Professor, University of North Carolina, Chapel Hill, NC

#### Delineating The Impact of Phytocannabinoid Exposure on HIV-Associated Neurocognitive Impairment: Insights From the HIV-1 Transgenic Rat Model.

Ayoub, S, PhD, Postdoctoral Scholar, Department of Psychiatry, University of California San Diego, San Diego, CA 92121.

Neurocognitive impairment remains prevalent and largely untreated among people living with HIV (PLWH). Cannabis use is also common in this population, yet its impact on HIV-associated neurocognitive impairment (NCI) remains unclear. While cannabis use has been linked to cognitive deficits in the general population, studies in PLWH more often report null or beneficial effects, suggesting the influence of factors such as usage patterns and cognitive domain assessed. Notably, isolated cannabis constituents (cannabinoids) can exert distinct – and sometimes opposing – effects on cognition and brain function, indicating that cannabis chemical composition may critically shape its effects on cognitive outcomes in HIV. To address this gap, we determined the impact of two major cannabinoids, Δ9-tetrahydrocannabinol (THC; 0.3 and 3 mg/kg) and cannabidiol (CBD; 0.3 and 3 mg/kg), on HIV-associated NCI under well-controlled experimental conditions using the HIV-1 transgenic rat model. Cross-species cognitive tasks were utilized to enhance translational relevance, including measures of risk-based decision-making, learning, cognitive flexibility, and effortful motivation. We found that THC recapitulated function-dependent cognitive effects reported in PLWH cannabis studies, whereas CBD produced minimal effects on cognition. Together these data support the use of the HIV-1 transgenic rat for evaluating cannabinoid-specific effects on HIV-associated NCI and suggest that THC may primarily drive cannabis-related cognitive effects in this population.

Supported by NIDA R01DA051295.

#### Real World Considerations for Cannabinoid Based Therapies During HIV

Ellison, AL, BSc, MSc, School of Medicine, Emory University, Atlanta, Georgia, 30307

Select cannabinoid compounds, such as cannabidiol (CBD) and D9-tetrahydrocannabinol (THC), interact with endogenous endocannabinoid receptors to elicit downstream immunomodulatory and other effects. To better understand effects of cannabinoids on endocannabinoid receptors in brain, we evaluated the impact of CBD and THC on endocannabinoid receptor gene expression in brains of two animal models. With oral CBD administration in a rhesus macaque model of SIV and THC vapor administration in a rat model system we found clear changes in endocannabinoid receptor expression regardless of the model, route of administration, cannabinoid, or disease state. However, specific changes in receptor expression were dependent on the receptor and brain region assessed. For example, CBD administration in rhesus macaques increased expression of endocannabinoid receptor 1 (cnr1) in all brain regions evaluated compared to SIV alone, but the same treatment selectively decreased transient receptor potential vanilloid 1 (trpv1) below the limit of detection in a singular brain region. Moreover, THC administration in rats elicited differential changes in expression across brain regions, increasing fatty acid amide hydrolase (FAAH) in dorsal striatum and hippocampus compared to vehicle, but decreasing this receptor in nucleus accumbens, frontal cortex, hypothalamus, and thalamus. These findings underscore the need for additional research to further elucidate effects of cannabinoids in healthy brain and in the context of disease, potentially leading to their safe and effective use in treating disease.

Funding: NIH F31 DA058562; R01 DA052859; U01 DA058527, Center for AIDS Research at Emory University P30 AI050409; T32 AI157855. Emory National Primate Research Center Grant No. ORIP/OD P51 OD011132; U42 PDP11023

#### Impact of Cannabis Use on the Viral Reservoir and Immune Cell Gene Expression in People with HIV on Antiretroviral Therapy.

Browne, EP, PhD^1^, Bhatt, U^1^, Murdoch, DM^2^, Margolis, DM^3^, Rudin, CD^4^; ^1^Department of Genetics, University of North Carolina at Chapel Hill, Chapel Hill, NC 27599 ^2^Department of Medicine, Duke University, Durham, NC 27710 ^3^Department of Medicine, University of North Carolina at Chapel Hill, Chapel Hill, NC 27599 ^4^Department of Computer Science, Duke University, Durham, NC 27705.

Cannabis (CB) use is frequent amongst people with HIV (PWH), but the impact of CB on HIV infection is unknown. PWH on antiretroviral therapy (ART) maintains a viral reservoir that is resistant to ART and elevated levels of chronic inflammation. We performed detailed HIV reservoir analysis and immunophenotyping on two cohorts of people with HIV on antiretroviral therapy (ART), including cannabis users and non-users. Flow cytometry indicated that cannabis using PWH exhibit elevated levels of naïve T cells and reduced levels of activated and exhausted T cells compared to non-using PWH. Bulk RNA sequencing (RNA-seq) on peripheral CD4+ T cells, as well as quantified plasma immune marker levels identified three distinct participant transcriptomic clusters, defined by differential expression of genes regulated by the inflammatory transcription factor NF-kB. Moderate cannabis use was associated with a lower inflammation profile, while heavy use was associated with a proinflammatory profile. Strikingly, cannabis use was associated with a significantly smaller HIV reservoir and with reduced levels of viral reservoir expression during ART. In vitro stimulation of peripheral blood mononuclear cells (PBMCs) from people with HIV indicated that THC exposure rapidly downregulates a module of inflammasome-regulated gene expression in monocytes, including IL-1 beta. These findings reveal that cannabis use is associated with distinct alterations to the viral reservoir and with the transcriptomic phenotype of immune cells in people with HIV.

Supported by NIDA: R33DA053599, R33DA059918, R01DA064083.

#### The Gut-Brain Connection: How Phytocannabinoids Supplement HIV Treatment to Reduce Chronic Inflammation.

Mohan, M, DVM, MS, PhD, Southwest National Primate Research Center, Professor, Texas Biomedical Research Institute, San Antonio, TX

Despite suppressive antiretroviral therapy (ART), people with HIV frequently manifest HIV-associated neurocognitive disorders (HAND). HAND is proposed to be mainly driven by chronic neuroinflammation mediated by activated microglia and perivascular macrophages. Emerging evidence also highlights the microbiota-gut-brain axis (MGBA), where intestinal dysbiosis and increased permeability exacerbate neuroinflammation. In longitudinal studies using the ART-suppressed SIV-infected rhesus macaque model, we investigated the efficacy of chronic low-dose Δ9-tetrahydrocannabinol (THC) on neuroinflammation and the MGBA. Our findings demonstrated that THC attenuated neuroinflammation and reduced dysbiosis via three key mechanisms: Transcriptomic Regulation: THC decreased the expression of genes linked to type-I interferon responses and glutamate excitotoxicity in the basal ganglia, a brain region critical for motor and cognitive function. Metabolic Shift: THC increased plasma endocannabinoid levels and inhibited the indoleamine 2,3-dioxygenase pathway. This shunts tryptophan metabolism away from the neurotoxic kynurenine pathway toward neuroprotective metabolites like serotonin and indole-3-propionate (IPA). Microbiome Homeostasis: THC-treated macaques maintained higher levels of beneficial Firmicutes and Clostridia that produce potent anti-inflammatory short-chain fatty acids and indole-metabolites like IPA. Collectively, these data suggest that cannabinoids may serve as a viable therapeutic strategy to mitigate HAND by reducing neuroinflammation and enhance signaling along the MGBA.

Supported by NIH/NIDA R01DA042524, R01DA052845, R01DA059874.

### Symposium 10 – SNIP Members’ Symposium

**Co-Chairs:** Susmita Sil, PhD, Assistant Professor, University of Nebraska, Medical Center, Omaha, NE and

Richard J. Noel, PhD., Chair & Professor, Ponce Health Sciences University, Ponce, PR

#### Role of Endolysosomes in SARS-CoV-2 Spike-Induced Cellular Senescence in Human Astrocytes

Chen, X, MD, PhD, Hasler, WA, PhD, Datta, G, PhD; Department of Biomedical Sciences, University of North Dakota School of Medicine and Health Sciences, Grand Forks, ND, 58203 United States.

COVID-19, caused by SARS-CoV-2, is associated with long-lasting neuropsychiatric and cognitive impairments, often referred to as neurologic post-acute sequelae of SARS-CoV-2 infection (Neuro-PASC). Emerging evidence suggests that the COVID-19 brain exhibits signs of aging and cellular senescence, which could lead to neurodegeneration and the development of neuro-PASC. In this study, we investigated the role of the SARS-CoV-2 spike protein S1 in inducing a senescence-like phenotype in human astrocytes. Our results show that S1 induces the development of hallmarks of cellular senescence including increased release of senescence-associated secretory phenotype (SASP) factors (IL-6 and CCL2), enhanced SA-β-gal activity, and elevated p16 levels. Additionally, S1 causes endolysosome damage including increased pH, altered morphology, and membrane leakage, as well as the release of galectin-3 and cathepsin B. Importantly, the multibasic motif (RPRR) of S1 is critical for S1-induced endolysosome damage and cellular senescence. Mechanistically, we identified toll-like receptor 7 (TLR7) as a key mediator of S1-induced endolysosome damage and cellular senescence; S1 interacts with TLR7, activation of TLR7 alone replicates the impact of S1, and TLR7 knockdown attenuates S1-induced senescence-like phenotype in astrocytes. Our findings provide novel mechanistic insights into the pathogenesis of neuro-PASC.

Supported by NIH: MH134592 and DA059280

#### Perinatal Fentanyl Exposure Reprograms Microglial Development and Neuroimmune Signaling Across Mesocorticolimbic Circuits

Olusakin, J, PhD, Nicot, A, Mclnerney, J, Franco, D, Bertelsen, K, Lobo, MK; Neurobiology, University of Maryland School of Medicine, Baltimore, MD, 21201 United States.

Prenatal opioid exposure continues to increase worldwide and is associated with neonatal opioid withdrawal syndrome and long-term neurodevelopmental risk. Further, evidence suggests that opioids perturb early neuroimmune signaling, but how such disruptions unfold across development remains unclear. Here, using a mouse model of perinatal fentanyl exposure (PFE), we previously identified sex-specific behavioral adaptations in adolescent mice with males display heightened anxiety-like behavior and females exhibiting reduced motivation to groom. To explore neuroimmune mechanisms that may underlie these outcomes, we performed a custom NanoString transcriptomic analysis across six developmental timepoints (from P1 to P35) from the prefrontal cortex, nucleus accumbens, and ventral tegmental area. PFE produced coordinated, sex- and region-specific dysregulation of microglial homeostatic and activation markers, including P2ry12, Cx3cr1, Csf1r, Cd68, Il18, and S1pr1 suggesting altered microglial maturation and immune priming during critical periods of circuit formation. Ongoing RNAscope analyses aim to validate cell-type specificity of molecular markers across mesocorticolimbic circuits, and future studies will employ CRISPR-based targeting of candidate genes to test their causal contribution to PFE-induced behavioral dysfunction.

#### SIV/HIV-Induced Lipid Dysregulation Induce Neuroinflammation and Tissue Damage, and the Role of Pannexin-1 in Neuro-HIV

Ajasin, DA, PhD^1^, Devin, SD, BS^2^, Prideaux, BD, PhD^3^, Eugenin, EE, PhD^2^, Gonzalez-Garcia, HG, MD^1^; ^1^Department of Internal Medicine-Infectious Disease Division, University of Texas Medical Branch, Galveston, TX, 77555 United States. ^2^John Sealy School of Medicine, University of Texas Medical Branch, Galveston, TX, 77555 United States. ^3^Department of Neurobiology, University of Texas Medical Branch, Galveston, TX, 77555 United States.

HIV-associated cardiovascular disease (HIV-CVD) and HIV associated neurocognitive disorder (HAND) are two of the most common HIV comorbidities seen in people living with HIV (PLWH), which are associated with chronic inflammation despite low viremia in PLWH. To understand how inflammation in the absence of viremia contributes to these comorbidities, we performed spatial unbiased analyses of lipids from post-mortem heart and brain tissues from both HIV-infected individuals and SIV-infected non-human primates (NHPs). Using mass spectrometry imaging (MSI), confocal imaging and immunoblotting, we identified arachidonic acid (AA) as one of the major lipids that changed significantly between the uninfected and infected tissues, which is associated with significant elevation in the levels of critical enzymes in arachidonic acid catabolic pathways, and inflammation. Interestingly, inhibition of an hemichannel (HC), Pannexin-1 (Panx-1) in the NHP led to significant reduction in the level of arachidonic acid and the expression of critical enzymes in the same pathways. While inhibition of Panx-1 HC does not reduce all inflammation, here we observed that arachidonic acid-associated inflammation is dampened in both the heart and the brain of both human and NHP samples. This result suggests that inhibition of Panx-1 HC could be therapeutic and ameliorate HIV-CVD and HAND.

#### Chronic HIV Infection Alters Neuronal Firing and Neurovascular Coupling in Reward Pathway Relevant Areas in Awake-Behaving Animals

Andrews, AM, PhD, Myers, C., MS, Fortney, SL, Shukla, P, Ramirez, SH, PhD; Pathology, Immunology & Laboratory Medicine, University of Florida, Gainesville, FL, 32610 United States.

Although people with HIV are living longer, HIV-associated neurocognitive disorder (HAND) prevalence remains high and can be exacerbated by drug use. However, the underlying mechanisms and synergistic effects of drugs of abuse remains unresolved. Neurovascular coupling (NVC) is a complex physiological process where active brain regions regulate blood flow to supply oxygen and nutrients to meet neuronal metabolic (energy) demands. NVC imbalances can lead to symptoms of cognitive impairment. Imaging studies in PWH suggests a dysfunctional NVC. However, how the NVC is impacted in anatomical areas relevant to the reward pathway in the context of HIV infection and drugs of abuse remains unexplored. New intravital miniscopes combined with genetically encoded activity-sensing fluorescent indicators as well as fluorescent tracers in the vasculature allows for the measurement of NVC in awake freely behaving animals. Applying this technology to the study of HIV and drugs of abuse enables cellular/mechanistic investigation into the changes in neurovascular function and could open the door to a better understanding of possible contributors to HAND neuropathology. We have performed pilot studies to image reward pathway relevant brain regions (cortex, nucleus accumbens NAc) of humanized mice with and without HIV infection. CD34+ humanized NOD scid gamma (NSG) mice were implanted with cranial windows or microlenses to the NAc infected with HIVJR-CSF. Animals were then imaged multiple times over the course of 4 months and showed hyperactive neuron firing and impaired vasodilation responses

Supported by DP2DA05617

#### HIV-relevant Inflammatory Stimuli and Antiretroviral Therapy Exposure Induces Reactive Astrocytes Driven by Glycolysis and Resulting in the Secretion of Neurotoxic Compounds

Halcrow, PW, PhD, Spencer, M, BS, Fields, JA, PhD; Dept. of Psychiatry, University of California, San Diego, La Jolla, CA, 92037 United States.

HIV infection often results in HIV-associated neurocognitive impairment (HIV-NCI) in approximately 50% of people with HIV (PWH) despite effective antiretroviral therapy (ART). HIV-NCI lacks effective treatment because the mechanisms of HIV-induced neuronal injury are poorly understood. Neuropathogenesis in HAND involves metabolic changes, with increasing evidence for a role by astrocytes. Astrocytes are targeted by HIV, inducing reactive astrogliosis—a common characteristic in postmortem brain tissues from decedents with HIV-NCI. Activated astrocytes contribute to HIV neuropathogenesis by altering the CNS microenvironment and releasing proinflammatory cytokines. While some molecules released by astrocytes are neurotrophic, evidence supports astrocytes as key mediators of HIV-induced neuronal injury. However, the metabolic mechanisms and secreted neurotoxins underlying activated astrocyte-induced neuronal injury remain unclear. Methods: Using primary astrocytes and neurons exposed to HIV-relevant stimuli (HRS including IL-1β, TNF-α, Tat, gp120, TFV, and DTG), we determined metabolic changes and neurite length with qPCR, ICC, and gene knockdown. Results: We found that (1) HRS induced reactive astrocytes resulting in (2) a metabolism driven by glycolysis, (3) astrocyte conditioned media (CM) induced neurite retraction, and (4) de-proteination of the CM blocked neurite retraction. Conclusion: This study begins to identify mechanisms by which HIV might induce glycolytic-driven astrogliosis that results in secreted neurotoxins that cause neuronal injury.

#### Methamphetamine and HIV-1 Infection Activate Innate Sensing in Microglia Through the Inflammatory cGAS-STING pathway

Mamede, JI, PhD^1^, Zayas, JP, PhD^1^, Jana, A, PhD^1^, Narasipura, S, PhD^1^, Gambut, S, MS^1^, Yoh, Sunnie, PhD^2^, Al-Harthi, L, PhD^1^; ^1^Department of Microbial Pathogens and Immunity, Rush University Medical Center, Chicago, IL, 60612 United States. ^2^Department of Immunology and Microbiology, Scripps Research, La Jolla, CA, 92037 United States

A significant proportion of people living with HIV/AIDS (PLWH) develop HIV-associated neurocognitive disorders (HAND). While combination antiretroviral therapy (cART) reduces morbidity and mortality, chronic neuroinflammation persists in the CNS, especially with comorbid methamphetamine (Meth) use, yet mechanisms remain unclear. Microglia drive HIV neuroinflammation. Using iPSC-derived microglia (iMGL) and microglia-containing cerebral organoids (CO-iM), we show that Meth and HIV-1 converge on the cGAS-STING pathway under brain-relevant cART. Meth causes mitochondrial dysfunction and cytoplasmic leakage of mitochondrial DNA (mtDNA) that colocalizes with cGAS puncta; MitoTracker (mass/activity) and SeaHorse profiling support reduced mitochondrial activity during binge-like re-dosing. HIV triggers rapid cGAS/STING activation after entry, consistent with sensing early reverse transcription products and capsid integrity loss. Low brain cART levels fail to block cGAS/STING activation. cGAS/STING knockout or STING antagonism (H-151) reduces ISG54/IFNÎ^2^ induction and cytokine release. Conditioned media from MethÂ±HIV-activated microglia skews astrocytes toward inflammatory phenotypes and impairs neurons, reducing synaptic markers (Synapsin-1, PSD95) and connectivity. In CO-iMs, cyclic multiplex IF confirms coordinated microglial cGAS/STING activation, astrocyte inflammation, and neuronal dysfunction. These data implicate Meth-induced cytoplasmic mtDNA release as a driver of cGAS/STING activation that sustains NeuroHIV inflammation and represents a potential therapeutic target.

Supported by R33DA058348

#### EcoHIV Infection Impairs Extinction Learning and Dysregulates Corticostriatal Microglia

Namba, MD, PhD, Yadlapalli, A, Wiafe-Jackson, J, Goldberg, SL, MS, Jackson, JG, PhD, Barker, JM, PhD; Department of Pharmacology & Physiology, Drexel University College of Medicine, Philadelphia, PA, 19102 United States.

Human immunodeficiency virus (HIV) remains a persistent public health dilemma worldwide that is associated with neurocognitive impairment (NCI). Many preclinical studies suggest that HIV can dysregulate reward learning, which may contribute to the common co-occurrence of substance use disorders with HIV. Nevertheless, very few studies have interrogated what specific reward learning constructs are impacted by chronic HIV infection, and elucidating these neurobehavioral processes is crucial to identifying effective treatment targets for treating NCI in the context of HIV. Here, we examined the impact of chronic HIV infection on sucrose-seeking behavior using the EcoHIV rodent model in male and female mice. EcoHIV had no impact on sucrose self-administration but significantly impaired extinction learning. EcoHIV also significantly reduced responding for sucrose among females but not males in a progressive ratio test. Examination of corticostriatal microglia via Iba1 immunolabeling revealed a significant reduction in Iba1+ cells within the infralimbic cortex of male mice due to EcoHIV infection. Within the nucleus accumbens (NAc), sex-specific inhibitory effects of EcoHIV infection on Iba1+ cell density were observed along with shifts in microglia morphology. Altogether, these results indicate that EcoHIV infection can enhance sucrose-seeking behavior under extinction conditions, that this is not due to enhanced motivation for sucrose, and that such behavioral impairments may be sex-specifically mediated by microglial dysregulation within corticostriatal reward circuitry.

Supported by NIH grants DP2DA051907 (JMB), DP2DA051907-01S1 (JMB), & F32DA060768 (MDN

#### HIF-1 siRNA Encapsulated Extracellular Vesicle Therapy Protects Against HIV-Associated Neurological Deficits

Ray, S, PhD, Kumar, M, Chemparathy, DT, PhD, Dash, PK, PhD, Sil, S, PhD; Pharmacology & Experimental Neuroscience, University of Nebraska Medical Center, Omaha, NE, 68198 United States.

HIV-associated neurocognitive disorders (HAND) persist in almost 50% of people with HIV on antiretroviral therapy, often accompanied by Alzheimer’s-like pathology, with Aβ accumulation reported in 29% of middle-aged individuals with HIV on antiretroviral therapy. We previously demonstrated that hypoxia-inducible factor-1α (HIF-1α) is a key driver of HIV-1 Tat-mediated amyloidogenesis in astrocytes, with amyloid-enriched astrocyte-derived extracellular vesicles (ADEVs) leading to Alzheimer’s-like pathology, synaptic damage, and behavioral changes in naive mice. However, whether selectively inhibiting astrocytic HIF’α can reverse HIV-driven Alzheimer’slike pathology and neurodegeneration in vivo remains unexplored. We hypothesized that intranasal delivery of ADEVs packaged HIF-1α-targeting siRNA could mitigate HIV-induced neuropathology. Using HIV-infected CD34+ NSG humanized mice, we administered HIF-1α siRNA-loaded ADEVs intranasally, achieving targeted delivery to the hippocampus and cortex. This intervention reduced expression of HIF-1α, amyloid precursor protein, AβmOC64, amyloid fibrils, phosphorylated tau, dampened glial activation, partially restored loss of synaptic proteins in the brain regions & partially improved impairments in spatial memory and anxiety-like behavior. These findings establish HIF-1α as a central regulator of HIV-associated Alzheimer’ss like pathology and validate ADEV-mediated HIF-1α siRNA delivery as a targeted, non-invasive therapeutic strategy for HAND and other Alzheimer’s-like disorders.

Supported by R21AG069541(NIH) and UNMC startup funds to S Sil.

#### HIV Suppresses Colonic B-Catenin, Alters the Microbiome, and Induces Gut Barrier Leakiness That Is Recapitulated by the Microbiome Independent of HIV and Reversed by B-Catenin Activation

Virdi, AK, PhD, Narasipura, S, PhD, Olivares, LJ, BS, Al-Harthi, L, PhD; Department of Microbial Pathogens and Immunity, Rush University Medical Center, Chicago, IL, 60612 United States.

HIV-mediated gut barrier dysfunction occurs early in infection and persists despite antiretroviral therapy, yet the mechanisms driving this pathology remain incompletely defined. Using a huPBMC-NSG mouse model, we show that human lymphocytes traffic to the gut within two weeks of engraftment and become HIV-infected. HIV infection induced functional gut barrier leakiness and altered the microbiota, assessed by FITC-dextran permeability and 16S rRNA sequencing. HIV-infected mice exhibited reduced microbial diversity, loss of Firmicutes (particularly Clostridia, key short-chain fatty acid producers) and expansion of Bacteroidota (Prevotella enriched), consistent with dysbiosis. In the proximal colon, HIV infection significantly downregulated active B-catenin and the tight junction protein ZO-1. Intracolonic administration of CHIR, a β-catenin agonist, restored B-catenin and ZO-1 expression and reversed functional leakiness. Notably, fecal microbiota transfer (FMT) from HIV-infected donors to antibiotic-treated HIV-negative recipients similarly reduced B-catenin and ZO-1 expression and induced gut leakiness, demonstrating that microbial communities or their products can impair barrier integrity independent of direct HIV infection. Together, these findings identify suppression of colonic β-catenin signaling as a central mechanism linking HIV-associated dysbiosis to gut barrier dysfunction and suggest β-catenin modulation as a therapeutic strategy to restore intestinal integrity in HIV disease.

### Symposium 11 - Local Symposium

**Co-Chairs:** Amanda Brown, Ph.D., Professor, Johns Hopkins University School of Medicine; Yajie Liang, PhD, Assistant Professor, University of Maryland School of Medicine, Baltimore, MD

#### Intravital Imaging Microglia Dynamics in the Live Mouse Brain.

Liang, YL, MB, PhD; Assistant Professor, Dept. Diagnostic Radiology and Nuclear Medicine, University of Maryland School of Medicine, Baltimore, MD

Microglia are the brain’s resident immune cells, constantly surveying the parenchyma and rapidly responding to injury, ischemia, and neoplastic invasion. Capturing these behaviors in vivo requires high-speed, volumetric imaging deep in scattering tissue, often in combination with targeted perturbations. In this talk, I will first introduce intravital two-photon strategies for microglia imaging in the mouse cortex, including chronic cranial windows, genetic labeling, and resonant-scanner microscopy. I will then highlight our recent development of tunable Bessel beam two-photon fluorescence microscopy (tBessel-TPFM), which converts slow 3D scans into fast 2D projections while allowing independent tuning of spatial resolution, axial coverage, and side-ring confinement, and uniquely maintains a stable focal center compatible with multi-wavelength stimulation. Using tBessel-TPFM, we map cerebral blood flow and neurovascular coupling across millimeter-scale volumes, quantify hemodynamic collapse in photothrombotic stroke, and perform kHz line scans in large vessels. Finally, I will focus on microglial process dynamics following single-cell two-photon ablation, where fast volumetric imaging combined with neural network–based denoising reveals previously unrecognized two-wave recruitment and coordinated extension–retraction patterns around lesions. Together, these platforms provide a powerful framework to study microglial behavior and to evaluate neuroimmune pharmacological interventions directly in the living brain.

Supported by NIH (R21AG077631; R03NS123733; R03NS128459; R21AG074978)

#### Cerebral Perfusion as a Biomarker for Alzheimer’s Disease

Ze Wang, PhD, Professor, University of Maryland School of Medicine, Baltimore, MD

Cerebral blood flow (CBF) is crucial to normal brain function, supplying oxygen and glucose to support neuronal metabolism, synaptic activity, and network integrity. Because the brain has limited energy reserves, it is highly sensitive to alterations in cerebral perfusion. Even mild or chronic reductions in CBF can disrupt neural function and impair brain homeostasis. Growing evidence indicates that cerebral hypoperfusion is closely linked to the etiology and progression of Alzheimer’s disease (AD). Reduced CBF has been observed in individuals at risk for AD, in prodromal stages such as mild cognitive impairment, and throughout the disease course. These findings suggest that perfusion abnormalities may represent an early and mechanistically relevant biomarker of AD. Arterial spin labeling (ASL) MRI enables noninvasive, quantitative measurement of CBF without the need for contrast agents or radiation, making it well suited for longitudinal and clinical studies. Advances in ASL techniques have improved sensitivity and reliability, facilitating the investigation of neurovascular dysfunction in aging and AD. Building on this framework, our work has focused on developing and applying advanced ASL-based methods to characterize cerebral perfusion alterations across the Alzheimer’s disease spectrum and to evaluate their utility as biomarkers for early detection and disease monitoring.

#### Reward Circuitry Microglia-Neuron Interactions Across the Lifespan in Disrupted Motivation

Lobo, Mary Kay, PhD; University of Maryland School of Medicine, Baltimore, MD 21201.

Environmental experiences that disrupt motivation and reward processing lead to neuronal structural adaptations in reward-circuit brain regions, including the nucleus accumbens. These neuronal adaptations—such as altered dendritic morphology and dendritic spine structure—can be shaped by microglial interactions with neurons. Our lab has identified changes in microglial contact onto specific nucleus accumbens neuron subtypes that exhibit atrophy during negative affective behaviors induced by social stress exposure. Microglia surrounding these neurons display phagocytic signatures. In parallel, we observe altered molecular profiles indicative of disrupted neuroimmune function in the nucleus accumbens. Additional studies examining conditions of stress or exposure to addictive drugs, either in adulthood or in utero, show that experiences inducing negative affective behaviors also result in altered neuroimmune signatures in the nucleus accumbens and other reward brain regions. Overall, our work is identifying unique microglia–neuron molecular signatures that contribute to the sculpting of neuronal adaptations underlying disrupted motivation and reward processing. These findings have implications for disorders characterized by impaired motivation, including substance use disorders and neuropsychiatric conditions.

#### Humanized Mouse Models That Enable the Development of Human Myeloid Cells: Opportunities for Targeting HIV Reservoirs in the Brain.

Heredia, A, PhD^1^, Pinzon-Burgos, E, MS^1^, Urquiaga, A, BS^1^, Munnawar, A, PhD^1^, Walczak, P, MD, PhD^2^, Chang, L, MD^2^

^1^Institute of Human Virology, University of Maryland School of Medicine, Baltimore, MD 21201; ^2^Department of Diagnostic Radiology and Nuclear Medicine, University of Maryland School of Medicine, Baltimore, MD 21201.

The development of persistent cellular reservoirs of latent HIV remains an obstacle to viral eradication because the virus rebounds after ART interruption. The main cellular reservoir is resting CD4 T cells, which is well known. In contrast, less is known about the cellular reservoirs of myeloid cells in liver, lung, lymph nodes, gut, urethra and brain. The brain reservoirs of myeloid cells (macrophages, microglia and dendritic cells) and astrocytes are difficult to study because brain can only be accessed at autopsy. Attempts are being made to evaluate HIV reservoirs, especially the brain, in humanized mouse models. Humanized mice, generated by transplantation of CD34 human hematopoietic stem cells into immunodeficient mouse strains, recapitulate key aspects of HIV infection in humans, including virus suppression by ART, establishment of virus reservoirs, and virus rebound following ART withdrawal. Humanization of the most commonly used mouse strains (NSG, NOG and NRG) results in robust development of human T cells (CD4 and CD8) but limited development of myeloid cells. This poor reconstitution of human myeloid cells is due, at least in part, to limited cross-reactivity of mouse and human cytokines The recent introduction of transgenes for human cytokines, including IL-3, GM-CSF, SCF, FLT3LG and IL34, into the genome of NSG and NOG mice have provided advanced humanized mouse models that reconstitute human myeloid cells and astrocytes in the brain. These newer humanized mouse models provide opportunities for targeting HIV reservoirs in the brain.

Supported by NIDA-DP1DA53719 and NIDA-R01DA056739.

#### Modeling Neural Degeneration to Enhance Brain Health in HAND

Royal, W, MD^1^, Sariyer, I, PhD^2^, Boatright, Jeffery, PhD^3^, Seiler, M, PhD^4^;

^1^Center for Brain Health Research, Morgan State University, Baltimore, MD 21251 ^2^Comprehensive NeuroAIDS Center, Temple University, Philadelphia, PA 19140 ^3^Department of Ophthalmology, Emory University School of Medicine, Atlanta, GA 30322 ^4^Physical Medicine & Rehabilitation, Ophthalmology & Visual Sciences, University of California, Irvine, Irvine, CA 92697 .

Impairment of taste, hearing, and vision can serve as early biomarkers of degenerative disorders, such as Alzheimer’s disease, and rehabilitation approaches that target these areas can be effective in slowing cognitive decline and improving brain function. Individuals with HIV-Associated Neurocognitive Disorders similarly develop significant impairment of these sensory functions, which may result from either virus- or treatment-related secondary complications or due to the primary effects of HIV infection. Retinal abnormalities caused by opportunistic infection, once found to occur in up to 75% of individuals with HIV/AIDS, are now rare due to the availability of treatment with combined antiretroviral therapy. However, abnormalities limited to the retina in the absence of an identifiable opportunistic process, termed HIV associated neuroretinal disorder (HIV-NRD), have been described in as many as 16% of individuals with HIV. HIV-NRD is associated with a higher risk of blindness and increased mortality. Preliminary studies performed using the HIV-1 transgenic rat model are providing insight into mechanisms that may lead to the development and progression of HIV-NRD. It is possible that there have been reductions in the incident risk of HIV-NRD with the most recently introduced anti-HIV treatment and management approaches. However, ongoing studies of HIV-NRD are critical for obtaining insights into developing approaches for preserving vision and improving the overall longevity and brain health of affected persons.

Supported by Veterans Administration/I50RX002358.

### Early Career Investigator Abstracts (See Symposium 3 above for Abstracts #1 - #12; #13-#22 below also Received the ECITA Award for their Posters):

#### 13. Stage-Dependent Transcriptomic Profiling of Human Cerebral Organoids with and without Binge Ethanol Treatment

Bishir, M, MS^1^, Pradeep, P^1^, Huang, W, MS^1^, Zhou, H, PhD^2^, Urso, CJ, PhD^2^, Sariyer, IK, PhD^3^, Chang, SL, PhD^1^;

^1^Institute of NeuroImmune Pharmacology, Seton Hall University, South Orange, NJ, 07079 United States. ^2^Department of Biological Sciences, Seton Hall University, South Orange, NJ, 07079 United States. ^3^Department of Microbiology, Immunology, Center for Neurovirology and Gene Editing, and Inflammation, Temple University Lewis Katz School of Medicine, PA, PA, 19140 United States.

Human cerebral organoids (hCOs) provide a physiologically relevant invitro model of early human brain development. Early pregnancy is a critical window for alcohol-induced disruption of fetal brain development. Among pregnant drinkers, 45% consume alcohol in first trimester and 60% are unaware of pregnancy until week four, emphasizing the need for early brain development models. Although hCOs up to 40 days resemble early fetal brain, stage-specific molecular changes and alcohol responses during maturation remain unclear. Here, we used hCOs with and without binge ethanol (EtOH, 50mM for 24h) to characterize transcriptomic changes across 10, 20, 30,40, 60, and 80 using RNA sequencing. Results from Days 40 and 80 showed that Day 40 hCOs were enriched for CNS development, synaptogenesis, and neurotrophic signaling, including BDNF, NTRK2, and ASCL1 centered networks. Transcription factors-PTF1A, ASCL1, BDNF, and PAX2-supported regional neural patterning and differentiation consistent with early brain development. In contrast, Day 80 hCOs exhibited transcriptomic signatures of maturation, including L1CAM-mediated axonal guidance, myelination, glutamatergic, GABAergic, opioid, and cholinergic signaling. EtOH treatment on Day 40 enhanced GABAergic while suppressing synaptogenesis signaling, neurexins and neuroligins. By comparison, Day 80 hCOs showed minimal transcriptional changes, with selective suppression of cholesterol biosynthesis. Together, these findings establish hCOs as a valuable invitro model for studying alcohol-mediated impairment of fetal brain development.

Supported by NIH Grants (U01-AA025964 and R03-AA031535).

#### 14. Testicular Toll with Viral Intrusion: Molecular Insights on The Impact of Chronic HIV-Infection on Male Fertility

Hediyal, TAH, PhD^1^, Stone, EMS, BS^1^, Shukri, OS, MD^1^, Schaal, VLS, BS^1^, Devanaboyina, MD, BS^1^, Foroughi, AF, BS^1^, Pradhan, PD, MD^2^, Yelamanchili, SVY, PhD^1^, Pendyala, GP, PhD^1^;

^1^Department of Anesthesiology, University of Nebraska Medical Center (UNMC), Omaha, NE, 68198 United States. ^2^Department of Pathology, Microbiology & Immunology, University of Nebraska Medical Center (UNMC), Omaha, NE, 68198 United States.

HIV infection remains a major global health concern, compromising immune function and increasing vulnerability to infection. Over time, fertility desire among people living with HIV has remained low, largely due to poor health, fear of transmission, and restrictive policies. In men, particularly during advanced disease progression, HIV has been associated with alterations in semen quality, including reduced ejaculate volume, impaired sperm motility, abnormal morphology, and increased sperm aneuploidy factors which all contribute to male infertility. This study investigated the molecular mechanisms underlying HIV-associated male infertility using a preclinical HIV transgenic (Tg) rat model. RT-PCR analysis revealed marked downregulation of CatSper 1-4 channel gene expression, accompanied by dysregulation of mitochondrial and inflammatory gene profiles in Tg testes. Histopathological examination demonstrated structural disruptions within the seminiferous tubules, while computer-assisted semen analysis (CASA) confirmed abnormal sperm morphology and reduced motility in Tg rats. Transcriptomic sequencing further identified novel gene signatures and disrupted pathways in Tg testes. Our findings indicate that HIV-1 infection impairs sperm production and function by interfering with ion channel regulation and perturbing broader molecular networks, ultimately contributing to infertility. Collectively, this study provides clinically relevant insights into reproductive complications in HIV-infected individuals, and the novel identified gene signatures may inform future therapeutics.

Supported by NIH R21DA058588, Lieberman Endowment and Department of Anesthesiology Development funds to G.P.

#### 15. Developmental Oligodendroglial and Myelin Abnormalities in A Non-Infectious Model of Neuro-HIV

Jeffries, MA^1^, Singhal, A^1^, Cruz-Berríos, M^2^, Romer, MA^1^, Jordan-Sciutto, KL^2^, Grinspan, JB^1^; ^1^Division of Neurology, Children’s Hospital of Philadelphia, Philadelphia, PA 19104^2^ Department of Oral Medicine, University of Pennsylvania, Philadelphia, PA 19104.

30-50% of people with HIV (PWH) exhibit neurocognitive dysfunction associated with persistent inflammation and white matter pathologies despite effective viral suppression. Adolescents undergoing myelination may be particularly susceptible to HIV white matter disruption. The noninfectious HIV-1 transgenic (Tg) rat model has an integrated partial HIV genome and an altered transcriptome suggestive of deficient myelination. We are using this model to examine the hypothesis that integrated HIV (iHIV) disrupts oligodendroglial development, altering myelin structure. Glial cultures from neonatal or 3-week-old HIV-1 Tg rats yielded fewer oligodendrocyte precursor cells (OPCs) than control cultures. Purified HIV-1 Tg OPCs exhibited reduced proliferation and impaired differentiation into oligodendrocytes (OLs). To determine if iHIV alters developmental myelin structure, we immunoblotted for myelin proteins from control and HIV-1 Tg brain regions at 3 or 9 weeks but found no changes. However, myelin is highly lipid-rich, and disrupted brain lipid metabolism results in myelin abnormalities and is predictive of cognitive decline in HIV. While lipidomics on whole brain myelin at 3 weeks showed no significant changes, HIV-1 Tg myelin at 9 weeks was significantly increased in diacylglycerols with specific increases in ceramides, phospholipids, and LPCs. Likewise, cholesterol was elevated at 9 weeks. These data suggest an acquired dysregulation in myelin lipid metabolism, which may be a therapeutic target to improve myelin integrity and cognitive function in PWH, particularly adolescents.

Supported by F32 MH135724, CHOP Supporting Promising Academic Researchers Fellowship, CHOP Bridge to Faculty Award.

#### 16. A BBB-Penetrating Nanocage Platform Remodels the Neuroimmune Microenvironment by Attenuating Dual Fibrotic and Immunological Barriers

Lee, DL, PhD^1^, Chung, SWC, PhD^2^, Kong, BK, PhD^1^, Suk, JSS, PhD^1^;

^1^Department of Neurosurgery, University of Maryland School of Medicine, Baltimore, MD, 21201 United States. ^2^1Center for Nanomedicine, Wilmer Eye Institute, Johns Hopkins University School of Medicine, Baltimore, MD, 21231 United States.

Aberrant Transforming Growth Factor-beta (TGFb) signaling drives the pathological microenvironment in glioblastoma (GBM) and amyotrophic lateral sclerosis (ALS). In these central nervous system (CNS) disorders, TGFb promotes fibrotic matrix deposition and maintains an immunosuppressive state. Clinical translation of TGFb-targeting therapies, however, is restricted by distinct limitations: small molecule inhibitors cause systemic toxicity by abolishing homeostatic signaling in peripheral tissues, whereas therapeutic antibodies exhibit poor blood-brain barrier (BBB) penetration. To address this, we developed TbR2-HFt, a human heavy-chain ferritin nanocage displaying TGFb type II receptors. Exploiting transferrin receptor 1-mediated transcytosis, this biologic is designed to cross the BBB and sequester excess pathological ligands. In both BBB-on-chip models and orthotopic GBM mice, TbR2-HFt demonstrated effective BBB permeability, resulting in intracranial accumulation and the downregulation of pSMAD2 levels within the tumor. This target engagement reduced collagen deposition and remodeled the immune microenvironment by depleting regulatory and exhausted T cells. Consequently, TbR2-HFt sensitized tumors to anti-PD-1 blockade, extending survival. These findings demonstrate the efficacy of TbR2-HFt in modulating TGFb-driven pathology within the CNS, suggesting its potential utility for fibrotic neurodegenerative conditions such as ALS.

Supported by National Institutes of Health (R01NS119609)

#### 17. Impact of Antidepressants on HIV Infection Dynamics in Peripheral and Central Myeloid Cells

Modica, AB, BS^1^, Agarwal, Y, BS^2^, Montilla, J, BS^2^, Gaskill, PJ, PhD^2^, Matt, SM, PhD^2^;

^1^Department of Microbiology & Immunology, Drexel University College of Medicine, Philadelphia, PA 191022; ^2^Department of Pharmacology & Physiology, Drexel University College of Medicine, Philadelphia, PA 19102.

Antiretroviral therapy (ART) has extended the lifespan and improved the quality of life for people with HIV (PWH), yet many continue to experience neurocognitive impairment (neuroHIV). Depression is an increasingly common comorbidity that influences HIV disease progression and neuroinflammation. Myeloid cells are primary HIV targets, and their activation has been linked to both HIV persistence and depression-associated inflammation. However, how antidepressant use intersects with infection and ART response remains ill-defined. Given that nearly one-third of PWH have depression, it is critical to understand how antidepressants influence HIV infection in myeloid cells. We hypothesize that antidepressants differentially modulate myeloid activation and HIV infection, with potential implications for ART effectiveness and neuroHIV progression. To test this, donor-matched uninfected and HIV-infected primary human monocyte-derived macrophages (MDMs) were treated with physiological concentrations of antidepressants with distinct mechanisms of action. A subset of infected MDMs also received ART. Infection was assessed by p24 immunofluorescence and p24 quantification in culture supernatants over time. Preliminary analyses suggest donor-dependent differences in HIV infection dynamics associated with antidepressant exposure, with potential implications for ART responsiveness. Future studies will extend these findings to CNS-relevant models by examining antidepressant effects on HIV infection and neuroinflammatory responses in iPSC-derived human brain organoids containing microglia.

Supported by K01 MH132466 and R61/R33 DA058501.

#### 18. In Situ Hybridization Reveals Persistent Viral DNA in The Blood–Brain Barrier Pericytes of HIV Suppressed Individuals

Naranjo, O, PhD, Torices, S, PhD, Schmidlin, S, BS, Tiburcio, D, BS, Gonzalez-Horta, E, PhD, Park, M, PhD, Toborek, M, MD, PhD; Department of Biochemistry and Molecular Biology, University of Miami, Miami, FL 33144.

HIV-1-infected individuals are at higher risk for cerebrovascular and neurological diseases. Despite advances in HIV treatment, around ∼50% of infected individuals experience some degree of HIV-associated neurocognitive impairment (NCI). These pathologies may be driven by low levels of viral replication that persist in HIV-infected brains, leading to immune activation, chronic inflammation, and viral reactivation. We have pioneered research on HIV-1 infection in brain pericytes and previously demonstrated that these cells possess the receptor profile necessary for HIV-1 infection. Blood–brain barrier (BBB) pericytes regulate endothelial tight junctions, capillary permeability, and cerebral blood flow, making them essential for maintaining CNS homeostasis. In vitro studies suggest that BBB pericytes are capable of latent infection and reactivation, and in vivo evidence has demonstrated HIV-1-infected pericytes in human brains with HIV encephalitis. By combining microvessel isolation with highly sensitive spatial RNA in situ hybridization, we achieve cellular-resolution mapping of endothelial cells and pericytes, with codetection of HIV DNA and RNA within microvascular units. Notably, we demonstrate that HIV DNA persists in temporal lobe BBB pericytes isolated from individuals with HIV despite antiretroviral therapy and viral suppression. Given their strategic position and regulatory roles at the neurovascular interface, these findings support the hypothesis that BBB pericytes participate in the formation of HIV brain reservoirs and HIV trafficking into the brain.

Supported by NIH grants (MH072567, MH122235, MH128022, DA059849, DA050528, DA044579, HL126559, and ES036983)

#### 19. DASH TEST: A Rapid Screening Test for Mild Cognitive Impairment

O’Donnell, T, Liang, H, PhD, Shichman, L, MS, Walter, J, MD, PhD, Ernst, T, PhD, Liu, P, PhD, Ryan, A, PhD, Dash, P, MD, Chang, L, MD; Departments of Radiology, Neurology and Medicine, University of Maryland Baltimore School of Medicine, Baltimore, MD 21205.

The prevalence of mild cognitive impairment (MCI) is expected to double by 2050. MCI is a risk factor for dementia, but early detection can maximize effectiveness of treatments and lifestyle interventions. We evaluated the validity of the Dash Dementia Test (DASH) to determine its clinical utility as an MCI screening tool. Methods: 96 participants (52 with MCI and 44 with normal cognition) completed a total of 134 Dash tests and other clinical cognitive assessments, including the Montreal Cognitive Assessment (MoCA) and a battery of neuropsychological tests (NPTs). Discrimination power and convergent validity of DASH test scores were compared to the other clinical assessments using area under the curve (AUC) analyses and correlation matrices, respectively. Intraclass correlation coefficients and Cronbach’s Alpha were calculated to assess internal consistency of DASH results, and a confirmatory factor analysis was conducted to assess structure validity. Results: DASH tests had a higher sensitivity (0.63) but slightly lower specificity (0.87) than MoCA (0.53, 0.89) for detecting MCI. Cronbach’s alpha of DASH tests was 0.64 (95% CI 0.55-0.73), which is moderately reliable. DASH test scores were moderately to highly correlated with MoCA scores and were moderately correlated with scores of cognitive domains within the NPT battery for both MCI and healthy participants. Conclusion: As a quick screening tool, DASH outperforms MoCA (at a fixed cutoff of 26) for detecting MCI. However, across all thresholds, the MoCA has slightly greater discrimination power.

Supported by National Institutes on Aging (3P30AG028747-17S2), National Institute of Neurological Disorders and Stroke (3R01NS115771-03S1).

#### 20. Development and Characterization of a Long-Acting Temsavir Prodrug Formulation

Ogundepo, S, Sultana, A, Silman, B, Ogunnaike, M, Hirbod, K, Le, Nam, HB, Gendelman, H, Edagwa, B. Department of Pharmacology and Experimental Neuroscience, University of Nebraska Medical Center, Omaha, NE 68198.

Human immunodeficiency virus type one (HIV-1) remains a global public health challenge despite the success of antiretroviral therapy (ART) in achieving sustained viral suppression. Long-term virologic control depends on strict regimen adherence, which is often compromised. Temsavir (TMR) remains a critical therapeutic option for heavily treatment-experienced individuals living with multidrug-resistant HIV-1, necessitating innovative long-acting strategies to improve adherence. TMR, the active form of fostemsavir, is a gp120 attachment inhibitor that enables viral suppression in patients with limited treatment options. However, TMR has a short plasma half-life requiring twice-daily oral dosing, contributing to poor adherence, increased pill burden, and risk of drug resistance. Long-acting injectable antiretrovirals have demonstrated the potential to sustain therapeutic drug levels and reduce regimen-related barriers such as stigma, comorbidities, and access to healthcare. With patient adherence challenges in mind, we developed long-acting TMR prodrug formulations using rational prodrug design. Preclinical studies in BALB/c mice and Sprague Dawley rats demonstrated that a lead prodrug nanosuspension, NM1TMR, sustained plasma TMR concentrations above reported oral Ctrough levels for up to three weeks following a single intramuscular injection. NM1TMR exhibited a prolonged apparent half-life consistent with slow absorption and depot-based drug release. These pharmacokinetic findings support further development of NM1TMR as a long-acting therapeutic option for HIV-1 treatment.

Supported by National Institute of Allergy and Infectious Diseases (NIAID)/NIH (R01 AI145542)

#### 21. Comorbid HIV Infection and Methamphetamine Use Differentially Alter Gut-Brain Axis Dysregulation

Pieklo, G, BS, Schindler, K, BS, Baines, K, Brady, S, Toborek, M, MD, PhD; Miller School of Medicine, University of Miami, Miami, FL 33127.

Methamphetamine (METH) use, which has increased to about 33 million people worldwide, elevates the risk for HIV transmission. Both METH use and HIV infection have been linked to major depressive disorder (MDD) via gut-brain axis (GBA) disruption. Despite the high frequency of people living with HIV (PLWH) that use METH, there is a gap of knowledge regarding how this comorbidity leads to development of MDD. In the present study, we hypothesize that METH use prior to HIV infection prompts distinct immune dysfunction by disrupting GBA to initiate MDD by mechanisms unique from either condition alone. To model tolerance driven increased drug usage, subsequent risky behavior and resulting HIV infection, we treated mice with escalating METH doses (1mg/kg- 5mg/kg) three times a day for 6 days prior to ecoHIV infection (1ug). Behavioral tests, including tail suspension and learned helplessness, were employed to assess MDD symptoms. At one week of infection, our results demonstrate a loss of intestinal barrier integrity and increased innate immunity, as well as suppressed epithelial protective and anti-inflammatory signaling. We find that the ileum and colon display varying degrees of barrier integrity loss and inflammatory dysregulation. These data, alongside the ecoHIV+METH group having a phenotype unique to the combined treatment, highlights the complex interaction of this comorbidity on the GBA. Overall, these results suggest that PWLH who misuse METH may require unique therapeutic strategies to alleviate MDD sequela.

Supported by NIDA 5R01DA059849-02.

#### 22. Targeting Sex-Specific Gut Microbiota Improves Social and Cognitive Impairments in EcoHIV-Infected Mice

Yannan, YL, Shinji, SS, Naigang, NL, Ryan, RZ, Matthew, MDS, Manjot, MD, Tessa, TS, Yuto, YH, Meixiang, MH, Hui, HL, Qingsheng, QL, Peter, PAC, David, DV, Xiaolei, XZ; Departments of Psychiatry, Neurology and Behavioral Sciences, Johns Hopkins Drug Discovery, Johns Hopkins University School of Medicine, Baltimore, MD 212872; Department of Neuropsychiatry, Faculty of Medicine, Dentistry, and Pharmaceutical Sciences, Okayama University, Okayama, MD 700-85583; School of Electrical, Computer and Biomedical Engineering, Southern Illinois University, Carbondale, IL 629016; The Wistar Institute, The Wistar Institute, Philadelphia, PA 191047; Department of Medicine, Icahn School of Medicine at Mount Sinai, New York, NY 10029.

(Abstract is not published per author request)

#### 23. HIV and Alcohol-Related Liver Toxicity: The Role of Apoptotic Bodies in Intra-Organ Signaling

Adepoju, LA, MS, Bybee, G, BS, Pathania, AS, PhD, Osna, NA, MD, PhD; Department of Pharmacology and Experimental Neuroscience, University of Nebraska Medical Center, Omaha, NE, 68198 United States.

Liver disease is a major cause of morbidity among people living with HIV (PLWH), particularly in those who abuse alcohol. Although HIV infection and ethanol metabolism independently induce hepatocyte injury, the synergistic effects driving liver damage remain poorly defined. Apoptotic bodies (ABs) from injured hepatocytes may propagate intra-organ toxicity by transferring inflammatory and death signals to naive cells. We hypothesized that ABs from HIV-1 ADA and alcohol-exposed hepatocytes enhance hepatotoxicity by inducing various cell death, while activating inflammatory signaling. Huh7.5-CYP2E1 hepatocytes were exposed to HIV-1 ADA and an acetaldehyde-generating system (AGS) to model alcohol metabolism. UV-induced apoptosis was used to generate ABs, which were characterized. Naïve hepatocytes were incubated with ABs for 2–60 hours. Cell death pathways and inflammatory responses were assessed using pathway-specific markers, and inflammasome gene expression, ROS and LDH release. Mechanistic specificity was confirmed using inhibitors and an ASGPR-blocking peptide. ABs derived from dual insult hepatocytes induced apoptosis, and others, with increased oxidative stress, necrosis, and upregulation of IL-6, TNF-α, NLRP3, IL-1β, and IL-18. Inhibitors reduced pathway activation by 50–70%, while ASGPR blockade markedly attenuated AB-induced injury. These findings show that HIV and alcohol synergistically exacerbate liver injury through AB-mediated intra-organ signaling, identifying AB formation and uptake as potential therapeutic targets in alcohol-induced liver disease in PLWH.

Supported by National Institute on Alcohol Abuse and Alcoholism (NIAAA)

#### 24. SIV/HIV-Induced Lipid Dysregulation Induce Neuroinflammation and Tissue Damage, and The Role Of Pannexin-1 in Neuro-HIV.

Ajasin, DA, PhD, Devin, SD, BS, Prideaux, BD, PhD, Eugenin, EE, PhD, Gonzalez-Garcia, HG, MD; Department of Internal Medicine-Infectious Disease Division, University of Texas Medical Branch, Galveston, TX, 77555 United States. ^2^John Sealy School of Medicine, University of Texas Medical Branch, Galveston, TX, 77555 United States. ^3^Department of Neurobiology, University of Texas Medical Branch, Galveston, TX, 77555 United States.

HIV-associated cardiovascular disease (HIV-CVD) and HIV associated neurocognitive disorder (HAND) are two of the most common HIV comorbidities seen in people living with HIV (PLWH), which are associated with chronic inflammation despite low viremia in PLWH. To understand how inflammation in the absence of viremia contributes to these comorbidities, we performed spatial unbiased analyses of lipids from post-mortem heart and brain tissues from both HIV-infected individuals and SIV-infected non-human primates (NHPs). Using mass spectrometry imaging (MSI), confocal imaging and immunoblotting, we identified arachidonic acid (AA) as one of the major lipids that changed significantly between the uninfected and infected tissues, which is associated with significant elevation in the levels of critical enzymes in arachidonic acid catabolic pathways, and inflammation. Interestingly, inhibition of an hemichannel (HC), Pannexin-1 (Panx-1) in the NHP led to significant reduction in the level of arachidonic acid and the expression of critical enzymes in the same pathways. While inhibition of Panx-1 HC does not reduce all inflammation, here we observed that arachidonic acid-associated inflammation is dampened in both the heart and the brain of both human and NHP samples. This result suggests that inhibition of Panx-1 HC could be therapeutic and ameliorate HIV-CVD and HAND.

#### 25. Dopamine Drives Temporal Regulation of Inflammatory Pathways in Human Monocyte-Derived Macrophages

Curley, E.O., Daniali, M, Channer, B, PhD, Nolan, R, PhD, Kist, T, Gaskill, P.J., PhD; Department of Pharmacology and Physiology, Drexel University, Philadelphia, PA, 19102 United States.

Dopamine (DA) is traditionally associated with the CNS, but growing evidence also indicates a key role in peripheral innate immunity. The Gaskill lab previously showed that DA amplifies inflammation in human monocyte-derived macrophages (hMDMs) by driving nuclear factor kappa-B (NF-κB) translocation, activating the NLRP3 inflammasome, and inducing production of inflammatory cytokines such as IL-6, IL-1β, and CXCL-10. However, the signaling pathways by which extracellular DA induces this response remain opaque. Our prior data suggest that DA acts via a non-canonical pathway unrelated to cAMP signaling, which is canonically associated with DA signaling. Therefore, we examined possible signaling pathways before and after 1hr, the time at which DA induces NF-κB nuclear translocation. hMDMs were treated with DA (1uM), IL-6 (20ng/mL), or LPS (10ng/mL) for 5, 15, 30, or 60 min, then assessed by Western blot for activation of JAK/STAT and AKT pathways. hMDMs were also treated with DA (1uM) or LPS (10ng/mL) for 5hr and then BzATP (200uM) for 1h to activate the NLRP3 inflammasome, assessing protein samples by Western for Gasdermin (GSDM) protein cleavage, a marker of NRLP3 activity. Our results suggests that DA does not activate AKT, but increases STAT activity before NF-κB activation, while driving GSDM-D and B cleavage and noncanonical NLRP3 activation after NF-κB activation. Identifying pathways by which DA modulates macrophage inflammatory responses is critical for defining its immunomodulatory role and finding therapeutic targets for diseases with altered dopamine levels.

Supported by NIDA R33DA058501

#### 26. Osteopontin Modulates Sex-Specific Viral, Immunological, and Metabolic Responses to Antiretroviral Therapy in Humanized Mice

Davis, ED, PhD, Wangui, JW, Argandona Lopez, CAL, Shirmamedova, AS; Department of Neurology, Johns Hopkins University School of Medicine, Baltimore, MD, 21287 United States.

People with HIV (PWH) now experience lifespans comparable to uninfected individuals due to the high efficacy and availability of antiretroviral therapy (ART). Risk of comorbidities including neurological and metabolic disorders increases as PWH age and can be further exacerbated by chronic inflammation. Moreover, weight gain following ART initiation is frequently observed, indicating that ART regimens, in combination with viral and host-specific factors, influence long-term clinical outcomes in PWH. The mechanisms governing this interplay between ART, inflammation, and metabolism remain poorly defined. Here, we explore osteopontin (OPN), a neuroinflammatory protein upregulated during HIV infection, as a potential mediator of ART-linked weight change and viral suppression. Humanized HIV-infected mice were treated for 5 months with OPN-suppressing RNA aptamers and supplied with either ART-laced or control chow. Blood was collected longitudinally to assess HIV RNA levels and immune profiles. Body weight was also measured. Intriguingly, HIV RNA was increased in OPN- males but decreased in females when compared to OPN+ counterparts. In HIV-infected females, ART negatively impacted food intake in an OPN-dependent manner. Despite reduced food consumption, these mice exhibited an 8% weight increase while on ART. Sex, OPN, and ART contributed to percentages of circulating B cells, T cells, and myeloid cells. These findings highlight complex, sex-dependent interactions between OPN and ART-associated weight gain, providing a novel target for improving ART-based interventions in PWH.

Supported by NINDS R01NS102006

#### 27. Understanding Differences in CRISPR Repair Pathways, Profiles, and Outcomes in HIV-1 Target Cells

Effah, S, MS, Dampier, W, PhD, Nonnemacher, M, PhD, Wigdahl, B, PhD; Microbiology and Immmunology, Drexel University, Philadelphia, PA, 19104 United States.

CRISPR gene editing of the integrated HIV-1 proviral DNA creates double-strand breaks within the provirus which are repaired by the host’s double-stranded break (DSB) repair pathways. The repair of these breaks can introduce indels (insertions and deletions) that impair transcription of the integrated HIV-1 proviral DNA. Though these repair proteins are known to be involved in CRISPR-mediated HIV-1 proviral DNA editing, less is known about their expression in cells targeted by HIV-1 under resting and activated conditions. Ultimately, what these protein expression dynamics mean for CRISPR editing efficiency in these HIV-1 targeted cells also remains unknown. We initially assessed total cellular expression of nonhomologous end-joining (NHEJ) associated repair proteins Ku70, LIG4 and DNA-PKcs in a CD4 T cell, macrophage and microglia cell models. Western immunoblot densitometry analysis showed that on a cellular level, DNA-PKcs expression was significantly higher in microglia than in CD4 T cells. DNA-PKcs expression was also higher in activated CD4 T cells compared to resting CD4 T cells. Finally, LIG4 expression was also significantly higher in CD4 T cells compared to microglia. Recently with our distinct amplification of the 5’ and 3’ LTR regions of the provirus from our latently infected cell models (J-Lat 10.6 and HC69), our goal is to assess repair protein binding at the CRISPR-Cas9 cut site and assess how modulation of repair protein activity can impact CRISPR-Cas9-mediated repair profiles and eventually CRISPR-Cas9 editing efficiency in cells targeted by HIV-1.

#### 28. Cocaine Overdose Induces Gap Junctional Compromise in the Heart, Resulting in Death

Espinoza-Perez, C, PhD, Luu, R, PhD, Valdebenito-Silva, S, PhD, Eugenin, E, PhD; Department of Neurobiology, University of Texas Medical Branch (UTMB), Galveston, TX, 77550 United States.

Background. Cocaine overdose is a major public health concern and is strongly associated with acute cardiovascular complication, but the underlying mechanisms remain poorly understood. Gap junctions formed by connexin 43 (Cx43) are essential for cardiac electrical conduction and coordinated myocardial contraction, and their disruption can promote life-threatening arrhythmias. Here, we investigated whether lethal cocaine doses directly target the heart, with a particular focus on gap junctions and myocardial contractility. Methods. C57BL/6 mice were intraperitoneally injected with a lethal dose of cocaine (90 mg/kg). Mice began to show signs of overdose within 5 minutes of injection. Cardiac tissue was collected to evaluate gap junctional communication, given the essential role of connexins in cardiac electrical conduction. We performed functional assays (dye coupling), assessed Cx43 distribution by confocal microscopy, and evaluated myocardial structural integrity. Results. Cocaine overdose strongly compromised gap junctional communication, with marked alterations in Cx43 localization at intercalated discs and increased lateralization. These changes were associated with hypercontracted myocardial muscle with features compatible with myofibrillar damage. Conclusions. Lethal cocaine overdose induces maladaptive Cx43 redistribution and loss of effective gap junctional communication, together with tetanic-like myocardial hypercontraction. These effects provide a plausible mechanistic basis for its pro-arrhythmogenic cardiac toxicity.

Supported by National Institute of Mental Health (NIMH), grants MH128082 and MH134761

#### 29. Feature-Graph–Powered Multi-Omics Integration Reveals Interpretable Biomarkers for Alzheimer’s Disease

Foroughi Nezhad, AFN, MS, Pendyala, GP, PhD; Department of Anesthesiology, College of Medicine, University of Nebraska Medical Center, Omaha, NE, 68198 United States.

The rapid expansion of multi-omics data (e.g., transcriptomics and proteomics) alongside growing biological prior knowledge creates a strong need for supervised methods that integrate heterogeneous, high-dimensional modalities for disease prediction and biomarker discovery. However, integration remains challenging due to small sample sizes relative to feature space, imbalanced modalities, missing data, and limited use of known feature–feature biology (e.g., pathways and interaction networks), which reduces interpretability and functional relevance. We hypothesize that biologically informed feature graphs in explainable GNNs enhance Alzheimer’s prediction and reveal interpretable biomarkers beyond existing unimodal and multi-omics methods. Our proposed framework (GNNRAI) uses modality-specific GNNs over biologically informed feature graphs to learn embeddings, which are aligned and integrated via a set transformer. Unimodal heads enable robust prediction under missing modalities. Across 16 biodomains, GNNRAI outperformed MOGONET and unimodal models in AD/control accuracy. Proteomics was more predictive than transcriptomics, while integration balanced predictive power with sample coverage. Explainability recovered known AD markers (APP, APOE, GFAP) and novel candidates (e.g IQGAP3), with lipid metabolism emerging as a key cross-domain hub. GNNRAI improved AD classification and identify interpretable biomarkers, even with incomplete data. These biomarkers enable improved diagnosis and targeted drug discovery and repurposing, motivating our ongoing deep learning repurposing.

Supported by The Kinman-Oldfield Family Foundation, Nancy and Ronald Reagan Alzheimer’s Award at UNMC

#### 30. Buprenorphine Reduces Transmigration of CD14+CD16+ Mature Monocytes Across the BBB: Implications for HIV-NCI

Gausepohl, AM, MS^1^, Murphy, A, PhD^1^, Lopez, L, BS^1^, Hernandez, CA, PhD^1^, Atkins, S, MS^1^, Kelschenbach, J, PhD^2^, Volsky, DJ, PhD^3^, Berman, JW, PhD^1^; ^1^Department of Pathology, Albert Einstein College of Medicine, Bronx, NY, 10461 United States. ^2^Department of Pharmacology & Physiology, Drexel University College of Medicine, Philadelphia, PA, 19102 United States. ^3^Department of Medicine, Icahn School of Medicine at Mount Sinai, New York, NY, 10029 United States.

HIV-associated neurocognitive impairment (HIV-NCI) affects up to 50% of people with HIV (PWH) despite antiretroviral therapy (ART). There are no treatments for HIV-NCI for PWH on ART. HIV-NCI is mediated in part by chemokine-induced transmigration of CD14+CD16+ mature monocytes across the blood brain barrier (BBB), bringing virus into the CNS and contributing to neuroinflammation. Opioid use disorder exacerbates HIV-NCI. We previously showed in an EcoHIV-infected mouse model of HIV-NCI that buprenorphine, an opioid agonist therapy, reverses cognitive impairment that occurs with infection. This was associated with decreased monocyte entry and HIV DNA within the brains of infected mice. We are now examining the impact of buprenorphine on chemokine-mediated migration of primary human mature monocytes across a human BBB model. Buprenorphine treatment of uninfected mature monocytes reduces their transmigration to CCL2 (1.3-fold) and transmigration of HIV-infected cells to both CCL2 and CXCL12 (1.4 and 1.2-fold, respectively). We are determining what steps buprenorphine limits in the transmigration of these cells across the BBB. Buprenorphine reduces CCL2 and CXCL12-mediated adhesion of HIV-infected mature monocytes to ICAM-1 (1.5 and 1.2-fold, respectively) and VCAM-1 (1.3 and 1.4-fold, respectively), proteins on the BBB endothelium that mediate firm arrest of these cells early in transmigration. Our studies show buprenorphine reduces transmigration of mature monocytes across the BBB, a key HIV neuropathogenic mechanism. We propose buprenorphine may be a therapy for HIV-NCI.

Supported by NIH/NIDA R01DA041931; NIH/NIAID T32AI007501; NIH/NIAID P30AI124414

#### 31. Rewiring of Pericyte Immune and Vascular Functions by Phthalates in HIV Infection

Gonzalez-Horta, E, PhD, Haden, H, BS, Kush, K, BS, Schmidlin, S, BS, Tiburcio, D, BS, Naranjo, O, PhD, Torices, S, PhD, Toborek, M, MD, PhD; Department of Biochemistry and Molecular Biology, Miller School of Medicine/University of Miami, Miami, FL, 33136 United States.

Blood–brain barrier (BBB) dysfunction contributes to HIV-associated neurocognitive disorders, and environmental toxicants may worsen neuroinflammation. Brain vascular pericytes (BVP), critical for BBB stability and microvascular health, are recognized as targets of HIV infection. However, the impact of phthalates, ubiquitous environmental contaminants, on BVP function during HIV infection remains poorly understood. We investigated how acute and chronic exposure to environmentally relevant phthalates affects inflammatory signaling, viral replication, and BBB-supportive function of BVP during HIV infection. Phthalates reprogrammed HIV-infected BVP immune function, shifting inflammasome sensing, with increased NLRP1 and AIM2 expression and decreased NLRP3 and NLRC4 expression. Exposure to phthalates also increased IL-1β, IL-18, and IL-10 mRNA levels but suppressed IL-6 mRNA regardless of HIV status. Phthalates induced early angiogenic suppression (VEGFA, IL-6) that transitioned to structural remodeling (MMP2, upregulated; ANGPT, downregulated) under chronic exposure, a profile consistent with matrix remodeling and potential barrier weakening. Moreover, chronic exposure to phthalates enhanced HIV replication. Our research identifies phthalates as underappreciated modulators of neuroinflammation and HIV neuropathogenesis. Phthalate exposure altered BVP functions and increased HIV replication, potentially worsening BBB-associated neuroinflammation. These findings highlight the importance of including environmental factors when evaluating neuroimmune interactions and BBB integrity.

Supported by in part by NIH grants ES036983, MH128022, and MH072567

#### 32. Informing an HIV-1 CRISPR Cure Strategy through Chromatin Accessibility Mapping and Epigenetic Alteration

Gunderson, CE, MS, Jones, J, MS, Klopfenstein, DV, PhD, Dampier, W, PhD, Nonnemacher, MR, PhD, Wigdahl, BD, PhD; Department of Microbiology and Immunology, Drexel University College of Medicine, Philadelphia, PA, 19104 United States.

Despite advances in antiretroviral therapeutics, human immunodeficiency virus type 1 (HIV-1) persists via the latent viral reservoir as an integrated provirus. Utilization of CRISPR/Cas9 gene editing to excise or inactivate the integrated provirus has been proposed as a curative strategy. We have found that guide RNAs (gRNA) that target two sites critical for latency maintenance and reactivation in the 5’ long terminal repeat (LTR) – an NF-kB binding site and the transactivation response element (TAR) – were the most effective in reducing HIV-1 proviral reactivation in vitro. As studies have shown that epigenetic interference can impede CRISPR/Cas cleavage of target DNA, we are interested in further investigating chromatin accessibility across the length of the integrated provirus. Therefore, we have analyzed publicly available HIV-1 ATAC-seq datasets from both lymphoid and myeloid cells to assess how different conditions impact the chromatin state of the provirus. These results show accessibility varies by cell type and activation type. Combining this information with our own ATAC-seq, ChIP-seq, and methyl-seq data will allow us to further elucidate differences in the epigenetic control of HIV-1 latency and reactivation in diverse cell types, and to explore whether the use of latency reversal agents at subtherapeutic doses may be integrated with CRISPR/Cas delivery to alleviate epigenetic repression at targets sites while enhancing editing efficiency.

Supported by NIMH R01 MH110360, NIMH P30 MH092177, NIMH T32 MH079785

#### 33. Methamphetamine Increases Uninfected And HIV-Infected Mature Monocyte Adhesion, Chemotaxis, and Blood-Brain Barrier Transmigration in Response to CCL2 and CXCL12

Hernandez, CA, PhD, Chilunda, V, PhD, Lopez, L, Calderon, T, PhD, Berman, JW, PhD; Department of Pathology, Albert Einstein College of Medicine, Bronx, NY, 10461 United States.

40.8 million people are living with HIV (PWH). Antiretroviral therapy (ART) increases the lifespan and quality of life of PWH. Despite viral suppression with ART, 15-50% of PWH develop HIV- neurocognitive impairment (HIV-NCI). 13-30% of PWH use methamphetamine (MA) that exacerbates HIV-NCI. HIV-NCI is mediated in part by mature monocyte (CD14+CD16+) transmigration across the blood-brain barrier (BBB) and entry into the CNS, resulting in neuroinflammation. We showed that mature monocytes preferentially transmigrate across a model of the human BBB to CCL2 or CXCL12, chemokines increased in the CNS of PWH. Here, we characterize effects of MA on steps of transmigration. We demonstrate that MA increases adhesion of uninfected mature monocytes treated with CCL2 to ICAM-1, important to this step in transmigration. The average fold change to baseline for CCL2 is 2.3, compared to MA+CCL2 is 2.9. MA also increases chemotaxis of uninfected and HIV-infected mature monocytes to CCL2 or CXCL12 compared to baseline. The fold change for MA is 1.0, CCL2 is 1.3, CXCL12 is 2.7, MA+CCL2 is 1.6, and MA+CXCL12 is 3.0 for uninfected mature monocytes. Using infected cells MA is 1.2-fold change, CCL2 is 1.0, CXCL12 is 2.6, MA+CCL2 is 1.4, and MA+CXCL12 is 3.1. MA increases BBB transmigration of HIV-infected mature monocytes to CCL2 or CXCL12, fold change to baseline to MA is 1.3, CCL2 is 1.4, CXCL12 is 1.5, MA+CCL2 is 2.5, and MA+CXCL12 is 3.1. Thus, MA may contribute to HIV-NCI by increasing monocyte entry into the CNS, resulting in exacerbated neuroinflammation and reseeding of viral reservoirs.

Supported by National Institute on Drug Abuse

#### 34. A Scalable Ultra-Long-Acting Pediatric Bictegravir Prodrug Formulation

Hirbod, Kimia, MS^2^, Raut, Samiksha S, MS^1^, Sillman, Brady, PhD^1^, Le, Nam Thai Hoang, BS^1^, Ogundepo, Sunday O, BS^1^, Hanson, Brandon, MS^1^, Gendelman, Howard E, MD^1^, Edagwa, Benson, PhD^1^; ^1^Department of Pharmacology & Experimental Neuroscience, University of Nebraska Medical Center, Omaha, NE, 68198 United States. ^2^Department of Pharmaceutical Sciences, University of Nebraska Medical Center, Omaha, NE, 68198 United States.

Simplification of HIV treatment over the past decade, including once-daily single-tablet regimens and long-acting injectable antiretroviral therapies (ART), has improved patient satisfaction and outcomes. However, significant treatment gaps persist in children living with HIV-1 infection, where daily oral ART remains limited by adherence challenges. Long-acting ART would be particularly beneficial for pediatric patients for whom achieving high rates of daily adherence and virologic suppression remains challenging. To these ends, an ultra-long-acting homodimeric prodrug nanosuspension of bictegravir (NM8BIC) was identified from a prodrug library and evaluated for pharmacokinetic profile in juvenile rats. Bictegravir (BIC) is a guideline-preferred potent integrase-strand transfer inhibitor for the treatment of HIV infection in pediatric patients. Scalable aqueous NM8BIC nanosuspensions were developed and evaluated for antiviral efficacy and pharmacokinetics. In vitro studies in monocyte-derived macrophages demonstrated enhanced intracellular uptake, retention, and prolonged HIV-1 suppression compared with native BIC. In vivo pharmacokinetic evaluation in juvenile Sprague Dawley rats following a single subcutaneous dose (50 mg/kg) showed sustained plasma BIC concentrations above 4x protein-adjusted IC95 for over one month, with good tolerability and no adverse effects observed. These findings support further development of NM8BIC as a promising LA therapeutic option for improving treatment adherence and virologic outcomes in pediatric HIV infection.

Supported by 5R61AI188553

#### 35. Investigating The Synergism of HIV and Opioid Use on Neuroinflammation and Cognitive Impairment

Hong, D, MS^1^, Jiang, G, PhD^3^, Chaillon, A, MD, PhD^2^, Fields, JA, PhD^1^; ^1^Psychiatry, UC San Diego, La Jolla, CA, 92093 United States. ^2^Infectious Diseases, UC San Diego, La Jolla, CA, 92093 United States. ^3^Biochemistry & Biophysics, UNC Chapel Hill, Chapel Hill, NC, 27599 United States.

Antiretroviral therapy (ART) has extended the lifespan of people with HIV (PWH), yet neurocognitive impairment (HIV-NCI) affects 30-50% of PWH. PWH are also prescribed opioids at higher rates and doses than the general population, increasing the risk of opioid use disorder. HIV and opioids may interact to promote chronic inflammation, which is associated with HIV-NCI, but the underlying mechanisms remain unclear. We hypothesized that HIV and opioids synergistically induce mitochondrial injury, leading to activation of the NLRP3 inflammasome. This hypothesis was evaluated using brain myeloid cells (BrMCs) isolated from freshly autopsied brain tissue and flash-frozen brain tissue from Last Gift donors. NLRP3 activation was assessed by real-time PCR and immunoblotting. In BrMCs, co-exposure to GDF-15 and lipopolysaccharide (LPS) increased mRNA expression of NLRP3 components, whereas individual exposures had no effect. In contrast, immunoblot results showed that LPS did not alter NLRP3 pathway protein expression, while GDF-15 alone increased protein levels, with no effect following co-exposure. Additionally, postmortem brain tissue from PWH with opioid use showed reduced NLRP3 pathway protein expression. These findings suggest that NLRP3 inflammasome activation may be BrMC-specific or that alternative inflammasome pathways or mechanisms contribute to inflammation in other CNS cell types. Future studies using BrMCs and brain tissues from a larger number of decedents will help clarify the role of NLRP3 in HIV- and opioid-induced neuroinflammation and neurotoxicity.

Supported by NIDA/DA062278

#### 36. Characterizing The Effects of Chromatin Structures on CRISPR/Cas9-Induced Edits of The HIV-1 Long Terminal Repeat

Jones, JEJ, MS, Gunderson, CEG, MS, Berman, REB, BS, Dampier, WND, PhD, Nonnemacher, MRN, PhD, Wigdahl, BW, PhD; Department of Microbiology and Immunology, Drexel University College of Medicine, Philadelphia, PA, 19102 United States.

Human immunodeficiency virus type 1 (HIV-1) is a retrovirus that integrates into the host cell’s genome as a provirus, establishing a reservoir of latently-infected cells. This presents a major therapeutic challenge, as infected cells avoid immune clearance and current antiretroviral drugs cannot target this stage of the replication cycle. Therefore, there is an outstanding need for the development of cure strategies that can clear or inactivate the provirus. One cure strategy is CRISPR/Cas9 gene therapy, whereby the Cas9 endonuclease complexes with a target DNA region through a complementary guide RNA (gRNA) and induces a double-stranded break (DSB) that is repaired by the cell. DSB repair can induce insertions, deletions, and other edits. gRNAs targeting the long terminal repeat (LTR) sequence can disrupt proviral gene expression by introducing edits to transcription factor binding sites, the transcription initiation and termination sequences, and/or the viral transactivation response element (TAR). In a J-Lat 10.6 system, approximately half of the proviruses were inactivated following treatment with gRNAs targeting the DNase hypersensitive NF-κB binding sites (κB1) or the nucleosome-protected TAR sequence (SMRT-01). Sequencing of the LTRs post-CRISPR revealed that the editing efficiency of κB1 was significantly higher than that of SMRT-01, supporting the notion that Cas9 preferentially cleaves in regions of open chromatin. Therefore, we propose to use small molecules to remodel the nucleosomes on the 5’ LTR to promote CRISPR/Cas9 inactivation of proviral gene expression.

#### 37. Can Antiretroviral Drugs Affect HIV and Alzheimer’s Disease Pathologies?

Kumar, MK, MS, Du, XD, MS, Zhang, CZ, MS, Foster, EGF, BS, Bhattarai, SB, MS, Yeapuri, PY, PhD, Kadry, RK, MS, Gorantla, SG, PhD, Mosley, RLM, PhD, Sil, SS, PhD, Gendelman, HEG, MD; University of Nebraska Medical Center, Omaha, NE, 68198 United States.

Antiretroviral therapy (ART) suppresses HIV and reduces the risk of profound dementia in people with HIV, but mild neurocognitive impairment often persists. Evidence suggests nucleoside reverse transcriptase inhibitors (NRTIs) may protect against Alzheimer’s disease (AD), yet their mechanism in HIV/AD remains unclear. To address this, we developed a humanized NOGAPPKM670,671N/IL-34 (NAIL) mouse model carrying human APPKM670,671NL Swedish mutation on immunodeficient NOG/IL-34 (NOD.Cg-PrkdcscidIl2rgtm1SugTg(CMV-IL-34)1/Jic) background. Reconstituted NAIL mice were infected at 6 months of age with HIV-1ADA. Following productive viral infection, animals were treated with combinations of emtricitabine, tenofovir, and dolutegravir via food for four weeks. Notably, ART reduced plasma HIV-1 RNA and restored CD4+ and CD8+ T-cell counts. Compared with NOG/IL controls, both uninfected NAIL and ART-treated HIV-1-infected NAIL mice showed significantly elevated soluble and insoluble Aβ, increased Aβ deposition, microgliosis, and neuronal loss in the olfactory bulb, cortex, and hippocampus. Within the NAIL model, ART-treated HIV-1-infected mice displayed no changes in cortical and hippocampal Aβ relative to uninfected NAIL control but exhibited significantly greater Aβ accumulation in the olfactory bulb. Immunofluorescence further revealed marked microglial activation and Map2+ neuronal loss in the olfactory bulb and hippocampus of ART-treated HIV-infected NAIL mice relative to the uninfected NAIL group. These findings highlight the complex interactions between HIV-1, ART, and AD.

Supported by NIH Grants R01 AG043540, 2R01 NS034239, and Carol Swarts, MD Emerging Neuroscience Research Laboratory

#### 38. Cocaine Increases HIV Pathogenesis by A Dopamine-Independent Mechanism

Luu, R, Eugenin, E, PhD; Dept. of Neurobiology, University of Texas Medical Branch at Galveston, Galveston, TX, 77555 United States.

Cocaine is the second most abused recreational drug. In addition to overdoses, cocaine is associated with inflammation, immunological suppression, and worsened infectious diseases, including HIV. Within the brain, cocaine causes both acute and chronic imbalance of neurobiochemistry by inducing chronically elevated neurotransmitters such as dopamine and preventing its reuptake at the neuronal cleft. However, in the immune system, cocaine suppresses cell function and promotes a pro-inflammatory state. Elevated levels of extracellular dopamine in response to cocaine increased susceptibility to HIV viral entry and neuroinflammation but the mechanism of cocaine-inducing immune/HIV dysregulation is poorly explored. We hypothesize that cocaine alone induces a transcriptionally active state within peripherally circulating immune cells that makes them susceptible to HIV infection and higher viral replication by a dopamine-independent mechanism. Here, we demonstrate that circulating levels of catecholamines in healthy, HIV, HIV+ART, and drug users are minimal and not correlated with variables such as drug status, immune compromise, or replication, suggesting an alternative mechanism of immune activation. Treatment of HIV-infected PBMCs with cocaine enhanced viral replication by an independent dopaminergic mechanism because blocking DAT or D1/D2 receptors on immune cells did not alter the enhanced replication elicited by cocaine. Therefore, cocaine is able to induce viral replication in a manner independent of soluble dopamine or its receptors through process distinct from the CNS.

Supported by The National Institute of Mental Health grant, MH128082/MH134761, the National Institute of Neurological Disorders

#### 39. Development of an Ultra-Long-Acting Doravirine Prodrug Nanoformulation

Mancy, AS, Hanson, BW, Gowda, B, Sillman, B, Thai Hoang Le, N, Gendelman, HE, Edagwa, B; Department of Pharmacology and Experimental Neuroscience, University of Nebraska Medical Center, Omaha, NE, 68198-5000 United States.

Long-acting antiretroviral therapy formulations represent a significant advance in HIV therapeutics. However, available non-nucleoside reverse transcriptase inhibitors rilpivirine and efavirenz have developed resistance and CNS side effects, respectively, unlike doravirine (DOR). The present study was designed to transform DOR into stable lipophilic prodrug nanosuspension to improve the Pharmacokinetic (PK) profiles in BALB/cJ mice and Sprague Dawley rats (SD). The synthesized prodrug (IM2DOR) exhibited higher lipophilicity evaluated by measuring the solubility in octanol compared to DOR. The nanoformulated DOR (NDOR) and IM2DOR transformed into aqueous nanosuspensions using high-pressure homogenization process and screened for PK profiles in BALB/cJ mice following a single intramuscular (IM) injection at a dose of 90 mg DOR equivalents/kg, IM2DOR sustained plasma DOR concentrations above 4x PA-IC95 for 4 months compared to NDOR that depleted after 2 months. Using the same dose, IM2DOR administered into SD rats as a single subcutaneous (SC) injection provided sustained plasma drug levels above 4x PA-IC95 for more than 3 months compared to IM levels that sustained for 3 months. IM2DOR nanosuspension improved intracellular drug delivery and antiretroviral activity in primary human monocyte-derived macrophages used for cellular uptake and retention, antiretroviral efficacy, and cytotoxicity compared to NDOR. These results suggest that a single IM or SC injection of IM2DOR sustains plasma DOR longevity above 4x PA-IC95 for at least 3 months in BALB/cJ mice and SD rats.

#### 40. Developing Broadly Neutralizing Antibody-Producing Glial Progenitors to Eradicate Human Immunodeficiency Virus from the Brain

McDougall, L, MS^1^, Abassi, A, PhD^2^, Sajadi, M, PhD^2^, Heredia, A, PhD^2^, Chang, L, MD^1^, Liang, Y, PhD^1^, Chu, C, PhD^1^, Munawwar, A, PhD^2^, Kalkowski, L, PhD^1^, Fadon-Padilla, L, PhD^1^, Laciuga, A^1^, Atkinson, B, PhD^2^, Janowski, M, MD, PhD^1^, Walczak, P, MD, PhD^1^; ^1^School of Medicine; ^2^Institute of Human Virology, University of Maryland, Baltimore, Baltimore, MD, 21201 United States.

While antiretroviral therapies (ART) are effective in suppressing systemic HIV replication, clearance of viral reservoirs in the brain remains limited by restricted drug penetration across the blood-brain barrier. Additionally, HIV reservoirs cause neurodegeneration and demand long-term ART to suppress viral resurgence, resulting in further neurotoxicity. Therefore, exploring new therapeutic strategies to eliminate HIV from the brain is necessary. This study utilizes glial progenitor cells (GRPs), with white-matter repair properties, by enhancing therapeutic capacity through genetic engineering. Our modified GRPs produce two broadly neutralizing antibodies (bNAb), with differing heavy and light chain linkers, which bind HIV gp120 for viral clearance. Stable integration of the bNAb constructs was verified by GFP reporter and bNAb co-expression in up to 90% of the CII-bNAb-mGRPs and CIII-bNAb-mGRPs via flow cytometry. Functionality was measured by neutralization assay of the purified bNAbs, with a TCID50 at 1.2ug/ml of CII-bNAb and 0.4mg/ml of CIII-bNAb. Binding assays to gp120 on the RAJI B-lymphocyte cell surface identified 90% of the cells had bound CII-bNAb and CIII-bNAb. In addition, intracerebral implantation of the GRP and RAJI cells found ∼50% of RAJI’s bound CII-bNAb and CIII-bNAb. Finally, mGRPs delivered interarterially were distributed globally throughout the brain and expressed GFP and bNAb up to seven days post-injection. Ongoing experiments will employ this strategy for viral reservoir clearance in the brains of IL-34-NOG humanized mice.

Supported by NIDA/NIH (R01DA056739; MPI: Walczak, Heredia)

#### 41. 17α-Estradiol Protects Against HIV-1 Tat-Induced Cellular Senescence in Human Astrocytes

Miteu, G, MS, Datta, G, PhD, Hasler, W, PhD, Chen, X, PhD; Department of Biomedical Science, University of North Dakota, Grand Forks, ND, 58203 United States.

Tat continues to contribute to the development of HIV-associated neurocognitive disorders (HAND) in ART era. As a secreted protein, Tat enters cells via receptor-mediated endocytosis. We have shown that Tat induces endolysosome dysfunction in CNS cells, and importantly, Tat induces cellular senescence via its endolysosome damaging effects. There exist sex disparities in HAND with women having greater neurocognitive impairments. Such sex-differences could not be readily explained by the protective role of 17β-estradiol (E2), which is higher in women. But the precursor of 17α-E2, an isoform of 17β-E2, is twice as high in men. Thus, 17α-E2, the predominant E2 in the brain may contribute to such sex-differences. Significantly, 17α-E2 exerts endolysosome enhancing effects and neuroprotective effects. Here, we explored the role of 17α-E2 in protecting against Tat-induced senescence in primary human astrocytes. Our findings reveal that pre-treatment with 17α-E2 significantly protects against Tat-induced increases in senescent markers (SA-β-gal activity, p16, and p21) and the release of senescence associated secretory phenotype (SASP) factors (IL-6, IL-8, and MCP1/CCL2). Additionally, we show that pre-treatment with 17α-E2 significantly protects against Tat-induced endolysosome dysfunction, as evidence by increased release of galectin-3 and cathepsin D. Our findings suggest that 17α-E2 protects against Tat-induced senescence. Such findings provide insights into the sex differences in HAND and may lead to the development of novel therapeutic strategies.

Supported by MH134592 and DA05928

#### 42. EcoHIV Infection Impairs Extinction Learning and Dysregulates Corticostriatal Microglia

Namba, MD, PhD, Yadlapalli, A, Wiafe-Jackson, J, Goldberg, SL, MS, Jackson, JG, PhD, Barker, JM, PhD; Department of Pharmacology & Physiology, Drexel University College of Medicine, Philadelphia, PA, 19102 United States.

Human immunodeficiency virus (HIV) remains a persistent public health dilemma worldwide that is associated with neurocognitive impairment (NCI). Many preclinical studies suggest that HIV can dysregulate reward learning, which may contribute to the common co-occurrence of substance use disorders with HIV. Nevertheless, very few studies have interrogated what specific reward learning constructs are impacted by chronic HIV infection, and elucidating these neurobehavioral processes is crucial to identifying effective treatment targets for treating NCI in the context of HIV. Here, we examined the impact of chronic HIV infection on sucrose-seeking behavior using the EcoHIV rodent model in male and female mice. EcoHIV had no impact on sucrose self-administration but significantly impaired extinction learning. EcoHIV also significantly reduced responding for sucrose among females but not males in a progressive ratio test. Examination of corticostriatal microglia via Iba1 immunolabeling revealed a significant reduction in Iba1+ cells within the infralimbic cortex of male mice due to EcoHIV infection. Within the nucleus accumbens (NAc), sex-specific inhibitory effects of EcoHIV infection on Iba1+ cell density were observed along with shifts in microglia morphology. Altogether, these results indicate that EcoHIV infection can enhance sucrose-seeking behavior under extinction conditions, that this is not due to enhanced motivation for sucrose, and that such behavioral impairments may be sex-specifically mediated by microglial dysregulation within corticostriatal reward circuitry.

Supported by NIH grants DP2DA051907 (JMB), DP2DA051907-01S1 (JMB), & F32DA060768 (MDN)

#### 43. Acute Neuroinflammation in Response to SARS-CoV-2 Shows Variant-Specific Patterns Shaped by Age and Sex in hACE2 Knock-in Mice

Narayanan, M, MS^1^, Krishna, VD, PhD^1^, Kim, M^2^, Chang, A^3^, Low, WC, PhD^4^, Ling, L, PhD^2^, Cheeran, MCJ, PhD^1^; ^1^Veterinary Population Medicine, College of Veterinary Medicine, University of Minnesota, St Paul, MN, 55108 United States. ^2^Department of Experimental and Clinical Pharmacology, College of Pharmacy, University of Minnesota, Minneapolis, MN, 55414 United States. ^3^Graduate Program in Neuroscience, Medical School, University of Minnesota, Minneapolis, MN, 55414 United States. ^4^Department of Neurosurgery, Medical School, University of Minnesota, Minneapolis, MN, 55414 United States.

SARS-CoV-2 has been associated with diverse acute and long-term neurological manifestations, despite its limited neuroinvasive capacity. Although epidemiological evidence indicates a variant-specific difference in systemic disease severity, the corresponding neurological impact and the underlying mechanisms are largely unexplored. Moreover, clinical outcomes of COVID-19 differ by biological sex and age, underscoring the need to unravel the interplay of these factors on the pathogenesis of neuroCOVID. To address this issue, we infected young (5 months) and aged (10-13 months) human ACE2 knock-in (hACE2-KI) mice with Beta, Delta and Omicron variants of SARS-CoV-2 and assessed viral burden and neuroinflammatory responses five days post infection. Delta variant -infected animals exhibited significantly higher pulmonary viral loads across age groups, with young females showing greater viral burden than age-matched males. Aged mice displayed robust upregulation of pro-inflammatory cytokines and disease-associated microglial markers in the brain. Sex-dependent differences were most pronounced following Delta infection, highlighting a strong interplay between viral variants, age, and sex in driving acute neuroinflammatory responses. Overall, our findings reveal that SARS-CoV-2 variants elicit distinct neuroimmune signatures that are modulated by both age and sex, providing insight into the epidemiological heterogeneity of neuroCOVID and informing future therapeutic strategies. Supported by NIH

#### 44. Perinatal Fentanyl Exposure Reprograms Microglial Development and Neuroimmune Signaling Across Mesocorticolimbic Circuits

Olusakin, J, PhD, Nicot, A, Mclnerney, J, Franco, D, Bertelsen, K, Lobo, MK; Neurobiology, University of Maryland School of Medicine, Baltimore, MD, 21201 United States.

Prenatal opioid exposure continues to increase worldwide and is associated with neonatal opioid withdrawal syndrome and long-term neurodevelopmental risk. Further, evidence suggests that opioids perturb early neuroimmune signaling, but how such disruptions unfold across development remains unclear. Here, using a mouse model of perinatal fentanyl exposure (PFE), we previously identified sex-specific behavioral adaptations in adolescent mice with males display heightened anxiety-like behavior and females exhibiting reduced motivation to groom. To explore neuroimmune mechanisms that may underlie these outcomes, we performed a custom NanoString transcriptomic analysis across six developmental timepoints (from P1 to P35) from the prefrontal cortex, nucleus accumbens, and ventral tegmental area. PFE produced coordinated, sex- and region-specific dysregulation of microglial homeostatic and activation markers, including P2ry12, Cx3cr1, Csf1r, Cd68, Il18, and S1pr1 suggesting altered microglial maturation and immune priming during critical periods of circuit formation. Ongoing RNAscope analyses aim to validate cell-type specificity of molecular markers across mesocorticolimbic circuits, and future studies will employ CRISPR-based targeting of candidate genes to test their causal contribution to PFE-induced behavioral dysfunction.

#### 45. Mitochondrial Antiviral Signaling (MAVS) Disruption in HIV and Substance Exposure: Basis for an Immunomodulatory Peptide Rescue Strategy

Ortiz, M, MD^1^, Jirakanwisal, K, PhD^1^, Fongsaran, C, PhD^1^, Valdebenito, S, PhD^2^, Eugenin, E, PhD^2^, Cisneros, I, PhD^1^; ^1^Department of Pathology, University of Texas Medical Branch, Galveston, TX, 77555 United States. ^2^Department of Neurobiology, University of Texas Medical Branch, Galveston, TX, 77555 United States.

HIV-1 pathogenesis is strongly shaped by immune interactions, particularly in individuals with co-occurring substance use disorders (SUD). Cocaine and fentanyl, two highly prevalent substances among people living with HIV, exacerbate immune dysfunction, increase viral persistence, and accelerate disease progression, yet the molecular mechanisms underlying these effects remain incompletely defined. Emerging evidence suggests that impaired Mitochondrial Antiviral Signaling Protein (MAVS) function contributes to dysregulated antiviral immunity by weakening type I interferon (IFN-I) responses essential for viral control. My doctoral research investigates how cocaine and fentanyl exposure disrupt CD4^+^T cell effector differentiation and the MAVS–IFN-I axis during HIV-1 infection. Using human primary CD4^+^T cells exposed to cocaine, fentanyl, or their combination in an HIV-1 infection model, we assess alterations in effector phenotypes and quantify cytokine and chemokine responses to characterize the resulting immune environment. To directly evaluate the impact on innate antiviral signaling, we analyze the expression of key proteins involved in MAVS-dependent IFN-I pathways. These mechanistic studies form the foundation for the development of a MAVS-mimetic therapeutic peptide, modeled after the naturally occurring MAVS minor genotype known for its enhanced antiviral capacity. The goal of this peptide is to restore or augment innate antiviral signaling in cells compromised by both HIV infection and substance exposure. If successful, this platform may extend beyond HIV–SUD syndemic. Supported by R01DA052263, NS137812

#### 46. Adolescent Exposure to Polychlorinated Biphenyls Disrupts the Gut-Brain Axis

Pierre, ET, BS, Lara, E, Torices, S, PhD, Kerr, N, PhD, Toborek, M, MD, PhD; Department of Biochemistry and Molecular Biology, University of Miami Miller School of Medicine, Miami, FL, 33136 United States. ^2^Department of Neurological Surgery, University of Miami Miller School of Medicine, Miami, FL, 33136 United States.

Depression is a leading cause of disability worldwide, arising from biological, psychological, social, and environmental factors. Adolescents (ages 10-19) are disproportionately exposed to these factors as their neural, immune, and endocrine systems undergo critical maturation supporting cognitive and emotional regulation. Central to this process is the gut-brain axis (GBA), which integrates these systems to influence cognition and emotion. This project investigates how environmental toxicants, particularly polychlorinated biphenyls (PCBs), disrupt GBA signaling and contribute to inflammation-driven behavioral changes. Although banned in 1979, PCBs are persistent organic pollutants that continue to contaminate the food chain and older buildings, including schools, where they were formerly used in building materials as caulks and sealants. The Toborek Lab has shown that PCBs disrupt circadian regulation and blood-brain barrier integrity in brain endothelial cells, mechanisms that may contribute to inflammation-driven mood dysregulation. With an adolescent mouse model, we identified PCB-induced behavioral phenotypes showing deficits in working and recognition memory, reduced sociability, increased depressive-like behavior, and molecular phenotypes showing decreased region-specific gut-intestinal tight junction expression and increased tissue-specific gut-intestinal and brain circadian clock expression. Future work will identify microbial and inflammatory pathways mediating these outcomes to inform GBA-targeted interventions to advance mood disorder treatment and management.

#### 47. Dolutegravir Prevents Nuclear Translocation of HIF-1 Alpha in Cardiac Myocytes

Ramasamy, M, PhD^1^, Namvaran, A, PhD^1^, Fisher, R, BS^1^, Malgija, B, PhD^2^, Edagwa, B, PhD^1^, Bidasee, K, PhD^1^; ^1^Pharmacology and Experimental Neuroscience, University of Nebraska Medical Center, Omaha, NE, 68198-5800 United States. ^2^Centre for Computational Informatics Research and Innovation, MCC-MRF Innovation Park Madras Christian College, Tamil Nadu, Chennai, 600 059 India.

Earlier we reported increased HIF-1α in cardiac homogenates from HIV-infected humanized mice without and with dolutegravir (DTG)/tenofovir disoproxil fumarate (TDF)/emtricitabine (FTC) treatment for twelve weeks. However, in immunofluorescent studies, the majority of the HIF-1α in myocytes from DTG/TDF/FTC-treated animals remained cytoplasmic, suggesting that DTG/TDF/FTC treatment were inhibiting nuclear translocation of HIF-1α, thereby preventing metabolic shifting under hypoxia/ischemia. In this study immunofluorescence and molecular docking were used to delineate the reason and the consequence of cytoplasmic accumulation of HIF-1α. Treating H9C2 cardiac myocytes with DTG (1.5 and 3.75 μM) for 2 hrs at ambient oxygen (20%) resulted in perinuclear accumulation of HIF-1α, and an increase in ROS generation. HIF-1α upregulated by therapeutic levels TDF and FTC were translocated to the nucleus with minimal ROS generation. Docking studies indicated strong interactions of DTG in the prolyl dehydrogenase hydroxylation domain of HIF-1α, the major and minor interacting sites on importin α where the nuclear localization of HIF-1α binds and with regions of importin β that interacts with the FG-Nups of the nuclear pore complex. In Seahorse mitochondrial flux assays, TDF, and FTC but not DTG increased basal oxygen consumption and ATP. All three drugs increased FCCP-induced maximal respiration. These new data suggest that chronic DTG exposure may be contributing to the early-onset heart failure reported in people living with HIV-1 infection. Supported by 1R01HL164306-03

#### 48. HIF-1 siRNA Encapsulated Extracellular Vesicle Therapy Protects Against HIV-Associated Neurological Deficits

Ray, S, PhD, Kumar, M, Chemparathy, DT, PhD, Dash, PK, PhD, Sil, S, PhD; Pharmacology & Experimental Neuroscience, University of Nebraska Medical Center, Omaha, NE, 68198 United States.

HIV-associated neurocognitive disorders (HAND) persist in almost 50% of people with HIV on antiretroviral therapy, often accompanied by Alzheimer’s-like pathology, with Aβ accumulation reported in 29% of middle-aged individuals with HIV on antiretroviral therapy. We previously demonstrated that hypoxia-inducible factor-1α (HIF-1α) is a key driver of HIV-1 Tat-mediated amyloidogenesis in astrocytes, with amyloid-enriched astrocyte-derived extracellular vesicles (ADEVs) leading to Alzheimer’s-like pathology, synaptic damage, and behavioral changes in naïve mice. However, whether selectively inhibiting astrocytic HIF-1α can reverse HIV-driven Alzheimer’s-like pathology and neurodegeneration in vivo remains unexplored. We hypothesized that intranasal delivery of ADEVs packaged HIF-1α-targeting siRNA could mitigate HIV-induced neuropathology. Using HIV-infected CD34+ NSG humanized mice, we administered HIF-1α siRNA-loaded ADEVs intranasally, achieving targeted delivery to the hippocampus and cortex. This intervention reduced expression of HIF-1α, amyloid precursor protein, AβmOC64, amyloid fibrils, phosphorylated tau, dampened glial activation, partially restored loss of synaptic proteins in the brain regions & partially improved impairments in spatial memory and anxiety-like behavior. These findings establish HIF-1α as a central regulator of HIV-associated Alzheimer’s like pathology and validate ADEV-mediated HIF-1α siRNA delivery as a targeted, non-invasive therapeutic strategy for HAND and other Alzheimer’s-like disorders.

This work was supported by grants R21AG069541(NIH) and UNMC startup funds to S Sil.

#### 49. Longitudinal Brain Morphometry in Early Substance Users in Relation to Multilevel Psychosocial Risks from the ABCD Study

Rodriguez Rivera, PJ, MPH, Cloak, C, PhD, Shichman, L, MS, Ernst, T, PhD, Chang, L, MD, MS; Department of Diagnostic Radiology and Nuclear Medicine, University of Maryland, School of Medicine, Baltimore, MD, 21201 United States.

INTRODUCTION: Early substance use (ESU) before age 16 is linked to psychosocial vulnerability and altered neurodevelopment, but the temporal ordering among psychosocial risk, ESU, and brain development remains unclear. We examined trajectories of brain morphometry across adolescence in relation to psychosocial risk and ESU phenotypes.

METHODS: ABCD Study Data Release 6.0 (baseline–Year 7) were analyzed. ESU phenotypes were derived using latent class analysis of past-year self-report and toxicology screens across visits, defining abstainers, earlyonset (≤13 years), and mid-adolescent onset (14–17 years) groups; class membership was treated as time-invariant. Weighted linear mixed-effects models tested ESU class-by-time effects on cortical thickness, surface area, and cortical/subcortical volume across 34 bilateral regions, adjusting for demographic, developmental, familial, and imaging covariates, with random intercepts for participant and scanner IDs. RESULTS: A total of 11,868 participants were followed from baseline (ages 9–10), with 5,056 contributing data by ages 16–17. Compared with abstainers, early- and mid-adolescent onset ESU groups were associated with smaller cortical surface areas and volumes across frontal, temporal, and posterior cingulate regions (all FDR-corrected p<0.05). Effect sizes were moderate (Cohen’s d≈0.30–0.60) and persisted across adolescence.

CONCLUSION: Early substance use may precede with persistent differences in cortical morphometry. Ongoing structural equation models also evaluate temporal ordering between psychosocial risk and ESU trajectories.

Supported by 5U01DA041117-11

#### 50. HIV Infection of Blood-Brain Barrer Pericytes Impairs Mitochondrial Oxidative Phosphorylation in the Neurovascular Unit via Dysregulation of the Electron Transport Chain

Schmidlin, SS, BS^1^, Naranjo, ON, PhD^1^, Torices, ST, PhD^1^, Fattakhov, NF, PhD^1^, Minevich, MM, BS^1^, Gilicinska, MG^1^, Byrareddy, SB, PhD^2^, Stevenson, MS, PhD^3^, Fontanesi, FF, PhD^1^, Toborek, MT, MD, PhD^1^; ^1^Department of Biochemistry and Molecular Biology, University of Miami Miller School of Medicine, Miami, FL, 33136 United States. ^2^Department of Pharmacology & Experimental Neuroscience, University of Nebraska Medical Center, Omaha, NE, 68198 United States. ^3^Department of Medicine, University of Miami Miller School of Medicine, Miami, FL, 33136 United States.

Human blood-brain barrier (BBB) pericytes are key neurovascular unit (NVU) cells essential for angiogenesis, capillary blood flow, basement membrane support, and maintaining endothelial cell integrity. Recent studies show that pericytes can support productive HIV infection in vitro, as well as in vivo in mouse pericytes, and in brain samples from patients with HIV-associated neurocognitive impairments (NCI). Importantly, BBB pericytes can establish latent HIV infections, which we confirmed in a co-culture model of human brain pericytes and microvascular endothelial cells. In this study, we hypothesized that HIV infection of BBB pericytes disrupts endothelial cell functions. To test this, we performed RNA sequencing analysis on endothelial cells co-cultured with active or latent HIV-infected pericytes. KEGG analysis identified oxidative phosphorylation (OXPHOS) and reactive oxygen species production as the top upregulated pathways in both conditions. BN-PAGE analysis revealed increased abundance of OXPHOS complexes I, III, IV, and V, with no change in CII. Endothelial cells also exhibited dysregulation of mitochondrial fission and reduced mitochondrial respiration measured by Seahorse XF analysis. We aim to investigate the extent of mitochondrial dysregulation and assess whether modulating mitochondrial dynamics can protect endothelial cells from oxidative stress and inflammation. These findings will help define the molecular mechanisms underlying BBB damage in people living with HIV and may reveal potential targets for the treatment of HIV-associated NCI. Supported by This work was supported by the National Institutes of Health grants HL126559, MH072567, DA059849, MH128022, and DA050528.

#### 51. Gabapentin’s Paradox: From Clinical Promise to Reproductive Threat

Shukri, OS, MD, Hediyal, TAH, PhD, Stone, EMA, BS, Schaal, VLS, BS, Devanaboyina, MD, BS, Foroughi, AF, MS, Yelamanchili, SVY, PhD, Pendyala, GP, PhD; Department of Anesthesiology, University of Nebraska Medical Center (UNMC), Omaha, NE, 68198 United States.

Gabapentin, a widely prescribed anticonvulsant and analgesic is widely used for neuropathic pain and seizure disorders. However, its potential impact on female reproductive health remains largely unexplored and poorly understood. In this study, we investigated the consequences of chronic gabapentin exposure on ovarian function in adult female Fischer 344 rats administered gabapentin (500 mg/kg/day) or saline for 21 days. Transcriptomic revealed profound dysregulation of genes governing steroidogenesis, folliculogenesis, and oocyte competence. Gabapentin treatment markedly upregulated key steroidogenic enzymes (CYP17A1 and CYP19A1), zona pellucida components (ZP1, ZP3), and FSHR and STAG3 (follicular signaling mediators). Conversely, essential regulators of oocyte maturation (LHX2, GDF9, BMP15, FIGLA, FOXL2, NOBOX), estrogen receptors (ESR1, ESR2), steroid biosynthesis (STAR, HSD3B1), apoptosis control (BAX, BCL2, CASP3, TP53), and meiotic DNA repair (MCM8, MCM9, SYCP3, RAD51, XRCC4) were significantly downregulated, along with gonadotropin receptors (GNRHR, LHCGR). Currently, we are evaluating if these molecular perturbations also corroborate with alterations in ovarian morphology and disrupted hormonal signaling, potentially predisposing to infertility. This study provides the first mechanistic evidence of gabapentin-induced ovarian dysfunction and underscore the urgent need to evaluate its reproductive safety particularly given its widespread use and potential for dependency.

Supported by Supported by NIH R01DA059177, Lieberman Endowment and Department of Anesthesiology Development funds to G.P.

#### 52. Productive HIV-1 Infection in CRISPR huCD4/CCR5/C1qbp KI Mice

Zhang, C, PhD, Du , XQ, MS, Yeapuri, P, PhD, Dash, PK, PhD, Sillman, BJ, PhD^1^, Mosley, RL, PhD, Edagwa, B, PhD, Lloyd, K.C, PhD, Gendelman, HE, MD; Department of Pharmacology and Experimental Neuroscience, University of Nebraska Medical Center, Omaha, NE, 68198 United States. ^2^UC Davis Mouse Biology Program, UC Davis Medical Center, Sacramento, CA, 95817 United States.

Rodents cannot be infected with HIV as their CD4, CCR5, and CXCR4 are unique from the human proteins; rodent post-transcriptional restriction factors inhibit viral RNA export, translation, assembly, and release; and robust murine NK and CD8^+^ T cells clear infected cells. Transgenic mice demonstrate random transgene insertion arrest virion production. Here, we report a novel CRISPR-Cas9 knock-in (KI) mouse that sustains HIV infection through targeted insertion of humanized CD4, CCR5, and C1qbp[D106G] at their respective loci. All site-specific CRISPR KIs were confirmed using RT-qPCR and Sanger Sequencing. Genotypic and flow cytometry tests demonstrated CD4+ T-cells isolated from peripheral blood, spleen, thymocytes, and lymph nodes. Each of the cells expressed huCD4/CCR5 in their correct orientation. Ex vivo HIV-1ADA (MOI = 0.1) challenge in rodent PBLs led to productive HIV-1 replication. Infectivity was affirmed after intraperitoneal HIV-1ADA (105 TCID50) challenge and infection demonstrated by plasma viral load and DNA (qPCR/ddPCR). Stained splenocytes from infected animals and formalin-fixed spleen tissues affirmed HIV-1p24 antigen expression. CD8+ T-cell depletion accelerated viral infection. This CRISPR KI mouse model enables physiologically regulated expression of HIV entry receptors and represents an advance over transgenic systems, providing a critical platform for therapeutic and vaccine research.

### Investigators / General Poster Abstracts (Abstracts #53 - #115)

#### 53. Cocaine-Mediated Microglial Inflammation Involves PANoptosis

Adeagbo, IA, BS, Oladipo, E, Deshetty, UM, Buch, S, Periyasamy, P; Department of Pharmacology and Experimental Neuroscience, University of Nebraska Medical Center, Omaha, NE, 68198 United States.

Cocaine is linked to persistent neuroinflammation and accelerated neuropathology, yet the mechanisms by which cocaine disrupts microglial function remain poorly defined. Z DNA binding protein 1 has emerged as a regulator of PANoptosis, an inflammatory cell death program integrating pyroptosis, apoptosis, and necroptosis. This study is hypothesized to show that cocaine induces PANoptosis in microglia through coordinated activation of these pathways. Herein, human HMC3 microglial cells were exposed to cocaine in dose and time dependent paradigms. PANoptosis related signaling proteins, including Z DNA binding protein 1, inflammasome components, caspases, and necroptotic markers, were analyzed by immunoblotting. Pathway dependency was assessed using selective inhibitors individually or in combination, and sigma-1 receptor involvement was examined using a selective antagonist. Our results demonstrated that cocaine induced dose and time dependent microglial activation, cytotoxicity, and upregulation of Z DNA binding protein 1, with concurrent activation of pyroptotic, apoptotic, and necroptotic signaling. Inhibition of individual pathways provided partial protection, whereas combined inhibition significantly reduced cell death, confirming PANoptotic dependency. Sigma-1 receptor blockade attenuated Z DNA binding protein 1 induction and inflammatory signaling. Overall, these findings identify PANoptosis as a mechanism of cocaine induced microglial injury and suggest upstream therapeutic targets for mitigating cocaine associated neuroinflammation.

#### 54. Characterizing The Effects of Historical Cocaine Use Disorder on HIV Neuropathogenesis and Cognitive Health In People With HIV

Adeyemi, O, MS^1^, Hills, C, MS^1^, Veenhuis, R, PhD^2^, Rubin, L, PhD^3^, Berman, JW, PhD^1^; ^1^Pathology Department, Albert Einstein College of Medicine, Bronx, NY, 10461 United States. ^2^Molecular and Comparative Pathobiology, Johns Hopkins University Medical Center, Baltimore, MD, 21287 United States. ^3^Neurology, Johns Hopkins University Medical Center, Baltimore, MD, 21287 United States.

While antiretroviral therapy has greatly improved the survival and quality of life of people with HIV (PWH), 15-50% of the 40 million PWH develop HIV-associated cognitive impairments, even when taking suppressive antiretroviral therapy. However, the mechanisms that mediate this are not fully characterized. HIV neuropathogenesis is mediated, in part, by transmigration of infected CD14+CD16+ intermediate monocytes across the blood-brain barrier (BBB) to CCL2, a chemokine increased in the CNS of PWH. These monocytes can infect and activate brain parenchymal cells that can function as long-lived viral reservoirs and release inflammatory cytokines and neurotoxic factors that injure neurons, contributing to HIV-associated cognitive impairment. Another comorbidity that impacts cognitive impairments in PWH is Cocaine Use Disorder (CUD), which is persistent cocaine use with difficulty in terminating use and chronic relapse. Many cognitive deficits associated with chronic use persist despite cessation, making it vital to examine Historical Cocaine Use Disorder (CUDH), which is past CUD without current use. One study showed that PWH with CUDH had significant deficits in executive function compared to those with only HIV, only CUDH, or neither condition. We are examining transmigration of peripheral blood subsets, specifically intermediate monocytes, inflammatory plasma mediators, and in vitro BBB permeability in a cohort of PWH with and without CUDH. Understanding persistent mechanisms underlying cognitive impairment in PWH with CUDH may direct treatments for these cognitive deficits.

#### 55. A Colostrum Nutraceutical for Parkinson’s Disease

Akter, S^1^, Kumar, M^1^, Sil, S^1^, Gendelman, HE^1^; ^1^Pharmacology and Experimental Neuroscience, University of Nebraska Medical Center, Omaha, NE, 68198-5880 United States.

Parkinson’s Disease (PD) is the second most common neurodegenerative disorder, affecting more than ten million people globally. The economic burden of the disease is high, with an estimated annual cost of nearly $52 billion. Available therapeutics for PD are primarily symptomatic. Herein, we developed a novel nonimmunogenic, nutraceutical derived from colostrum for PD. Previously we showed that C-EVs were neuroprotective and anti-inflammatory in a PD model. Transcriptomic performed on midbrain tissues showed that C-EVs suppressed microglial activation and reduced the inflammasome. To produce enough C-EVs of high purity in short time periods new techniques were required. Our laboratory introduced the EXODUS (novel EV Isolation) as an advanced, automatic system for purifying EVs from biofluids by employing dual-frequency ultrasonic nanofiltration. This allowed the production of highly concentrated EVs in limited time. The EVs produced using this approach demonstrated potent biological activity, exhibiting neuroprotection and anti-inflammatory responses highlighting their therapeutic potential for PD.

Supported by University of Nebraska Foundation

#### 56. Sex-Dependent Effects of Prophylactic Central TNF Blockade on Pain-Related Behaviors and Macrophage Neurotransmitter Receptor Function during Paclitaxel-Induced Peripheral Neuropathy (PIPN)

Alhadlaq, MW, MS^1^, Ignatowski, TA, PhD^1^; ^1^Department of Pathology and Anatomical Sciences, University at Buffalo/ Jacobs School of Medicine and Biomedical Sciences, Buffalo, NY, 14203 United States.

Background: Chemotherapy can be life-saving, yet many patients develop painful side effects, including peripheral neuropathy (PN), a chronic neuropathic pain (NP) condition, where patients experience burning, tingling, or extreme sensitivity to touch. Paclitaxel, a widely used chemotherapeutic agent, induces PN through inflammatory mechanisms, including increased levels of the pro-inflammatory cytokine tumor necrosis factor-alpha (TNFα). We hypothesized that prophylactic central TNFα blockade would attenuate the development of pain-related behaviors in a sex-dependent manner. Methods: Paclitaxel (4 mg/kg; i.p.) was used to induce NP in male and female Sprague–Dawley rats. A TNFα blocker was administered perispinally 24 hours prior to the first paclitaxel injection. Mechanical and cold sensitivity were assessed using the calibrated von Frey monofilament and acetone drop tests, respectively. Peritoneal macrophages were collected for ex vivo receptor-specific stimulation. Results: Paclitaxel induced both mechanical and cold hypersensitivity in males and females, with no significant sex differences. Yet, TNFα blockade attenuated mechanical hypersensitivity in males and cold hypersensitivity in females. Also, paclitaxel impaired α2-adrenergic receptor (α2-AR) and muscarinic acetylcholine receptor (mAChR) function in macrophages from male rats only, as this impairment was prevented by prophylactic treatment with the TNFα blockade. These findings demonstrate a sex-specific response to TNFα inhibition, suggesting that male and female use distinct inflammatory pathways during PIPN.

Supported by Intramural Funding

#### 57. CCR5 Receptor Targeted Lipid Nanoparticles for HIV-1 Proviral DNA Excision

Ali, MU, BS, Chaudhary, BN, MS, Dey, SS, MS, Gendelman, HE, MD, Panja, SP, PhD; Pharmacology and Experimental Neuroscience, University of Nebraska Medical Center, Omaha, NE, 68105 United States.

Therapeutic interruption of ART in HIV-1 results in viral rebound, the reasons underlying this rebound viremia is the existence of latent viral reservoirs. Latency development in HIV-1 infection is associated with an exclusive increase in CCR5 receptor expression in the latently infected cells; Hence, we formulated CCR5-targeted Lipid Nanoparticles containing an FDA approved ionizable lipid MC3. Encapsulating CRISPR-Cas9 mRNA in LNPs to excise the latent proviral DNA resulted in selective targeting and elimination of HIV-1 from both T-lymphocyte and myeloid cellular compartments. LNPs were formulated by microfluidic mixing, and their mRNA encapsulation characterization was performed by ribogreen assay. Nanoparticle size and zeta potential were analyzed by Dynamic Light Scattering (DLS). Luciferase and cell viability assay was conducted to analyze the nanoparticle’s toxicity profile and differential uptake. CCR5-targeted CRISPR-Cas9 encapsulated LNPs were tested for their HIV-1 excision efficiency in primary macrophages, T-lymphocytic, and monocytic cell lines. The formulated CCR5 receptor targeted LNPs (M5-LNPs) had mRNA encapsulation efficiency of more than 90% and particle size of less than 100nm. The LNPs were non-toxic at dose ranging from 16µg to 1µg per million cells in primary macrophages.M5-LNPs encapsulating CRISPR-Cas9 mRNA achieved higher HIV-1 excision efficiency in both monocytic and lymphocytic cell lines relative to the control LNPs. Conclusively, M5-LNPs also exhibited superior HIV-1 excision efficiency in HIV-1 infected MDMs relative to the control.

Supported by NIH Funding: R01MH121402, R01MH115860

#### 58. Characterizing Mechanisms of Buprenorphine-Mediated Reduction of CCL2-Induced Mature Monocytes Transmigration and the Roles of MOR and KOR in this Process

Atkins, S.A., MS, Gausepohl, A.M., MS, Volsky, D. J., PhD, Kelschenbach, J, PhD, Calderon, T.M., PhD, Berman, J.W., PhD; Pathology, Albert Einstein College of Medicine, Bronx, NY, 10461 United States.

Antiretroviral therapy (ART) significantly improves health outcomes for people with HIV (PWH) but does not eradicate the virus within cells. HIV neuropathogenesis is mediated, in part, by transmigration of HIV+ (cells harboring virus) and HIV- (uninfected) CD14+CD16+ mature monocytes across the blood brain barrier (BBB) to CCL2, which is elevated in the CNS of PWH. This contributes to inflammation and viral seeding of the brain that results in HIV-associated neurocognitive impairment (HIV-NCI) in up to 50% of PWH despite effective ART. There is no treatment for HIV-NCI in PWH on ART. Opioid use disorder (OUD) worsens HIV-NCI. Buprenorphine (BUP) is a semi-synthetic opioid used to treat OUD. Mature monocytes express µ (MOR) and κ (KOR) opioid receptors, to which BUP binds, suggesting BUP may act on these cells. We showed previously that BUP treatment of mice before or after chronic infection with EcoHIV prevents or reverses HIV-NCI. BUP reduces CCL2-induced uninfected mature monocyte adhesion to human brain microvascular endothelial cells, an early step in monocyte transmigration across the BBB. This suggests that BUP may mitigate HIV-NCI. The effects of BUP on CCL2-induced transmigration of HIV+/- mature monocytes, and the roles of MOR and KOR in mediating these effects, remain unclear. Therefore, we are characterizing mechanisms by which BUP reduces CNS entry of HIV+/- mature monocytes. We propose that BUP may serve as a therapy to decrease chronic neuroinflammation in PWH.

#### 59. Monocyte-Derived Macrophages from People with HIV Using Cannabis Induce Distinct Metabolic Phenotypes in Blood-Brain Barrier Cells

Avalos, B, PhD, Ford, M, BS, Funk, K, BS, Crescini, M, BS, Marcondes, MCG, PhD, Iudicello, J, PhD, Fields, JA, PhD; Department of Psychiatry, University of California, San Diego, La Jolla, CA, 92093 United States.

Chronic HIV infection is associated with neuroinflammation and blood-brain barrier (BBB) dysfunction in people with HIV (PWH). Cannabis use may attenuate HIV-associated inflammation; however, its impact on BBB cellular function remains unclear. We hypothesize that conditioned media (CM) from monocyte-derived macrophages (MDMs) isolated from PWH using cannabis promotes homeostasis in endothelial cells and astrocytes. CM was generated from MDMs treated with IL-1β, THC, CBD, or combinatorial conditions. Human endothelial cells and astrocytes were exposed to 10% CM generated from MDMs isolated from PWH with varying cannabis use patterns. Cellular metabolic phenotypes were assessed using Seahorse extracellular flux assays to quantify oxygen consumption rate (OCR) and extracellular acidification rate (ECAR). Expression of BBB-relevant genes, including C3 and GLUT1, was quantified by RT-qPCR. CM from MDMs treated with IL-1β reduced OCR and increased ECAR in human endothelial cells and astrocytes, albeit the effects were modest in endothelial cells. CM from MDMs treated with IL-1β induced expression of C3 and GLUT1 in astrocytes, consistent with observed metabolic changes. However, CM from MDMs treated with IL-1β reduced C3 expression in endothelial cells compared to treatment with IL-1β alone. This study demonstrates that MDMs from PWH, with and without cannabis use, exert differential effects on BBB-associated cells. These results highlight the potential for peripheral immune factors associated with cannabis use to influence BBB cellular responses in PWH.

#### 60. Determining The Impact of Synthetic Fentanyl on Blood-Brain Barrier Responses to Estrogen and Progesterone Fluctuations in The Context of HIV Infection and Neuroinvasion

Basic, M. B., MS^1^, Ramirez, S.H., PhD^1^, Andrews, A.A., MS^1^; ^1^Pathology, Immunology and Laboratory Medicine, University of Florida, Gainesville, FL, 32610 United States.

Despite the effectiveness of antiretroviral therapy, the risk of HIV-infected women experiencing chronic neuroinflammation, dysfunction of the blood-brain barrier (BBB), and HIV-associated neurocognitive disorder continues. These outcomes suggest a critical role for sex-specific biological factors. Fluctuating hormones across the menstrual cycle, such as estrogen and progesterone, have been shown to modulate immune function and the integrity of the BBB. However, the impact of these hormones on HIV replication and entry into the CNS of women infected with HIV remains unknown. In addition, opioid misuse, including fentanyl, may cause elevated immunomodulatory risks as well as an increased risk of HIV infection. Thus, we propose that fentanyl may synergize with sex hormones to increase HIV replication and promote transmigration of infected immune cells across the BBB. Primary human monocytes and macrophages were treated with physiologically relevant concentrations of 17Î^2^-estradiol, fentanyl, or their combination. Combined estrogen and fentanyl exposure increased HIV replication in macrophages, as measured by p24 ELISA. In an in vitro BBB model, estrogen- and fentanyl-treated monocytes exhibited altered migratory behavior across the endothelial barrier. Future studies will incorporate progesterone and a 3D tissue-engineered BBB to model menstrual cycle-associated hormonal fluctuations and define mechanisms driving immune cell neuroinvasion in women infected with HIV. Together, these studies address sex-specific drivers of HIV neuropathogenesis in the context of opioid exposure.

Supported by R01DA058536

#### 61. Cannabis- and Opioid-Associated Alterations in Glucose Metabolism Markers in Postmortem Brains of People With HIV

Boustani, Ali, MD, Hong, DH, MS, Shu, LS, BS, Funk, KF, BS, Fields, JAF, PhD; Department of Psychiatry, UCSD, La Jolla, CA, 92093 United States.

Cannabis and opioid use are common among people with HIV (PWH), with opioids and cannabinoids being among the most frequently used substances. Use of these drugs may modify neuroimmune and metabolic pathways and potentially exacerbate HIV-associated neurocognitive impairment (HIV-NCI). Glucose dysregulation contributes to HIV-NCI, and substance use may modify these metabolic pathways. We hypothesized opioid use impairs brain insulin signaling, whereas cannabinoid use may preserve these metabolic alterations. To test this hypothesis, postmortem brain specimens were procured from the National NeuroHIV Tissue Consortium. Insulin-signaling and glucose metabolism pathways (IRÎ±, IRÎ^2^, IRs, GLUT3, HK1, and PKM2) were analyzed in dorsolateral prefrontal cortex (DLPFC) lysates stratified into the following groups: non-users, opioid users, cannabinoid users, or combined opioid/cannabinoid users. To determine how opioids and cannabis may interactively influence glucose metabolism densitometry of immunoblots was compared between the groups. Opioid use was associated with the reduced protein levels of IRÎ^2^ and HK1, indicating impaired insulin receptor signaling and early glucose metabolism. In contrast, brain tissues from PWH using cannabis and cannabis plus opioids showed preservation of IRÎ±, IRÎ^2^ levels and modest increases in GLUT3. These findings suggest that Insulin-related signaling proteins may be reduced in brain tissues from PWH using opioids. However, cannabis use may moderate or reverse these opioid induced effects.

Supported by NIDA/DA062278; NIMH/MH128108

#### 62. Microglia-Targeted Gene Therapy Using Receptor-Based Raav Delivery

Chandra, R, PhD, Franco, D, BS, Lobo, MK, PhD; Department of Neurobiology, University of Maryland, School of Medicine, Baltimore, Baltimore, MD, 21201 United States.

Microglia play important role in neuroimmune signaling, synaptic remodeling, and circuit plasticity processes strongly implicated in CNS disorders. However, efficient and selective viral based genetic manipulation in microglia in the adult brain remains a major limitation. We are developing a microglia receptor guided AAV delivery by pulling down the AAV library against microglia-enriched membrane proteins. We have selected two microglial membrane markers, TMEM119 and P2RY12, as candidate receptor proteins for analysis. First, we validated antibody specificity in vitro and in vivo. Immunocytochemistry in BV2 and SIM-A9 microglial cell lines demonstrated consistent labeling of TMEM119 and P2RY12. In parallel, immunostaining in CX3CR1-GFP reporter mice confirmed robust microglial TMEM119 and P2RY12 signal, providing proof-of-principle in the nucleus accumbens (NAc), a key region for addiction and mood-related circuitry. Next, we are selecting an AAV capsid background for AAV library generation by evaluating previously studied AAV serotypes. To this end, we packaged a TMEM119 promoter and a set of P2RY12 promoter fragments that drive tdTomato and will be test the efficiency of transduction in mouse NAc. We will also expand to an in vivo barcoded capsid library approach in the NAc to identify optimal capsids for subsequent microglia-directed AAV library development. This AAV screening can yield microglia-selective vectors broadly useful to neuroscience, enabling precise studies of microglia across CNS disease models to accelerate mechanistic and translational research.

#### 63. Polyamine headgroup derived Lipid Nanoparticles Facilitate Enhanced Endosomal Escape and CRISPR-Cas9 mediated HIV-1 Proviral DNA Excision

Chaudhary, BN, MS, Panja, S, PhD, Ali, MU, BS, Dey, SS, MS, Gendelman, HE, MD; Pharmacology and Experimental Neuroscience, University of Nebraska Medical Center, Omaha, NE, 68105 United States.

Despite antiretroviral therapy (ART), the persistence of latent HIV-1 proviral DNA in CD4+ T cells remains a major obstacle to achieving a functional cure. Therefore, CRISPR-Cas9-mediated viral DNA excision is essential. To this end, our laboratory has developed compositionally unique lipid nanoparticles (LNPs) for CRISPR-Cas9/guide RNA (gRNA) delivery to HIV-1 reservoirs for viral elimination. The gRNAs target structurally conserved regions (tat, rev, and gp41) of the HIV-1 genome and excise them. However, the effectiveness of LNP-based CRISPR therapy is limited by insufficient endosomal escape and reduced mRNA translation efficiency. To address this issue, we synthesized a library of polyamine headgroup derived biodegradable ionizable lipids and evaluated their mRNA translation efficacy. Our lead ionizable lipid-based LNPs (Bpip-LNP) achieved significantly higher mRNA translation efficiency than LNPs formulated with the gold-standard ionizable lipids D-Lin-MC3-DMA and ALC-0315. In BALB/c mice, Bpip-LNPs demonstrated a 2-fold higher mRNA translation compared to MC3-LNPs. Furthermore, a single dose of Bpip-LNPs with an optimal CRISPR-Cas9 mRNA/gRNA ratio resulted in approximately 62% elimination of HIV-1 proviral DNA from the latently infected T-lymphocytic cell line. Overall, the polyamine head group in the ionizable lipid demonstrated an improved endosomal escape and enhanced CRISPR-Cas9 delivery to HIV-1 reservoir cells.

Supported by NIH grants: R01MH121402, R01MH115860

#### 64. Lithium Chloride as a Modulator of Mitochondrial Dysfunction in the Context of ART

Consalvo, AM, MS, Garcia Diaz, M, Ross, R, PhD, Al-Harthi, L, PhD, Wallace, J, PhD; Department of Microbial Pathogens and Immunity (MPI), Rush University, Chicago, IL, 60612 United States.

People living with HIV (PLWH) are living longer with effective antiretroviral therapy (ART), yet continue to experience elevated rates of non-AIDS comorbidities strongly linked to mitochondrial dysfunction. Identifying strategies to counteract ART-associated mitochondrial injury is therefore an important priority. Lithium chloride (LiCl) is a modulator of cellular stress responses and mitochondrial homeostasis, but its ability to mitigate ART-induced dysfunction remains incompletely defined. Peripheral blood mononuclear cells (PBMCs) from healthy donors were exposed to ART with or without LiCl for short and longer-term intervals. Mitochondrial function was assessed by measuring cellular respiration, mitochondrial content, oxidative stress, and mitochondrial genome integrity. Exposure to ART alone, including plasma-equivalent concentrations of bictegravir (BIC; 6.15 µg/mL), tenofovir alafenamide (TAF; 0.121 µg/mL), and emtricitabine (FTC; 2.13 µg/mL), which together comprise Biktarvy, as well as efavirenz (EFV), reduced respiratory capacity. In contrast, co-treatment with LiCl improved mitochondrial respiration, indicating partial restoration of mitochondrial function. Molecular analyses suggested changes in mitochondrial genome abundance and stress-response signaling consistent with a protective or compensatory effect. Together, these findings provide preliminary evidence that LiCl counteracts ART-induced mitochondrial dysfunction in immune cells and supports further study of mitochondrial modulators to reduce ART-associated comorbidities in PLWH.

#### 65. Persistence of the SHIV.D Reservoir and Immune Activation in the Central Nervous System during Long-Term ART Treatment

Decker, KA^1^, Mallick, S, PhD^2^, Podgorski, RM, PhD^3^, Gabor, TA^3^, Bar, K, MD^2^, Burdo, T, PhD^1^; ^1^Rutgers Institute for Translational Medicine and Science, Rutgers, the State University of New Jersey, New Brunswick, NJ, 08901 United States. ^2^Department of Medicine, Perelman School of Medicine, University of Pennsylvania, Philadelphia, PA, 19104 United States. ^3^Center for NeuroVirology and Gene Editing, Department of Microbiology, Immunology, and Inflammation, Lewis Katz School of Medicine, Temple University, Philadelphia, PA, 19140 United States.

Though antiretroviral therapy (ART) suppresses viral replication in people with HIV (PWH), viral reservoirs persist. HIV-associated CNS complications are pervasive among PWH on ART, and chronic immune activation due to viral persistence is a likely contributor. Here, we are using a rhesus macaque (RM) model system that utilizes a barcoded transmitted/founder (TF) SHIV.D.191859 (SHIV.D) virus generated by inserting a HIV-1 clade D Env into a SIV backbone. For this study, ten RMs were infected with SHIV.D and treated with ART ten weeks post-infection. Five of the RMs were necropsied after 9 months on ART, and five necropsied after 20 months of ART to simulate long-term ART usage. With periodic and endpoint RM samples, we measured viral loads along with total and intact proviral DNA. Additionally, CNS and peripheral tissues were subjected to RNA/DNAscope to determine distribution of actively replicating and latent SHIV.D and immunohistochemistry to characterize monocyte/macrophage and T cell activity. SHIV.D DNA was detected in myeloid cells and CD4+ T cells in all RMs. SHIV DNA and RNA, shown via RNA/DNAscope, were present in the CNS and peripheral tissues of all RMs, and declined from 9 to 20 months on ART. While immune activation declined with ART, immunohistochemistry showed that CD68+ macrophage and CD3+ T cells remained in the CNS after ART at low levels in all RM. These findings suggest that though ART suppresses viral replication in the SHIV.D model, the peripheral and CNS viral reservoir persists likely promoting chronic immune activation.

Supported by R01 MH128155

#### 66. The Influence of Long-Acting Antiretroviral Drugs on Blood-Brain Barrier Outcomes in The Context of Methamphetamine Exposure

Delorme-Walker, V, PhD^1^, Au, K, BS^1^, Lim, WL^1^, Fields, A, PhD^2^, Furihata, T, PhD^3^, Siqueira Lima, D, MD, PhD^1^, Webb, W, PhD^4^, Billings, E, PhD^4^, Siuzdak, G, PhD^4^, Grant, I, MD, PhD^2^, Cherner, M, PhD^2^, Letendre, S, MD, PhD^2^, Milner, R, MD, PhD^1^, Ellis, RJ, MD, PhD^2^, Grelotti, D, MD, PhD^2^, Iudicello, J, PhD^2^, Marcondes, MCG, PhD^1^; ^1^San Diego Biomedical Research Institute, Maria Cecilia G Marcondes, San Diego, CA, 92121 United States. ^2^University of California San Diego, Maria Cecilia G Marcondes, San Diego, CA, 92121 United States. ^3^Tokyo University of Pharmacy and Life Sciences, School of Pharmacy, Tokyo, Tokyo, 192-0392 Japan. ^4^Scripps Research, Maria Cecilia G Marcondes, San Diego, CA, 92121 United States.

Advances in long-acting injectable (LAI) antiretroviral therapies (ART) present a treatment option for populations with poor ART adherence, such as among people living with HIV (PWH) who use drugs. Methamphetamine (METH) remains a major challenge to control HIV transmission for behavioral and mechanistic reasons. Disruption of the blood-brain barrier (BBB) is a critical underlying factor in neuroinflammation. Using a multicellular BBB in vitro system, we examined the impact of LAI on endothelial health and BBB integrity, especially combined with METH and conditions that mimic the HIV-latent brain. The results suggest that different ART drugs cross the BBB at different rates, and that BBB disruption may facilitate access. The results also indicate that LAI drugs cross the BBB regardless of disruption and are beneficial in the context of HIV and METH. Our findings have implications in pre-exposure prophylactics (PrEP) and for the treatment of PWH who are drug users.

Supported by NIDA R01DA058705 and R01DA059344

#### 67. HIV-1 Tat-Activated Microglial Extracellular Vesicle Triggers Neuronal Ferroptosis

Deshetty, UM, PhD^1^, Chettiar, P, MS^2^, Dravid, SM, PhD^2^, Periyasamy, P, PhD^1^, Sil, S, PhD^1^, Buch, S, PhD^1^; ^1^Pharmacology & Experimental Neuroscience, University of Nebraska Medical Center, Omaha, NE, 68198 United States. ^2^Psychiatry and Behavioral Sciences, Texas A & M University College of Medicine, Texas, TX, 77843 United States.

Background: Extracellular vesicles (EVs) are lipid bilayer-enclosed nanoparticles that mediate intercellular communication through the transfer of proteins, lipids, thereby modulating recipient cell function. In neurodegenerative disorders, including NeuroHIV, EVs contribute to synaptodendritic dysfunction. Objective: This study examined whether ferroptosis-associated cargo within HIV-1 Tat-induced MEVs contributes to neuronal synaptodendritic injury. Methods: BV2 microglia were treated with Tat (100 ng/mL, 48 h), heat-inactivated Tat, or pretreated with deferoxamine (DFO; 2µM, 1 h) prior to HIV-1 Tat exposure. MEVs were isolated by differential ultracentrifugation and applied to primary rat cortical and hippocampal neurons for 48 h. Ferroptosis markers, iron, endolysosomal pH, mitochondrial function, and synaptodendritic integrity were assessed using immunoblotting, immunocytochemistry, Seahorse assays, transmission electron microscopy, electrophysiology, and dendritic spine analysis. Results: Tat MEVs significantly increased ferroptosis-related proteins (TF, TFR1, STEAP3, DMT1, FTH1), iron accumulation, lysosomal deacidification, mitochondrial ROS, and dysregulation excitatory, and inhibitory synaptic markers. These changes were associated with impaired synaptic transmission and reduced mushroom and stubby dendritic spines. MEVs from DFO-treated microglia attenuated these effects and restored neuronal function. Conclusions: HIV-1Tat-induced MEVs mediate ferroptosis-associated neuronal dysfunction, revealing a novel mechanism underlying synaptodendritic injury in NeuroHIV

Supported by Start-up funds of Dr Shilpa Buch

#### 68. Disulfide-Based LNPs with Enhanced Endosomal Escape Facilitates Improved HIV-1 DNA Elimination

Dey, SS, MS, Chaudhary, BN, Ali, MU, Gorantla, S, Panja, S, Gendelman, HE; Department of Pharmacology and Experimental Neuroscience, University of Nebraska Medical Center, OMAHA, NE, 68105 United States.

Lipid nanoparticles (LNPs) are an approved platform for nucleic acid delivery but remain limited by poor endosomal escape (≤5%), restricting their therapeutic potential. Despite major advances, no LNP formulation has been approved for HIV cure. Compared to adenoviral systems that have failed clinically, LNPs with enhanced endosomal escape and cell-specific targeting represent a promising alternative. In this study, we developed disulfide-based ionizable lipid LNPs to improve cytosolic delivery of CRISPR guide RNAs in primary human macrophages. A library of cleavable disulfide-containing ionizable lipids was synthesized and formulated using microfluidics, followed by physicochemical characterization and evaluation of transfection efficiency in human monocyte-derived macrophages using firefly luciferase mRNA. FDA-approved MC3 and ALC-0315 LNPs served as comparators. The lead formulation (BA-LNP), composed of a novel disulfide-based ionizable lipid, DOPC, cholesterol, and DMG-PEG2K, exhibited optimal size, near-neutral charge, ≥94% encapsulation efficiency, and >80% cell viability at therapeutic doses. BA-LNP achieved ∼20-fold higher luciferase expression than MC3- or ALC-0315-based LNPs, consistent with enhanced endosomal escape confirmed by reduced LAMP-1 colocalization. In humanized mice, BA-LNP mediated protein expression in lungs and spleen, whereas MC3 and ALC-0315 preferentially targeted the liver. CCR5-targeted BA-LNP achieved ∼80% proviral DNA excision in HIV-infected macrophages, demonstrating efficient targeting and functional CRISPR delivery.

#### 69. HIV-1 infection of huCD4/CCR5/C1qbp Knock-In Mouse Splenocytes

Du, XQ, MS^1^, Yeapuri, P, PhD^1^, Edagwa, BJ, PhD^1^, Mosley, RL, PhD^1^, Lloyd, KC, PhD^2^, Zhang, C, PhD^1^, Gendelman, HE, MD^1^; ^1^Department of Pharmacology and Experimental Neuroscience, University of Nebraska Medical Center, OMAHA, NE, 68198 United States. ^2^UC Davis Mouse Biology Program, University of California, Davis, Davis, CA, 95616 United States.

Murine models fail to support productive HIV-1 infection. While humanized mouse models were developed their reliance on human cell engraftment has limited their use in any translational study. Transgenic mice likewise are limited due to random integration leading to non-physiological gene expression. To overcome these limitations, we developed human CD4/CCR5/C1qbp knock-in (KI) mice. CRISPR-Cas9 permitted human gene replacements for CD4, CCR5, and C1qbp in immunocompetent C57BL 6N background then confirmed by Sanger sequencing. We now demonstrate following phytohemagglutinin and interleukin 2 activation and CD3 T cell recovery productive HIV-1 replication was sustained for seven days. Progeny virus was measured in cell supernatants by reverse transcriptase (RT) activity, HIV-1 p24 antigens, flow cytometry and ddPCR (for viral DNA and RNA). We demonstrated productive HIV-1 infection in dissociated splenocytes in huCD4/CCR5 C1qbp KI mice. This unique cell-based platform will now allow antiretroviral and HIV-1 cure strategies.

#### 70. Astrocytic HIV-1 Tat Expression to Model Executive Function Impairment in HAND

Duffy, BC, BS^1^, DeSouza, D, MS^2^, Johnson, TP, PhD^2^, Nonnemacher, MR, PhD^1^, Kortagere, S, PhD^1^; ^1^Microbiology & Immunology, Drexel University, Philadelphia, PA, 19129 United States. ^2^National Institute of Neurological Disorders and Stroke, National Institutes of Health, Bethesda, MD, 20892 United States.

Despite advances in anti-retroviral therapy (ART) and its widespread availability, 40 million people live with HIV-1 (PWH) worldwide. While severe manifestations of HIV-1 infection have waned, comorbidities persist including HIV-associated neurocognitive disorders (HAND). Mild forms of HAND persist, with 30 to 50% of PWH exhibiting working memory, attentional, and executive function deficits related to prefrontal cortex (PFC) function. The neurotoxic viral protein Tat has been detected in PWH who are virally suppressed on ART. To understand the effects of Tat produced by astrocytes on PFC mediated tasks and model expression of Tat in the CNS viral reservoir, lentiviral vectors with GFAP promoters were applied in U87MG cultures. Different promoter sequences showed distinct specificity of transgene expression. A lentivirus was designed to encode Tat (101 amino acids) under the GFA104 promoter (LV-GFA104-Tat). In vitro studies show that transduction with the control lentiviral construct at MOI 0.5 causes transgene expression in 10% of U87MG astrocytes. To investigate cognitive effects of low-level astrocytic Tat expression, LV-Tat or LV-control was injected to mPFC of male Sprague-Dawley rats before testing PFC-mediated tasks. Rats receiving LV-Tat exhibited no significant differences on novel object or spatial object recognition, or attentional set-shifting behavior suggesting no impairment of learning and memory or cognitive flexibility. Future analyses will examine the effects of LV-Tat on astrocytic pathways to understand astrocyte specific Tat-mediated glutamate toxicity.

Supported by NIH grants

#### 71. Drugs of Abuse Modulate CNS HIV Infection in a Humanized Mouse Model of NeuroHIV

Dutta, D, PhD, Makarov, E, MS, Thiele, M, BS, Fernandes, A, BS, Gorantla, S, PhD; Pharmacology & Experimental Neuroscience, College of Medicine, University of Nebraska Medical Center, omaha, NE, 68198 United States.

Drug abuse, a major risk factor for HIV infection, is strongly linked with HIV-associated neurocognitive disorders (HAND). Fentanyl and cocaine may enhance HIV susceptibility and promote viral dissemination to the central nervous system (CNS). Using a novel humanized glial mouse model, we investigated how drug abuse alters HIV infection dynamics, tissue distribution, and CNS involvement. Longitudinal in vivo imaging (IVIS) was employed to noninvasively track spatiotemporal HIV spread, including early CNS invasion. Mice were treated with cocaine or fentanyl before and during HIV infection. Plasma and tissue viral RNA/DNA were quantified by ddPCR and RNA/DNAscope, inflammation was analyzed by immune phenotyping, cytokine profiling, and brain transcriptomics. Both drugs significantly increased plasma and tissue HIV RNA compared with controls. RNA/DNAscope revealed increased HIV in brain sections with drug use. IVIS imaging demonstrated enhanced viral replication and accelerated spread to the brain, with earlier/stronger CNS signals with drug treatment. Fentanyl exposure increased CCR5 expression, expanded CD14â°CD16â° intermediate monocytes, and induced marked immune dysregulation, including elevated IL-6, MCP-1, MIP-1Î^2^, MIP-1Î±, and CXCL10. Brain transcriptomics showed heightened immune activation, cellular trafficking, and neuroinflammatory pathways. These findings demonstrate that cocaine and fentanyl exacerbate HIV replication, accelerate CNS viral dissemination, and amplify neuroinflammation, highlighting drug abuse as a critical driver of HIV disease progression and HAND.

Supported by 5R01DA054535-05

#### 72. The Role of NLRP3 Inflammasome in Opioid-Induced Neurochemical Markers, Therapeutic Effects, and Adverse Effects in Male Mice

El-Hage, N, PhD^1^, Rodriguez, M, PhD^1^, Veeragoni, D, PhD^1^, Carbajal, C, MS^1^, Owens, F, PhD^1^, Eans, SO, MS^2^, Williams, AK, BS^2^, Shinouchi, R, PhD^2^, McLaughlin, JP, PhD^2^; ^1^Department of Cellular and Molecular Medicine, Florida International University, Miami, FL, 33199 United States. ^2^Cellular and Systems Pharmacology, University of Florida, Gainesville, FL, 32610 United States.

Repeated opioid use activates glial cells, which release pro-inflammatory molecules through pathways like the NLRP3 inflammasome. This activation contributes to neuroinflammation, tolerance, and hyperalgesia, reducing the long-term effectiveness of opioids. The study investigated NLRP3’s role in neuroinflammation, synaptic plasticity, and behavioral responses to morphine and fentanyl in mice. Treatments produced dose-dependent pain relief, respiratory depression, and psychostimulation, while increasing levels of cytokines IL-1β, IL-6, IL-18, and TNF-α, as well as chemokines MCP-1 and RANTES. These effects were associated with elevated levels of NLRP3, JNK, p38, and NF-kB, and decreased levels of synaptic markers NMDAR, AMPA, and PSD-95. The NLRP3 inhibitor MCC950 lowered inflammatory cytokines and restored PSD-95 levels. MCC950 also improved opioid effects such as pain relief, respiratory depression, hyperlocomotion, and reward, while reducing tolerance and hyperalgesia. These results suggest that NLRP3 influences opioid-induced inflammation and behaviors, which could increase the risk of overdose.

Supported by National Institutes of Health grants R01DA057884 and R01DA057145

#### 73. Dynamic-Contrast Enhanced (DCE) MRI to Assess Blood Brain Barrier Disruption after Focused Ultrasound in Humanized NSG or NOG Mice

Ernst, T, PhD^1^, Heredia, A, PhD^2^, Jiang, L, PhD^1^, Cunningham, E, BS^1^, Shichman, L, PhD^1^, Bernard, S, BS^1^, Walczak, P, PhD^1^, Chang, L, MD^1^; ^1^Department of Diagnostic Radiology and Nuclear Medicine, University of Maryland School of Medicine, Baltimore, MD, 21201 United States. ^2^Institute of Human Virology, University of Maryland School of Medicine, Baltimore, MD, 21201 United States.

INTRODUCTION: The blood-brain-barrier (BBB) prevents the delivery of most drugs, including antiretroviral therapeutics (ART) for HIV infection, into the brain. Since focused ultrasound (FUS) can cause transient BBB disruption (BBBd), we used dynamic contrast enhancement (DCE) MRI to measure the transfer constant K_trans_ of gadolinium contrast agent (Gd) across the capillary endothelium after BBBd in mice. METHODS: Humanized mice were generated with NSG or NOG mice during neonatal days 2-5, when they were sub lethally irradiated and engrafted with human CD34+ stem cells. At 25-64 weeks of age, 39 mice underwent FUS-BBBd, after injection of microbubbles. DCE MRI was performed at 9.4T using a dynamic 2D FLASH sequence after a bolus injection of Gd (temporal resolution ∼5s). An extended Tofts model was used to quantify K_trans_ in brain regions showing Gd-enhancement on T1W scans (N=17) or in the thalamus of animals without Gd-enhancement (N=16). Scans with fewer than 10 enhanced voxels were excluded. RESULTS: K_trans_ was markedly higher (p<0.0001) in mice with Gd enhancement (9.56±7.3/min) compared to those without enhancement (0.032±0.066/min). No significant sex or age effects were found on DCE measures. CONCULSION: These K_trans_ measurements indicate that FUS with microbubble injection caused marked BBBd in sonicated brain regions, which supports the use of FUS to deliver pharmaceutical agents that cannot cross the intact BBB. Ongoing studies will assess BBBd with K_trans_ for the delivery of lipid nanoparticles containing ART to eradicate HIV reservoirs from HIV-infected brains.

Supported by NIDA-DP1DA53719.

#### 74. A Metabolic Approach to Eliminate Myeloid Viral Reservoirs

Eugenin, E, PhD; Neurobiology, UTMB, Galveston, TX, 77555 United States.

HIV reservoirs are present in several tissues including the CNS. However, the nature and mechanisms by which these viral reservoirs are generated and reactivated within the CNS are unknown. During the last 5 years of funding, we developed an imaging system to detect, quantify, and characterize viral reservoirs in vivo, including the CNS. Using more than 232 human brain samples obtained from HIV-infected individuals under effective ART (1-25 years on ART), we identified the identity and characterized the decay of the viral reservoir as well as the ongoing residual expression of viral proteins. We identified that early after infection, the myeloid viral reservoir (microglia/macrophages) is predominant, and it is responsible for long-term ART. A small population of HIV infected astrocytes was detected, but long-term ART did not reduce the size of the glial viral reservoir. Thus, preventing a cure during the patient life lifetime. During the previous funding period, we identified, using multiple techniques, that long-lived viral reservoirs changed their metabolic profile, accumulating lipids, using low levels of glucose, and mostly utilizing glutamine/glutamate to generate ATP to survive—a “smart” use of a highly abundant energy source in the brain. Blocking these metabolic pathways results in significant apoptosis of HIV reservoirs even in the absence of reactivation, reaching a cure in vitro and in vivo. Thus, we propose to identify whether we can reach viral eradication by exploiting a metabolic weakness.

Supported by NIMH MH128082/MH134761

#### 75. White Matter Pathology in HIV-Associated Neurocognitive Disorder: The Role of Gp120-Induced Myelin Damage

Gamboa, A, MS, Kajikawa, M, BS, Agbey, C, PhD, Mocchetti, I, PhD; Laboratory of Preclinical Neurobiology, Department of Neuroscience, Georgetown University School of Medicine, Washington, DC, 20057 United States.

Neuropathology in HIV-associated neurocognitive disorder (HAND) is multifaceted, involving damage to both gray and white matter. Moreover, recent studies have shown a correlation between HIV, white matter damage, and cognitive decline, despite the use of antiretroviral therapy (ART). Yet, little is known about how HIV infection of the brain causes damage to white matter. The observed white matter pathology could be the result of persistent viral reservoirs, ART, the associated comorbidities, or viral proteins. One potent neurotoxic viral protein is the envelope protein gp120, which is present in the brain of virally controlled study patients treated with ART. Thus, we used a gp120 transgenic (gp120tg) mouse model, which exhibits synaptic reduction in the hippocampus, as well as cognitive deficits, to determine the role of gp120 in white matter damage. White matter damage often involves oligodendrocyte (OL) dysfunction, OL precursor cell (OPC) alterations, and myelin basic protein (MBP) loss. Therefore, to investigate white matter pathology, we analyzed MBP immunoreactivity, protein levels, and OPC populations from the hippocampus of aged gp120tg mice. Immunohistochemical analyses revealed altered MBP intensity and OPC populations in gp120tg compared to age-matched wild-type (WT) controls. Furthermore, we show a significant increase in MBP protein levels compared to WT. Moreover, analyses of these sections by electron microscopy uncovered white matter pathology denoted by areas of aberrant myelin structure. Future studies will establish the mechanisms utilized by gp120.

Supported by 1 R01 NS079172-11

#### 76. Clinically Relevant ART Reduces Osteoclast Metabolic Reserve and Bone Resorptive Function

Goldner, J^1^, Garcia Diaz, M^1^, Das, A^2^, Veerathu, S^2^, Ross, R, PhD^1^, Al-Harthi, L, PhD^1^, Wallace, J, PhD^1^; ^1^Microbial Pathogens and Immunity, Rush University, Chicago, IL, 60612 United States. ^2^Biology, Illinois Mathematics and Science Academy, Aurora, IL, 60506 United States.

Approximately 40 million people worldwide are living with human immunodeficiency virus (HIV), according to the World Health Organization. Although antiretroviral therapy (ART) effectively suppresses viral replication, people living with HIV remain at elevated risk for age-related comorbidities compared to the general population. Emerging evidence indicates that ART can contribute to cellular stress and toxicity across multiple immune cell types. Monocytes, which give rise to osteoclasts, play essential roles in immune function and tissue homeostasis. While HIV infection has been shown to alter osteoclast function, the effects of clinically relevant ART on osteoclasts remain poorly defined. Primary monocytes were isolated from healthy donors and differentiated toward osteoclasts in the presence of plasma-equivalent concentrations of Bictegravir (BIC, 6.15 µg/mL), Tenofovir Alafenamide (TAF, 0.121 µg/mL), and Emtricitabine (FTC, 2.13 µg/mL), individually or in combination as the fixed-dose regimen Biktarvy. Maturation, bone resorption, and mitochondrial function were assessed. ART exposure impaired osteoclast differentiation and metabolism. Spare respiratory capacity was significantly reduced by both TAF (p=0.0297) and Biktarvy (p = 0.0225) relative to vehicle. Biktarvy also significantly decreased maximal respiration (p=0.0193). TAF-treated osteoclasts displayed impaired maturation and diminished resorptive activity on bone-mimetic substrates. Our findings suggest that Biktarvy has pronounced morphological and physiological effects on osteoclasts, potentially driven by TAF.

#### 77. Nicotine-Associated Enhancement of Recognition Memory and Neuroprotective Signaling in Rodent and Human Experimental Models

Gupta, E, BS, Bishir, M, MS, Chang, SL, PhD; Institute of NeuroImmune Pharmacology, Seton Hall University, South Orange, NJ, 07079 United States.

Epidemiological studies have reported associations between tobacco use and reduced incidence of neurodegenerative disorders such as Alzheimer’s and Parkinson’s disease, prompting interest in the neurobiological effects of nicotine independent of tobacco smoke. Here, we examined nicotine’s effects on recognition memory and memory-related gene expression using rodent and human models. Fisher 344 rats received twice-daily subcutaneous injections of nicotine (0.25 mg/kg) or saline for 17 days, after which recognition memory was assessed using the Novel Object Recognition Test. Nicotine-treated rats spent significantly more time exploring the novel object compared to saline controls. To investigate molecular changes, RNA sequencing data from the prefrontal cortex, hippocampus, and dorsal striatum of nicotine-exposed rats were analyzed (GSE47474). Differentially expressed genes were evaluated using Ingenuity Pathway Analysis, revealing enrichment of signaling pathways related to synaptic plasticity and memory, alongside reduced activity in pathways associated with neural cell death and neurodegenerative processes. In parallel, microarray data from SH-SY5Y human neuroblastoma cells exposed to nicotine (1mM, 1h or 24h; GSE11208) showed similar pathway-level changes after nicotine exposure. Together, these findings link nicotine exposure with behavioral and transcriptomic changes consistent with enhanced memory-related signaling and reduced neurodegeneration-associated pathways, identifying molecular targets that may inform future studies of cognitive and neuroprotective mechanisms.

Supported by National Institutes of Health: R01DA046258

#### 78. The role of inflammation and blood-brain barrier disruption in depression pathogenesis in people with HIV

Hills, C, MS^1^, Cardozo, F, PhD^2^, Gemelli, M, BS^2^, Vasconcello, H, BS^2^, Otero, A, MD^2^, Sharma, A, MD^3^, Jones, DL, PhD^2^, Gabbay, V, MD^2^, Berman, JW, PhD^1^; ^1^Department of Pathology, Albert Einstein College of Medicine, Bronx, NY, 10461 United States. ^2^Department of Psychiatry and Behavioral Sciences, University of Miami Miller School of Medicine, Miami, FL, 33136 United States. ^3^Department of Medicine, Albert Einstein College of Medicine, New York, NY, 10461 United States.

Depression is the most common neuropsychiatric comorbidity in people with HIV (PWH), with nearly twice the prevalence of the general population, and is associated with increased morbidity and mortality. HIV infection induces chronic inflammation despite viral suppression with ART. HIV-associated inflammation may mediate depression pathogenesis by disrupting the blood-brain barrier (BBB) and altering monocyte function. Using a human BBB model, we found that plasma from PWH increased permeability ∼2-fold compared with plasma from people without HIV (PWoH) (p=0.02). We are conducting a study recruiting PWH and PWoH who undergo assessments for depression severity and psychosocial measures. We evaluate peripheral inflammatory mediators and markers of endothelial/ BBB damage, BBB permeability, and PBMC transmigration for each participant and will use multivariate analyses to assess associations of these measures with depression severity. The following analyses are still ongoing. To date, sE-selectin and sICAM, markers of endothelial injury, are significantly higher in PWH compared to PWoH (p < 0.05). There is a trend of higher sE-selectin and sICAM in PWH with moderate versus mild depression, not seen in PWoH. For PWH, there is a trend of higher sE-selectin in those with a history of depression, while sICAM is higher in those with active depression. Elevated plasma sE-selectin and sICAM may signify BBB damage that contributes to depression in PWH. These molecules may be biomarkers to identify those with active depression and track depression severity/recurrence in PWH.

Supported by R01MH131207

#### 79. Inhibiting GCPII Restores Cognitive Deficit in A Murine Model of HIV

Huang, MH, MD, PhD^1^, Zheng, YZ, MD^1^, Maragakis, RM^1^, Pietri, PP, BS^1^, Su, YS, MD, PhD^1^, Alt, JS, BS^1^, Wu, YW, BS^1^, Liyanage, WL, PhD^1^, Finney, CT^1^, Thomas, MT^1^, Peters, DP, PhD^1^, Zhu, XZ, MD, PhD^1^, Kannan, RK, PhD^1^, Rais, RR, PhD^1^, Slusher, BBS, PhD^1^; ^1^School of Medicine, Johns Hopkins University, Baltimore, MD, 21205 United States.

Despite effective antiretroviral therapy, many people living with HIV (PLH) experience cognitive impairments. Magnetic resonance spectroscopy (MRS) studies in PLH showed that higher brain N-acetyl-aspartyl-glutamate (NAAG) levels correlate with better cognition (Wiseman et al, 2025). NAAG is hydrolyzed by glutamate carboxypeptidase II (GCPII), whose activity elevates with inflammation. We hypothesized that GCPII inhibition would elevate NAAG and improve cognitive outcomes. EcoHIV-infected mice were treated with selective GCPII inhibitor 2-PMPA 3 weeks post-infection. After 2 weeks of daily dosing, mice underwent social interaction, novel object recognition, and fear conditioning tests. CSF NAAG levels were measured by LC-MS/MS and cortical synaptic and dendritic integrity was assessed by immunofluorescence. 2-PMPA increased CSF NAAG ∼8-fold, reversed EcoHIV-induced behavioral deficits and restored synaptic density and dendritic structure. Given the central role of microglia in HIV neuropathogenesis, we next tested a microglia-targeted hydroxyl PAMAM dendrimer conjugate of 2-PMPA (D-2PMPA). Cy5-labeled D-2PMPA localized preferentially to microglia in EcoHIV-infected brains. Three-times-weekly dosing produced therapeutic effects comparable to 2-PMPA at an 8.3-fold lower dose. These findings support GCPII inhibition as a therapeutic strategy for HIV-associated neurological disorders. Microglia-targeted dendrimer delivery further enhanced 2-PMPA potency, positioning D-2-PMPA as a promising next-generation therapeutic for modulating neuroinflammation and glutamatergic dysfunction.

Supported by P30 MH075673

#### 80. Random Forest Analysis Identifies Transcriptomic Mechanisms Underlying Binge Ethanol-Induced Splenic Atrophy

Huang, W^1^, Bishir, M^1^, Abdul Azeeze, MST, PhD^1^, Chidambaram, SB, PhD^2^, Chang, SL, PhD^1^; ^1^Institute of NeuroImmune Pharmacology, Seton Hall University, South Orange, NJ, 07079 United States. ^2^Department of Pharmacology, JSS College of Pharmacy, JSS Academy of Higher Education & Research, Mysuru, Karnataka, 570015 India.

We have reported that binge ethanol (EtOH) induces splenic atrophy in adolescent F344 rats in a sex-dependent manner. Despite receiving identical EtOH treatment regimens, both male and female rats exhibited differential reductions in spleen size. This study aimed to identify the molecular mechanisms driving such differential effects. F344 rats received 3-day binge EtOH (4.8 g/kg/day; 52% w/v; i.g.) at pubertal onset and were sacrificed 24h after the final dose for spleen collection. Total RNA was isolated from individual spleens and subjected to RNA sequencing. Differentially expressed genes (DEGs) were identified relative to water-treated controls. A machine learning-based random forest classifier trained on normalized splenic gene expression data stratified by spleen weight captured transcriptomic changes associated with differential EtOH effects. Feature importance scores identified the top 500 predictive genes from 12,331 total genes in males and females, which were analyzed by Ingenuity Pathway Analysis for functional enrichment. Results revealed inhibition of cell cycle and chromatin regulatory pathways in males and activation of inflammatory pathways in females. This unbiased, high-dimensional random forest analysis identified robust transcriptomic signatures underlying differential EtOH-induced spleen atrophy, highlighting suppressed cell proliferation in males and enhanced inflammatory responses in females.

Supported by NIH AA025964

#### 81. A User-Friendly Quality Control and Visualization Pipeline for 3D-Multi-Electrode Array Recordings from Human Brain Slice Cultures

Iqbal, W, PhD^1^, Nash, B, PhD^1^, Kelschenbach, J, PhD^1^, Ghosh, B, PhD^1^, Van Duyne, R, PhD^1^, Jackson, J, PhD^1^, Sarkar, A, MD, PhD^2^, Meucci, O, MD, PhD^1^; ^1^Department of Pharmacology and Physiology, Drexel University College of Medicine, Philadelphia, PA, 19102 United States. ^2^Department of Neurology, Drexel University College of Medicine, Philadelphia, PA, 19102 United States.

Multi-electrode arrays (MEAs) are popular tools to study broad neuronal activity using in vitro models of disease. Recently developed 3D-MEAs have improved our ability to record human brain slice cultures due to better electrode positioning and higher sensitivity than standard planar MEAs. However, 3D-MEAs can be more prone to noise from intrinsic and extrinsic sources that confound downstream analyses. Analysis tools from makers like Multi Channel Systems (MCS) facilitate recording and basic feature extraction but have limited QC abilities for noise and data visualization, and alternative toolkits often require computational expertise or cannot easily integrate into MCS workflows. Therefore, we developed user-friendly MATLAB scripts that work alongside MCS software to detect noisy signals prior to spike detection and visualize data outputs. Our QC pipeline streams MCS RawData, estimates baseline noise per channel in user-defined segments, flags persistently noisy or inactive electrodes using a robust median/MAD criterion, and detects transient global artifacts via a spatial consensus rule across stable channels. We tested the pipeline using several control recordings and successfully identified noisy channels/sections from 3D-MEA recordings of human brain slices to remove from downstream analyses. The post-analysis script then visualizes MCS-derived metrics in user-defined heatmaps and raster plots for additional inspection. Overall, our approach ensures a rigorous analysis of 3D-MEA recordings and will support our ongoing research on cognitive disorders like neuroHIV.

Supported by NIH NIDA R37 DA015014

#### 82. Characterize Oral-to-Blood Microbial DNA Translocation in Individuals with Cocaine Use Disorder

Jiang, Wei, MD^1^, Johnson, D, PhD^1^, Sundararaj, K, PhD^1^, Fitting, S, PhD^2^, McKinnon, J, MD^1^, Berto, Stefano, PhD^1^; ^1^Pharmacology and Immunology, Medical University of South Carolina, Charleston, SC, 29425 United States. ^2^Department of Psychology & Neuroscience, University of North Carolina at Chapel Hill, Chapel Hill, NC, 27599 United States.

Cocaine disrupts gut barriers in animal models, potentially enabling microbial translocation and inflammation in the periphery and central nervous system, but its direct role in inducing inflammation remains controversial. This study aimed to determine if the oral cavity is a source of circulating microbial DNA translocation in individuals with current cocaine use disorder (COC). A cross-sectional study was conducted, comparing 10 COC (via smoking or vaping) and 24 controls in paired saliva and blood. Microbial 16S rRNA V4 region was sequenced in isolated microbial DNA. Single-cell RNA sequencing was analyzed in human PBMC. Saliva from COC, but not plasma, exhibited reduced alpha diversity and altered beta diversity, characterized by enriched Streptococcus and depleted Fusobacterium, Neisseria, and other taxa versus controls. Controls exhibited low to undetectable microbial translocation in plasma. By contrast, plasma Streptococcus and several S. species displayed COC-specific oral enrichment and evidence of translocation into the bloodstream. In vitro, cocaine selectively enhanced S. parasanguinis growth, consistent with COC-enriched oral pathobionts and blood translocation in vivo. S. parasanguinis, but not cocaine alone, induced IL-1Î^2^ and TNF-Î± production in human primary monocytes. scRNAseq further revealed innate immune activation, impaired T cell function, and heightened susceptibility to infection in COC. This is the first study demonstrating that COC via smoking or snorting exhibited compromised oral-to-blood barrier and selective microbial translocation in vivo.

Supported by National Institutes on Drug Abuse

#### 83. Combinatorial therapy with Low Dose IL-2 & Amyloid Beta Targeting Antibody for Alzheimer’s disease

Kadry, R, Bhattarai, S, Foster, E, Qasim, S, Lu, Y, Yeapuri, P, PhD, Mosley, RL, PhD, Gendelman, HE, PhD; Pharmacology and Experimental Neuroscience, University of Nebraska Medical Center, Omaha, NE, 68198-5800 United States.

Alzheimer’s disease (AD) is characterized by accumulation of neurotoxic misfolded amyloid-β (Aβ), leading to neuronal loss and cognitive decline. Soluble oligomeric Aβ (oAβ) is the most neurotoxic isoform and a primary therapeutic target. Although passive immunization with monoclonal antibodies (mAbs) can reduce amyloid burden, clinical efficacy has been limited by antibody mediated neuroinflammation, including amyloid related imaging abnormalities edema (ARIA-E), driven by proinflammatory microglia and effector T-cell (Teff) responses. We hypothesized that combining an oAβ-selective mAb with a regulatory T-cell (Treg) stimulating agent, low-dose interleukin-2 (IL-2), would promote noninflammatory amyloid clearance and improve therapeutic safety. Computational modeling and molecular dynamics simulations identified a unique epitope selectively exposed on oAβ. Mice were immunized with this epitope, and hybridomas were generated and screened using immunoprecipitation and surface plasmon resonance for selective binding to Aβ monomers, oligomers, and fibrils. The resulting novel mAb demonstrated greater selectivity for neurotoxic oAβ compared with FDA approved Aducanumab and Lecanemab. In APP/PS1 transgenic mice, combination therapy significantly reduced brain-infiltrating inflammatory Teff cells, decreased amyloid burden, and improved cognitive performance in the radial arm water maze compared with mAb or IL-2 alone. These findings support a combinatorial immunotherapy strategy to enhance efficacy while minimizing neuroinflammatory adverse effects in AD.

#### 84. Bridging Dendritic Spine Architecture and Neuronal Activity in Healthy Human Cortex: Implications for Chemokine Signaling

Kelschenbach, J, PhD^1^, Ho, C-T, PhD^1^, Nash, B, PhD^1^, Iqbal, W, PhD^1^, Gosh, B, PhD^1^, Jozefick, T^1^, Van Duyne, R, PhD^1^, Jackson, J, PhD^1^, Sarkar, A, MD, PhD^2^, Meucci, O, MD, PhD^1^; ^1^Department of Pharmacology and Physiology, Drexel University College of Medicine, Philadelphia, PA, 19102 United States. ^2^Department of Neurosurgery, Drexel University College of Medicine, Philadelphia, PA, 19102 United States.

The chemokine CXCL12 acts through its receptor CXCR4 as a key regulator of synaptic structure and network activity in the central nervous system. We previously demonstrated that CXCL12-CXCR4 signaling increases dendritic spine density in cortical neurons and reverses spine loss and cognitive impairment in a rodent model of neuroHIV. CXCL12 promotes multiple facets of synaptic plasticity, including de novo spine formation, increased density of thin spines enriched in postsynaptic markers, and reduced interspine distance, consistent with enhanced spine dynamics and clustering. These CXCR4-dependent structural changes are accompanied by functional network adaptations, as evidenced by increased spike frequency in multielectrode array (MEA) recordings. To assess the relevance of these chemokine-driven neuronal effects in a human system, we utilized organotypic cortical cultures derived from healthy surgical resections. These human brain cultures maintain high cellular viability, preserve neuronal, astrocytic, and myeloid populations, exhibit stable dendritic spine architecture, and display spontaneous neuronal activity detectable by MEA. In line with our rodent findings, preliminary data indicate that CXCL12 treatment of the human organotypic culture increases neuronal spine density and baseline neuronal activity. Ongoing studies aim to define the kinetics and mechanisms by which CXCL12 modulates synaptic organization/network function, advancing chemokine signaling pathways as therapeutic targets for cognitive decline.

Supported by NIH/NIDA R37DA015014

#### 85. Roles of Exocytosis in Myelin Membrane Expansion and Plasticity

Lam, M, PhD^1^, Dehtiarov, O, BS^1^, Duan, L, BS^1^, Geraghty, AC, PhD^2^, Yalcin, B, PhD^2^, Monje, M, MD, PhD^2^, Zuchero, JB, PhD^1^; ^1^Department of Neurosurgery, Stanford University, Stanford, CA, 94305 United States. ^2^Department of Neurology and Neurological Sciences, Stanford University, Stanford, CA, 94305 United States.

Myelin accelerates conduction velocity along axons, and loss of myelin leads to cognitive deficits and physical disability. Cells that make myelin coordinate extreme feats of membrane trafficking to wrap axons and form a compact sheath. Despite its complex structure, myelin in the CNS changes in abundance and structure to adapt to new brain activity, requiring spatiotemporal coordination of membrane trafficking in myelin-forming oligodendrocytes. How does neuronal activity regulate membrane trafficking in oligodendrocytes? We previously discovered that exocytosis through the v-SNAREs VAMP2 and VAMP3 drives myelin membrane expansion during development. VAMP2/3 mediate membrane fusion at myelin sheath edges and at the innermost layer, where myelin interfaces with the axon. To determine if neuronal activity stimulates oligodendrocyte exocytosis, we co-cultured primary oligodendrocytes with active versus silenced neurons. Activity from glutamatergic neurons doubles the rate of VAMP3 exocytosis in oligodendrocytes in a calcium-dependent manner. To test how exocytosis sculpts myelin in vivo, we used optogenetic stimulation to induce activity-dependent myelination in the mouse corpus callosum while inhibiting oligodendrocyte exocytosis. We found that oligodendrocyte VAMP2/3 are required for activity-dependent sheath remodeling. Furthermore, motor learning and memory of a skilled forelimb reach task requires oligodendrocyte exocytosis. Thus, we uncover a cellular mechanism for spatiotemporal control of myelin addition that promotes neuroplasticity.

#### 86. NLRP3 inflammasome-Related Microglial Pyroptosis in EcoHIV-Infected Mice

Li, HL, MD, PhD^1^, Mactutus, CFM, PhD^1^, Shtutman, MS, PhD^2^, Booze, RMB, PhD^1^; ^1^University of South Carolina, Psychology, Columbia, SC, 29208 United States. ^2^University of South Carolina, Pharmacy, Columbia, SC, 29208 United States.

HIV-associated neurocognitive disorders (HAND) have become a major clinical concern, which is generally characterized by persistent viral reservoirs and a lower level of chronic inflammation. NLRP3 inflammasome activation exhibited its unique role in the progression of many chronic inflammatory diseases. However, the links of HIV and NLRP3 inflammasome activation remain incompletely understood. In this study, EcoHIV was retro-orbitally injected into C57BL/6J wild-type mice and analyzed at 14-, 30-, and 60-days post-infection. Whole-brain imaging using an innovative light-sheet microscopy (SLICE) system was applied to visualize the timeline of EcoHIV distribution and the colocalization of NLRP3 inflammasome activation in the brain. Additionally, in vitro, BV2 microglial cell line was infected with EcoHIV and treated with MCC950, an inhibitor of the NLRP3 inflammasome, for three days. Results showed that EcoHIV-infected mice showed a peak in NLRP3 expression at two weeks post-infection compared with controls, followed by a modest decline at four weeks. In vitro, EcoHIV-infected BV2 cells exhibited significantly increased EcoHIV-eGFP fluorescence. Treatment with MCC950 significantly reduced EcoHIV-induced NLRP3 expression, suggesting that inhibition of NLRP3 inflammasome activity suppresses both pyroptosis and microglial activation. Together, elucidating the interplay between microglial pyroptosis and NLRP3 inflammasome activation may provide new insights into the pathogenesis and potential therapeutic strategies for NeuroHIV.

Supported by AG082539, DA059310

#### 87. Opioids Leads to Increased HIV Transcription in Mouse Astrocytes

Litwiler, ST, BS, Campbell, LA, PhD; Department of Neuroscience, Georgetown University Medical Center, Georgetown, DC, 20057 United States.

Among people living with human immunodeficiency virus (PLWH), there is a subset of the population that will use opioids recreationally, for pain management, or treatment of opioid use disorder (OUD). Opioids have been shown to reduce immune cell function and increase HIV replication. However, relatively few studies have assessed the probability that opioids may affect astrocytes, known to contribute to the viral reservoir, or exacerbate HIV infection in the brain, thus contributing to HIV-associated neurological disorders (HAND). To assess whether opioids increase HIV transcription in primary astrocytes, we utilized a modified Ecotropic HIV (EcoHIV), which includes a chimeric envelope protein gpr80 that allows for murine infection. The modified EcoHIV also expresses mCherry and NanoLuciferase (EcoHIV NL4-3 Dual Red) which can be used as a quantitative measure of HIV transcription. We hypothesize that opioids could activate the EcoHIV NL4-3 Dual Red promoter in infected astrocytes, which subsequently may promote damage in the CNS. Our data show that opioids such as morphine, methadone and fentanyl consistently increase HIV transcription as measured by NanoLuciferase, while naltrexone and nalaxone could block these effects. Interestingly, we also observed p24 levels increase in the media, indicating that astrocytes could become productively infected in the presence of opioids. A better understanding of the interplay between PLWH and OUD will lead to the development of novel and more effective drug therapies to delay HAND.

Supported by 1R21DA063110

#### 88. HIV gp120 Impairment of Astrocyte Glutamate Clearance via Reduction of Outward K+ Currents

Liu, J, Ton, H, PhD, Xiong, H, MD, PhD; Dept of Pharmacology & Exp Neuroscience, University of Nebraska Medical Center, Omaha, NE, 68198 United States.

Astrocytes express various ion channels and dysregulation of ion channels in astrocytes alters their functions in controlling glutamate uptake and release, leading to pathogenesis of neurodegenerative disorders including HIV-associated neurocognitive disorders (HAND). To understand how ion channel dysregulation contributes to HAND pathogenesis we studied HIV gp120, a potent neurotoxin, on outward K+ current in rat astrocyte cultures. Incubation of astrocytes with HIV gp120 (400 pM, 20-24h) decreased whole-cell outward K+ currents which were sensitive to voltage-gated K+ channel blockers 4-aminopyridine (4-AP), tetraethylammonium (TEA) and Î±-dendrotoxin. The gp120-mediated membrane depolarization resulted in decreased levels of glutamate transporter 1 (GLT-1) expression which were mimicked by TEA, 4-AP or 25 mM K+. The reduction of GLT-1 expression impaired astrocyte uptake of glutamate, one of the voltage-dependent functions in astrocytes. These results suggested that inhibitory effect of gp120 on outward K+ currents impaired glutamate clearance in astrocytes exposed to gp120 and that up-regulation of outward K+ currents may rescue astrocyte function and lower extracellular glutamate concentration during HIV-1 brain infection.

Supported by NIH NIDA R01DA050540

#### 89. JQKD82 Suppresses HIV-1 Replication in Human Monocyte-Derived Macrophages

Ma, J^1^, Wang, X^1^, Zhu, J^2^, Ho, W^1^; ^1^Department of Pathology, Temple University, Philadelphia, PA, 19104 United States. ^2^Department of Pathology, The Ohio State University, Columbus, OH, 43210 United States.

JQKD82 is an epigenetic modulator that inhibits lysine-specific demethylase 5 (KDM5), a cellular factor involved in HIV-1 latency and T cell survival. Although JQKD82 has been shown to affect HIV-1 activation and cytopathic effects in latent T-cell models, its role in HIV-1 infection of macrophages remains unclear. In this study, we examined whether JQKD82 inhibits HIV-1 replication in primary human monocyte-derived macrophages. Treatment of macrophages with JQKD82 at non-cytotoxic concentrations dose-dependently inhibited HIV-1 replication, as evidenced by decrease in virus-induced syncytium formation, virus gag gene expression and p24 protein production. The inhibitory effect of JQKD82 on HIV-1 infection appeared to be at viral entry level, as the treatment of macrophages with JQKD82 prior to infection was more potent for HIV-1 inhibition than postinfection treatment. Infection of macrophages with a pseudotyped HIV-1 virus (NL4-3-ΔEnv-eGFP-Bal) further confirmed that JQKD82 blocked HIV-1 entry. Mechanistic studies showed JQKD82 downregulated the expression of the HIV-1 entry receptors (CD4 and CCR5) in macrophages at both mRNA and protein levels. In addition, JQKD82-treated cells expressed higher level of CC-chemokine RANTES than control cells. These observations support further investigation of targeting KDM5 inhibition as a potential therapeutic strategy for HIV-1 infection.

Supported by DA051893, DA058536, MH134402

#### 90. Effects of MR-Guided Focused Ultrasound Modulation of Nucleus Accumbens on Oral Fentanyl Motivation In Rats

Martin, DA, PhD^1^, Eldirdiri, A, PhD^3^, Ernst, T, PhD^3^, Cunningham, E, BS^3^, Bernard, S, BS^3^, Calu, DJ, PhD^1^, Chang, L, MD^2^; ^1^Neurobiology Dept, University of Maryland, Baltimore, Baltimore, MD, 21201 United States. ^2^Department of Neurology, University of Maryland School of Medicine, Baltimore, MD, 21201 United States. ^3^Department of Diagnostic Radiology and Nuclear Medicine, University of Maryland School of Medicine, Baltimore, MD, 21201 United States.

Transcranial low-intensity focused ultrasound (LIFU) is being explored clinically and preclinically as a non-invasive method of neuromodulation for psychiatric disorders including addiction. Methods: We tested the effect of MR-guided LIFU of bilateral nucleus accumbens (NAc), using MR-acoustic Radiation force-imaging (ARFI) for targeting and estimation of relative LIFU intensity, in anesthetized male and female rats trained to self-administer oral fentanyl. The rats were evaluated on progressive ratio (PR) responding for oral fentanyl/sucrose (70ug/ml in 5% sucrose) 24 hours post-LIFU. Controls were anesthesia-matched but received no LIFU. Results: Prior to LIFU, oral fentanyl/sucrose reward resulted in lower FR3 responding and higher PR responding than sucrose-alone (5%) reward, indicating a role for fentanyl in the motivating and reinforcing properties of fentanyl-sucrose. MR-ARFI-guided LIFU did not significantly attenuate the performance at 24-hours post-LIFU on PR responding for oral fentanyl/sucrose as a group relative to anesthesia-only controls. In a subset of LIFU parameters, MR-ARFI measured displacement during LIFU in NAc correlated inversely with PR performance relative to baseline. A control experiment using bilateral inactivation of NAc with intracranially infused muscimol/baclofen mixture (50 ng + 50 ng/0.5ul/side) reduced PR responding for fentanyl/sucrose solution, indicating PR responding for oral fentanyl-sucrose is NAc dependent. Conclusion: Further work is required to determine LIFU parameters that conclusively reduce NAc-dependent fentanyl motivation.

Supported by NIDA-DP1DA53719

#### 91. Electronic Cigarettes and Alcohol Induce Alveolar Epithelial and Blood-Brain Barrier Injury via P2X7 Receptor Signaling

Mekala, NK, PhD, Togre, NS, Rom, S, Sriram, U, Persidsky, Y; Lewis Katz School of Medicine, Temple University, Department of Pathology and Laboratory Medicine, Philadelphia, PA, 19140 United States.

Previously, we demonstrated that chronic exposure to EtOH and e-Cig stimulates P2X7r leading to extracellular vesicle (EV) release from pulmonary alveolar epithelial cells (PAEC). We assessed EV content by proteomic analysis, evaluated EV induced effects in brain microvascular endothelial cells (BMVEC) and correlated with changes in brain microvessels isolated from mice chronically exposed to EtOH. EVs isolated from media of PAEC exposed to EtOH, acetalaldehyde, ALD (EtOH metabolite), and e-Cig demonstrated increase of several proteins, notably uPA known to break extracellular matrix (ECM). EtOH, ALD, and e-Cig upregulated expression of uPA receptor and inflammasome complex, increased IL1β secretion, caspase-1 activation in PAEC. All were suppressed by P2X7r inhibition in PAEC. EV signaling was evidenced after EVs application to BMVEC by decrease in trans-endothelial resistance (TER, barrier function), intracellular Ca2+ release, MMP9/TGF-β1 secretion (ECM remodeling). PAEC pretreatment with P2X7r inhibitor diminuted uPA content in EVs, improved TER, diminished Ca2+ release and MMP9/TGF-β1 secretion from BMVEC. In mice chronically exposed to EtOH, BBB damage was confirmed by increase of uPA, MMP2, MMP9 and decrease of TIMP1 gene expressions in brain microvessels. Animals treated with P2X7r inhibitor showed decreased amount of EVs and normalized gene expression of uPA, MMP2/9, and TIMP1 in microvessels. Therefore, uPA transported by PAEC derived EVs can negatively affect the BBB integrity confirming lung-brain cross talk during EtOH/e-Cig exposure and regulatory role of P2X7r.

Supported by NIH/DA040619, AA030841

#### 92. Identification of Gene Networks Driving Opioid Withdrawal-Induced Depression in the HIV-1 Tg26 Mouse Model

Moidunny, S, PhD^1^, Amerzhanov, D, PhD^3^, Bartmanski, BJ, PhD^3^, Tao, J, PhD^1^, Volmar, CH, PhD^2^, Kogadeeva, MZ, MD^3^, Beurel, E, PhD^2^, Roy, S, PhD^1^; ^1^Department of Surgery, University of Miami School of Medicine, Miami, FL, 33136 United States. ^2^Department of Psychiatry and Behavioral Sciences, University of Miami School of Medicine, Miami, FL, 33136 United States. ^3^Genome Biology Unit, European Molecular Biology Laboratory, Heidelberg, Heidelberg, 69117 Germany.

Major Depression is prevalent in HIV-patients using opioids. Depression in HIV-patients is associated with higher mortality rates and poor adherence to antiretroviral therapy. Recent studies indicate that opioid use exacerbates HIV-induced neuronal damage and neurocognitive impairments; however, the neural mechanisms underlying depression associated with HIV-1 and opioid use are largely unknown. To address this gap in knowledge, we systematically investigated the effects of opioid treatment on development of anxiety-like and depression-like behaviors in wild-type and HIV-1 transgenic mice (Tg26). Mice were chronically treated with morphine, spontaneously withdrawn and subjected to a battery of behavioral tests: open-field test, sociability test and tail suspension test. At the end of behavioral testing, mice were humanely euthanized and brain tissues collected for RNA seq analysis. We observed that spontaneous and protracted morphine withdrawal induces anxiety-like and depression-like behaviors more severely in Tg26 mice, compared to wild-type controls. Consistently, we observed significant transcriptional changes in pathways and neural circuits associated with mood disorders, including neuronal synaptic transmission (glutamatergic and GABAergic), serotonergic and dopaminergic systems, mitochondrial dysfunction and glial activation. In summary, this study deepens our understanding of the neural mechanisms involved and provides valuable insights that could guide the development of therapies for anxiety and depression in HIV-patients in the future.

Supported by Miami Center for AIDS Research and National Institute on Drug Abuse

#### 93. Chrysin Attenuates HIV Associated Sudden Cardiac Death Risks by Blunting Cardiac Abnormalities

Namvaran, A, PhD^1^, Garcia, J, MS^1^, Ramasamy, M, PhD^1^, Hackfort, B, PhD^2^, Dash, P, PhD^1^, Fisher, R, BS^1^, Edagwa, B, PhD^1^, Gorantla, S, PhD^1^, Bidasee, K, PhD; ^1^Department of Pharmacology and Experimental Neuroscience, University of Nebraska Medical Center, Omaha, NE, 68198-5800 United States. ^2^Department of Cellular and Integrative Physiology, University of Nebraska Medical Center, Omaha, NE, 68198-5800 United States.

Rates of sudden cardiac death (SCD) in people with HIV-1 infection (PWH) are > 4 times higher than those in uninfected individuals, and the reasons for this remain poorly understood. Earlier, we showed that HIV-1-infected humanized mice (Hu-mice) treated with DTG/TDF/FTC recapitulated diastolic dysfunction and abnormalities in electrocardiogram (ECG) that serve as harbingers for SCD. Hypoxia-induced factor-1 alpha (HIF-1α) was also increased in chronically treated animals with DTG/TDF/FTC. While an acute increase in HIF-1α is beneficial in hypoxic regions, persistent elevation can be deleterious. Here, we investigated whether adding the dietary flavone and inhibitor of HIF-1α, chrysin, to the feed would blunt cardiac abnormalities. After 12 weeks of infection, HIV-1-infected Hu-mice developed diastolic/systolic dysfunctions as evidenced by increases in E/A ratio, isovolumic relaxation time (IVRT), and a reduction in fractional shortening. ECG abnormalities, including QT interval prolongation, were also prominent. Adding chrysin to the diet of HIV-infected Hu-mice (40mg/kg/day) lowered their plasma HIV-1 viremia by 25%. Treating HIV-infected Hu-mice with DTG/TDF/FTC for eight weeks starting four weeks after infection blunted systolic dysfunction but not diastolic dysfunction, and ECG changes. Adding chrysin with DTG/TDF/FTC blunted diastolic dysfunction and ECG changes, including QT prolongation. These data also show for the first time that inhibiting HIF-1α is cardioprotective in HIV-1-infected Hu-mice by blunting HIV-associated structural and electrical cardiac remodeling.

Supported by Funded by 1R01HL164306-03

#### 94. HIV Tat-Mediated Astrocytic PANoptosis Induces Neuroinflammation

Oladipo, E, Adeagbo, IA, Agbolade, A, Buch, S, Periyasamy, P; Department of Pharmacology and Experimental Neuroscience, University of Nebraska Medical Center, Omaha, NE, 68198 United States.

Despite effective combined antiretroviral therapy, people living with HIV-1 continue to experience chronic neuroinflammation and cognitive impairment. The HIV Tat protein persists in the central nervous system and induces neurotoxicity and glial stress independent of viral replication. Although apoptosis, necroptosis, and pyroptosis have been implicated in NeuroHIV, growing evidence suggests convergence through PANoptosis, a coordinated inflammatory cell-death program. We hypothesized that HIV Tat induces Z-DNA-binding protein 1 (ZBP1)-dependent PANoptosis in astrocytes. To test this hypothesis, mouse primary astrocytes were exposed to HIV Tat in dose- and time-dependent studies to evaluate astrocyte activation and ZBP1-mediated PANoptosis. Cell death was assessed using lactate dehydrogenase release and propidium iodide staining. Expression of glial fibrillary acidic protein (GFAP), ZBP1, and PANoptosis-associated proteins was analyzed by Western blotting. The involvement of ZBP1 was examined using gene silencing with ZBP1-specific siRNA. Our studies showed that HIV Tat induced astrocytic cell death, evidenced by increased lactate dehydrogenase release and propidium iodide positivity. HIV Tat exposure also increased GFAP, ZBP1, and proinflammatory cytokine expression. ZBP1 knockdown reduced PANoptotic signaling and astrocytic cell death. These findings demonstrate that HIV Tat induces ZBP1-dependent PANoptosis in astrocytes and identify ZBP1 as a therapeutic target in NeuroHIV pathogenesis.

#### 95. Biktarvy, A Potential Algogen for HIV-Associated Pain

Pal, A^1^, Zhou, Yixuan^1^, A, David, PhD^2^, Liang, Y, PhD^2^, Gong, B, MD, PhD^4^, Salisbury, E, PhD^3^, Valdebenito-Silva, S, PhD^1^, G Hemil, G, MD^5^, Eugenin, E, PhD^1^, Yuan, S, MD, PhD^1^; ^1^Department of Neurobiology, University of Texas Medical Branch, Galveston, TX, 77555 United States. ^2^Institute for Human Infections and Immunity (IHII), University of Texas Medical Branch, Galveston, TX, 77555 United States. ^3^Department of Orthopedic Surgery, University of Texas Medical Branch, Galveston, TX, 77555 United States. ^4^Department of Pathology, University of Texas Medical Branch, Galveston, TX, 77555 United States. ^5^Department of Internal Med-Infectious Disease, University of Texas Medical Branch, Galveston, TX, 77555 United States.

Lifelong use of Antiretroviral Therapy helps HIV patients achieve normal lifespan but causes neurological comorbidities such as chronic pain (HIV-PAIN) and sensory neuropathy (HIV-SN). Biktarvy (Bik) is a first line ART regimen in US and Europe, combines two nucleoside reverse transcriptase inhibitors (emtricitabine, tenofovir alafenamide) with one integrase strand transfer inhibitor (bictegravir) and is a rapid viral suppressant. HIV-PAIN affects 30-62% of 39 million HIV patients, contradictory to its safer ART notion. To determine if Bik is algogenic and contributes to HIV-PAIN, we orally administered Bik to male and female mice for 21 days, as 51% of HIV patients are female with more prevalent HIV-PAIN. We used human equivalent dose (to mimic patient daily use) and 3x dose (to fully excavate neurotoxicity and compensating the limited administration time). Both doses induced mechanical allodynia at day 11, persisting to day 21, with females showing earlier and more prominent allodynia. Treated mice also developed heat and cold hyperalgesia without change in nerve conduction velocity of tail sensory and motor nerves. Histologically, hindpaw skin showed degenerated protein gene product 9.5 positive nociceptors (acute pain mediators) and sprouting of growth associated protein 43 positive nociceptors (chronic pain mediators), indicating selective pathological changes and sensory neuropathy. Thus, we conclude Bik as potential algogen, contributing to HIV-PAIN and HIV-SN. Further study will focus on exploring the nociceptive mechanism and developing more effective analgesics.

Supported by NINDS R01NS122571, Pilot grant from UTMB (P30 AG024832), UTMB IHII short-term DPRIT program

#### 96. Neural Progenitor Cell-Derived IGF-1 Modulates Pericyte Responses Following Methamphetamine and HIV Exposure

Park, M, PhD, Levitis, D, BS, Si, J, BS, Toborek, M, MD, PhD; Department of Biochemistry and Molecular Biology, University of Miami Miller School of Medicine, Miami, FL, 33136 United States.

Methamphetamine (METH) use and HIV-1 infection are major contributors to neurovascular injury and blood-brain barrier (BBB) dysfunction, yet the role of neural progenitor cells (NPCs) in regulating vascular responses remains poorly defined. We examined whether NPC-derived insulin-like growth factor-1 (IGF-1) modulates NPC-pericyte signaling during METH and HIV exposure. In a mouse model, METH and HIV co-exposure increased IGF-1 mRNA expression in subventricular zone (SVZ)-derived NPCs and the caudate putamen, while HIV alone elevated IGF-1 in the SVZ. Despite regional transcriptional increases, IGF-1 protein levels were unchanged in the frontal cortex and reduced in plasma, indicating disrupted IGF-1 homeostasis. In vitro, METH and HIV co-treatment suppressed FAK, ERK, and AKT phosphorylation and altered cytoskeletal organization in human NPCs. Conditioned media from METH- and HIV-exposed NPCs reduced ERK, AKT, and FAK activation in primary human brain pericytes. Neutralization of IGF-1 selectively restored ERK and AKT, but not FAK, signaling in pericytes, suggesting pathway-specific regulation. Functionally, NPC exposure to METH and HIV reduced NPC-pericyte adhesion. These findings identify NPC-derived IGF-1 as a paracrine modulator of pericyte signaling and cell-cell interactions, implicating altered neurogenic-vascular communication in methamphetamine- and HIV-associated neurovascular remodeling.

Supported by DA060085, DA060085-02S1, DA050528, DA044579, DA059849, HL126559, MH128022, and MH072567.

#### 97. Social Strain and the Opioid Brain: Mechanisms Underlying Prescription Opioid Use Post Social Defeat in HIV+ Adolescents

Pendyala, G, PhD; Department of Anesthesiology, University of Nebraska Medical Center, Omaha, NE, 68198 United States.

Adolescence is a critical developmental phase marked by physical, cognitive, emotional, and social changes, making it highly vulnerable to adverse experiences with lasting consequences. According to UNAIDS 2023, 1.4 million adolescents live with HIV, underscoring the need to understand psychosocial stress impacts. Social defeat (SD), modeled via a resident-intruder paradigm, simulates bullying-related stress. HIV-1 transgenic (Tg) and wild-type (WT) rats underwent 10 days of SD, followed by behavioral, biochemical, molecular, omics, and drug-seeking analyses in early adulthood. Tg rats exhibited heightened anxiety-like behavior and neuroinflammation, with elevated TNF-Î±, GFAP, Iba-1, and IL-6. Interactome analysis revealed impairments in synaptogenesis, mitochondrial function, and endocytosis. Proteomics analysis showed pervasive downregulation of synaptic vesicle cycle components and exocytosis machinery, with GO terms such as synaptic vesicle cycle and vesicle-mediated transport significantly decreased in Tg SD vs. Tg controls; the SNARE complex was the only downregulated cellular component. Tg rats exposed to SD demonstrated increased oxycodone intake, indicating greater vulnerability to opioid use. These findings suggest repeated SD amplifies HIV-1-induced neuroinflammation, disrupts synaptic and exocytotic functions, and alters mitochondrial/peroxisomal metabolism. Proteomic shifts may reflect glial metabolic reprogramming toward energy-intensive inflammatory states, paralleling enhanced drug-seeking behavior.

Supported by NIH R21DA058588, Lieberman Endowment, Departmental Start-up funds & 2024 Opioid Prevention and Treatment pilot grant to G.P.

#### 98. Morphine Promotes OPRM1 Alternative Splicing to Generate a M-Opioid Receptor Isoform Enhancing Dependence-Related Signaling

Sariyer, IK, PhD^1^, Swingler, M, BS^1^, Donadoni, M, PhD^1^, Cakir, S, BS^1^, Kumar, V, BS^2^, Bishir, M, PhD^3^, Huang, W, PhD^3^, Chang, SL, PhD^3^; ^1^Department of Microbiology, Immunology, and Inflammation, Center for Neurovirology and Gene Editing, Temple University Lewis Katz School of Medicine, Philadelphia, PA, 19140 United States. ^2^Lewis Katz School of Medicine, Temple University, Philadelphia, PA, 19140 United States. ^3^Institute of NeuroImmune Pharmacology, Seton Hall University, South Orange, NJ, 07079 United States.

Clinically used opioids, such as morphine, primarily act through the Î1/4-opioid receptor (MOR), encoded by the OPRM1 gene, which undergoes extensive alternative splicing. More than 20 OPRM1 isoforms have been identified, yet functional characterization has largely focused on the canonical MOR-1 variant. With the emergence of three-dimensional human cerebral organoids (hCOs) derived from induced pluripotent stem cells (iPSCs), it is now possible to model human-specific neuronal responses to opioids more accurately. In this study, we established hCOs as a functional platform to investigate the impact of morphine on OPRM1 pre-mRNA splicing and opioid signaling. Morphine exposure selectively induced the MOR-1X isoform in hCOs and iPSC-derived neurons in a dose-dependent manner. Upon morphine withdrawal, HEK293 cells expressing MOR-1X exhibited markedly enhanced cAMP superactivation, a molecular hallmark of opioid dependence, compared with MOR-1. Furthermore, isoform-specific knockdown of MOR-1X by shRNA effectively abolished this cAMP overshoot. Transcriptomic and gene ontology analyses revealed distinct gene expression profiles and signaling pathways between MOR-1 and MOR-1X activation. Collectively, these findings identify MOR-1X as a morphine-inducible isoform with a potential key role in the molecular mechanisms underlying opioid signaling, adaptation, and dependence in the brain.

Supported by NIH/NIDA, R01DA052284

#### 99. Differential Effects of Itaconate and Citraconate on Microglial Morphology and Functional State: A Quantitative Analysis of Activation and Phagocytic Markers

Sikirzhytskaya, AS, PhD^1^, Aksenova, M, PhD^1^, Li, H, PhD^2^, Sikirzhytski, V, PhD^1^, Ji, H, MS^1^, Altomare, D, PhD^1^, Booze, RM, PhD^2^, Shtutman, M, PhD^1^; ^1^College of Pharmacy, University of South Carolina, Columbia, Columbia, SC, 29208 United States. ^2^Department of Psychology, Cognitive and Neural Science Program, University of South Carolina, Columbia, Columbia, SC, 29208 United States.

The cell-permeable ester form of the mitochondrial metabolite itaconate, 4-octyl-itaconate (4OI), is a potent suppressor of HIV-induced neuroinflammation. Here, we compare the potential of 4OI with the modified itaconate isoform dimethyl-citraconate (DMC). Citraconate inhibits the itaconate-producing enzyme aconitate decarboxylase (Acod1). However, the neuroprotective activity of DMC in the combined treatment of HIV-Tat and cocaine was similar to or exceeded that of 4OI. Furthermore, RNA-seq data show that both compounds activate similar antioxidant and anti-inflammatory pathways. Phenotypic analysis of microglial morphology shows slightly distinct activity. Quantification of Iba1 (reactivity) and CD68 (phagocytosis) markers revealed significant treatment effects on cell area, Iba1 intensity, and CD68 intensity. Correlation profiling showed that HIV-Tat and cocaine disrupted normal morphology-activation coupling, driving cells toward a phagocytic phenotype. DMC partially restored baseline coordination and dampened marker expression, whereas 4OI maintained elevated Iba1 and CD68 levels. Live/dead assays confirmed that these shifts lowered cytotoxicity, indicating genuine metabolic modulation rather than reduced viability. Overall, the results expand the panel of available itaconate derivatives for the treatment of neuroinflammation, with DMC more effectively attenuating microglial activation toward a homeostatic phenotype. Given its higher potency and ease of administration, dimethyl-citraconate is a promising therapeutic candidate.

Supported by AG082539, DA059310, MH106392, NS100624, NIH NIDA R01DA054992

#### 100. Microglia mediate neuronal injury by HIV and Methamphetamine in association with release of pro-inflammatory cysteinyl leukotrienes

Singh, H, PhD, Maung, R, BS, Kaul, M, PhD; School of Medicine, University of California, Riverside, Riverside, CA, 92505 United States.

Microglia are the resident immune cells of the central nervous system and play a crucial role in maintaining brain homeostasis. During HIV infection, activated glial cells release inflammatory and neurotoxic factors that contribute to neurocognitive impairment (NCI), which persist despite combined antiretroviral therapies. Methamphetamine (METH) use among people living with HIV further exacerbates NCI, although the underlying mechanisms remain poorly understood. In this study, we investigated how METH influences HIV infection of microglia and associated neurotoxicity. Induced pluripotent stem cell derived microglia infected with HIV-1BaL (MOI 1) exhibited peak p24 production at day 6 post-infection, followed by a decline through day 12. Exposure to 100ÂµM METH reduced p24 levels at day 6, suggesting partial interference with viral replication. However, conditioned media (CM) from HIV-infected microglia significantly reduced neuronal survival compared with non-infected controls. METH alone and in combination with HIV further exacerbated neuronal loss, indicating that viral load alone does not fully reflect viral pathogenicity. Analysis of CM from microglia infected with HIV-1BaL at MOIs ranging from 0.01 to 1.0 and treated with METH (1-100Î1/4M), revealed increased release of pro-inflammatory cysteinyl leukotrienes, implicating in HIV neurotoxicity. Ongoing studies using male and female derived microglia will further elucidate sex-specific effects. These findings highlight a complex interplay between HIV infection, METH exposure, and microglial-mediated neurotoxic activation.

Supported by R01 MH087332, R01DA052209, R01NS148274, R01DA063181

#### 101. Neonatal Morphine and HIV Synergy Induce Persistent Neuroimmune and Anxiety-Related Transcriptional States

TAO, J, PhD, Antoine, D, PhD, Roy, S, PhD; Department of Surgery, University of Miami Miller School of Medicine, Miami, FL, 33136 United States.

Background: Early-life opioid exposure can disrupt neurodevelopment and heighten vulnerability to anxiety and affective disorders, particularly in individuals with HIV. Methods: Using single-cell RNA sequencing (scRNA-seq), we profiled adolescent brains from wild-type and HIV-1 transgenic (Tg26) female mice exposed to morphine during postnatal days 6–7. Results: Morphine exposure in Tg26 mice resulted in a highly dysregulated microglial phenotype, characterized by the ectopic upregulation of genes encoding neuronpeptides (Avp, Hcrt, and Pmch), while simultaneously showing a reduction in both inflammatory and homeostatic markers (Map3k6, Lgals3, Ccl3). Microglia also showed enhanced expression of dynorphin (Pdyn) and κ-opioid receptor (Oprk1) signaling modules implicated in dysphoria and stress-induced negative effects. Furthermore, transcriptomic mapping revealed cell-type-specific neuronal adaptations: cholinergic neurons upregulated genes linked to anxiety and arousal (Avp, Oxt), GABAergic neurons upregulated genes linked to condition and aversive behavior, whereas glutamatergic neurons enriched for transcripts associated with thigmotaxis and fear behaviors. Conclusions: Together, these findings demonstrate that brief neonatal morphine exposure in an HIV-inflamed milieu induces persistent, cell-type-specific neuroimmune and neurotransmitter reprogramming that engages the dynorphin–KOR pathway and predisposes to anxiety- and aversion-related behaviors.

Supported by National Institute on Drug Abuse Grants

#### 102. Neuroinflammatory Mechanisms Linking Systemic Infection to Exacerbated Ischemic Brain Injury

Torices, S, PhD, Fattakhov, N, PhD, Del Sol, S, Lara, E, Toborek, M, PhD; Miller School of Medicine, University of Miami, Miami, FL, 33136 United States.

People living with HIV experience a higher risk of ischemic stroke and often exhibit poorer neurological outcomes, yet the biological pathways responsible for this enhanced vulnerability are not fully understood. Chronic immune activation and dysregulated neuroinflammatory signaling represent central features of HIV neuropathogenesis and may interact synergistically with ischemic injury to worsen brain damage. To investigate this interaction, we utilized an in vitro model consisting of human astrocytes and brain endothelial cells subjected to oxygen-glucose deprivation followed by reoxygenation (OGD/R) to mimic ischemic stress. Our findings show that astrocytes mount a robust inflammatory response under combined HIV-1 exposure and this activation is significantly reduced by treatment with cenicriviroc (CVC), a dual CCR2/CCR5 antagonist. These results suggest that chemokine receptor-dependent pathways contribute to HIV-driven amplification of neuroinflammation. In parallel, EcoHIV-infected C57BL/6 mice exposed to middle cerebral artery occlusion (MCAO) exhibited heightened glial reactivity, increased inflammatory signaling in the brain, and greater ischemic damage compared to uninfected controls. HIV infection also appeared to exacerbate susceptibility to stroke and worsen post-stroke outcomes. Overall, our data indicates that HIV-enhanced neuroinflammation plays a key role in aggravating cerebral injury after ischemic stroke. Targeting inflammatory signaling pathways may represent a promising therapeutic avenue to improve neurological recovery in individuals living with HIV

Supported by R21NS141704

#### 103. Pericyte Mechanisms are Altered Following Exposure to METH and Secretome of METH-Stimulated Brain Endothelial Cells

Trivedi, JJ, MS, Mekala, N, PhD, Togre, N, PhD, Rom, S, PhD, Sriram, U, PhD, Persidsky, Y, MD, PhD; Department of Pathology and Laboratory Medicine, Lewis Katz school of Medicine, Temple University, Philadelphia, PA, 19140 United States.

Methamphetamine (METH) is a potent psychostimulant, widely abused in the United States and worldwide. METH abuse causes excitotoxicity, glial cell activation, and blood-brain barrier (BBB) damage leading to neurodegeneration. Within BBB, pericytes are tightly connected to endothelial cells and form crucial regulating component maintaining microvessel integrity. Pericyte dysfunction is considered one of the early hallmarks of BBB damage, and reduced pericyte coverage is linked to aggravated neuroinflammation. Yet, mechanisms triggering pericyte dysfunction following METH exposure is not well-known. Using human brain pericytes, we studied pericyte responses to METH by evaluating markers such as PDGFRb, aSMA, ICAM-1, VCAM-1; and found no changes in their expression. However, pericyte mitochondrial respiration was altered, suggesting direct influence of METH on mitochondrial function. Further to understand physiological impact of BBB microenvironment on pericytes, we exposed pericytes to secretome of METH-stimulated brain endothelial cells. We observed that there is decrease in membrane PDGFRb expression and increase in P62 expression, suggesting pericyte activation on METH-mediated endothelial response. Our findings show that pericyte function change with endothelial response following METH exposure. Hereby, we establish that though pericytes might be sensitized following METH exposure, their functions might be dysregulated by additional hit like pro-inflammatory microenvironment.

Supported by NIDA/5R01DA052970 & NIAAA/5R01AA030841

#### 104. HIV Uses Tunneling Nanotubes to Spread Aggregated Proteins and Contribute to HAND

Valdebenito-Silva, S, PhD; Neurobiology, University of Texas Medical Branch, Galveston, TX, 77555 United States.

HIV induces the formation of tunneling nanotubes (TNTs), a dynamic F-actin intercellular structures, which are generated from the viral reservoir to reach surrounding uninfected cells upon reactivation. Furthermore, TNTs enable the direct cytoplasmic exchange of host components, including mitochondria, vesicles, signaling molecules, aggregated neurotoxic proteins, and viral material (including viral RNA and proteins). Here, we identified TNTs as a critical mechanism for the spread of infection and also facilitates the spread of neurodegeneration even in the presence of ART. Using mixed cultures of primary neurons and astrocytes, infected with HIV, in the presence and absence of SAHA and ART. Cell were microinjected with fluorescent (AÎ^2^1-42-5-tetramethyl rhodamine) and non-fluorescent amyloid-Î^2^1-42 (AÎ^2^1-42) to study cell-to-cell propagation of Î^2^1-42 between TNT connected cells using live-cell imaging and confocal. We demonstrate that HIV reservoirs use TNTs to spread infection and bystander neuronal/glial damage despite suppressive ART. Blocking TNTs prevents viral spread, enhances immune recognition, and reduces the spread of proteins such as APP and Tau. Also, long-term HIV infection impairs the complex between mitochondria and the endoplasmic reticulum (ER), resulting in ER stress and the formation of aggregated toxic proteins such as APP and Tau. Together, these findings identify TNTs contribution to HAND and as novel therapeutic target to improve HIV comorbidities.

Supported by NINDS NS137812

#### 105. Methamphetamine Enhances HIV Infection in Human iPSC-Derived Microglia

Wang, X, MD^1^, Wang, X.W., PhD^1^, RONG, Y.R., MD, PhD^2^, HO, W.H., MD, PhD^1^; ^1^Department of Pathology and Laboratory Medicine, Lewis Katz School of Medicine, Temple University, Philadelphia, PA, 19104 United States. ^2^Department of Pathology and Laboratory Medicine, Temple University Hospital, Philadelphia, PA, 19104 United States.

Methamphetamine (METH) is a potent psychostimulant which is commonly used by people infected with HIV. Clinically, METH use is implicated in HIV infection and neuroinflammation. In this study, we examine whether METH has direct effect on HIV infection of human microglia, the major target and reservoir cells for the virus in the brain. We observed that METH treatment of human iPSC-derived microglia (iMg) significantly enhanced HIV replication, as indicated by increased HIV gag expression, p24 protein levels, and reverse transcriptase activity. Mechanistically, METH suppressed the expression of interferons (IFNs), IFN stimulated gene (Viperin) and the CC chemokine (RANTES). In addition, METH upregulated the expression of the HIV entry coreceptors (CCR5 and CXCR4) in iMg. These findings suggest that METH use is a promoting factor for HIV infection of microglia. Because many individuals infected with HIV use METH, it is important to further investigate the interactions between METH use and HIV in target cells to better understand the mechanisms underlying HIV persistence in the brain and to develop effective strategies for viral eradication.

Supported by This research was funded by the National Institutes of Health (grant numbers DA051893, DA058536, and MH134402).

#### 106. Electronic Cigarette Induces Inflammatory and Oxidative Stress Factors in Human Brain Organoids

Wang, X, MD^1^, Mekala, N, PhD^1^, WANG, X, PhD^1^, SONG, L, PhD^1^, MA, JIE, PhD^1^, RONG, Y, MD, PhD^1^, Persidsky, Y, MD, PhD^1^, HO, WZ, MD, PhD^1^; ^1^Department of Pathology and Laboratory Medicine, Lewis Katz School of Medicine, Temple University, Philadelphia, PA, 19104 United States.

The use of electronic cigarettes (e-cigs) has increased substantially, particularly among young adults and smokers. Although e-cigs are known to induce proinflammatory cytokines and contribute to chronic inflammatory lung diseases, their effects on neuroinflammation remain poorly understood. In this study, we investigated whether e-cig exposure induces inflammatory and oxidative stress responses using a three-dimensional (3D) human brain model-human induced pluripotent stem cell (iPSC)-derived vascularized and microglia-containing cerebral organoids (vMCOs). We assessed the expression of a panel of inflammation- and oxidative stress-related factors in vMCOs following e-cig exposure. Our results show that nicotine-free e-cig exposure had minimal effects, whereas exposure to e-cigs containing 1.8% nicotine selectively and significantly increased the expression of a microglial marker (TMEM119), an oxidative stress enzyme (NOX4), a cytosolic innate immune sensor (NLRP3), and a proinflammatory cytokine (IL-18). No

Supported by This research was funded by the National Institutes of Health (grant numbers DA051893, DA058536, and MH134402).

#### 107. Sleep fragmentation alters microglial function leading to lethal neuroinflammation during sepsis-like inflammation

Wani, A, PhD^1^, Chase, J C, PhD^2^, McKnight, A, PhD^2^, Magne, J, PhD^1^, Mari, L, PhD^1^, Poudel, S, PhD^1^, Janke, L, PhD^3^, Verbist, K C, PhD^1^, Rickman, A D, PhD^4^, Hilyard, A, PhD^4^, Yang, M, PhD^1^, Tummers, B, PhD^5^, Sharma, P, PhD^1^, Saravia, J, PhD^1^, Palacios, G, PhD^1^, Guy, C S, PhD^1^, Shanmugam, R, PhD^2^, Smirnov, I, PhD^6^, Pelletier, S, PhD^7^, Bag, A K, MD, PhD^8^, Rosch, J W, PhD^2^, Kipnis, J, PhD^6^, Heckmann, B L, PhD^4^, Green, D R, PhD^1^; ^1^Department of Immunology, St Jude Children’s Research Hospital, Memphis, TN, 38103 United States. ^2^Department of Host-Microbe Interactions, St. Jude Children’s Research Hospital, Memphis, Memphis, TN, 38103 United States. ^3^Department of Pathology, St. Jude Children’s Research Hospital, Memphis, TN, 38103 United States. ^4^Department of Molecular Medicine, USF Health Byrd Alzheimer’s Center and Neuroscience Institute, Tampa, FL, 33613 United States. ^5^Department of Inflammation Biology, School of Immunology & Microbial Sciences, King’s College London, London, London, SE1 1UL United Kingdom. ^6^Center for Brain Immunology and Glia (BIG), Washington University School of Medicine, St. Louis, St. Louis, MO, 63110 United States. ^7^Department of Medical and Molecular Genetic, Indiana University School of Medicine, Indianapolis, Indianapolis, IN, 46202 United States. ^8^Department of Radiology, St. Jude Children’s Research Hospital, Memphis, Memphis, TN, 38103 United States.

It is well established that infection can promote slow wave sleep, but the functional importance of this phenomenon remains unclear. In this study, we utilized sleep fragmentation, i.e., intermittent disruption of sleep throughout the day, to explore the relationships between sleep and inflammatory disease. We found that sub-lethal injection of a bacterial lipopolysaccharide (LPS), prior to sleep fragmentation (SF) invariably resulted in dramatically increased mortality compared to LPS only or SF only controls, highlighting the importance of sleep following an inflammatory challenge. Further investigation revealed hyperactivation of microglia in the hypothalamus and other brain areas in mice subjected to LPS plus sleep fragmentation. As Toll-like receptor-4 (TLR4) is crucial for responses to LPS, we examined animals with microglia-specific deletion of TLR4 and found that it completely protected mice from the combination treatment. To further explore the role of sleep, we generated Dec2 (BHLHE41) mutant mice that display decreased sleep and found that these animals are protected from low dose LPS plus SF. We are currently extending our results to bacterial infection.

#### 108. Immune Metabolic Correlates of Self-Harm Thoughts and Somatic Depressive Symptoms in South Africans With HIV 1 Subtype C

Williams, ME, PhD^1^, Schutte, L, PhD^2^, Asia, LK, MS^1^, Wissing, MP, PhD^2^, Jansen Van Vuren, E, PhD^3^; ^1^Biomedical and Molecular Metabolism Research (BioMMet), North-West University, Potchefstroom, North West, 2531 South Africa. ^2^Africa Unit for Transdisciplinary Health Research (AUTHeR), North-West University, Potchesftroom, North West, 2531 South Africa. ^3^Hypertension in Africa Research Team (HART), North-West University, Potchesftroom, North West, 2531 South Africa.

Depressive symptoms in people living with HIV may cluster into somatic and cognitive affective domains that relate differently to neuroimmune and metabolic activity. We examined whether baseline immune and tryptophan kynurenine pathway markers were associated with PHQ 9 self-harm thoughts and depressive symptom subscales in a South African HIV-1 subtype C cohort. Treatment-naive adults with HIV were assessed at baseline in 2010 (n = 69) and followed in 2015 (n = 40). Baseline measures included kynurenine pathway metabolites and inflammatory markers, while depressive symptoms were assessed using the PHQ-9. Lower baseline NGAL levels were observed in participants who later reported self-harm thoughts and were associated with greater odds of self-harm thoughts at follow-up (*p* = 0.027 after Hochberg correction). At baseline, quinolinic acid (p < 0.001), kynurenine (*p* = 0.003), hsCRP (*p* = 0.007), and suPAR (*p* = 0.001) were positively associated with somatic symptoms, while the KA:QUIN ratio showed an inverse association (*p* = 0.004). No markers were associated with cognitive affective symptoms or follow-up subscales after correction. These findings suggest that immune metabolic processes may relate more strongly to somatic depressive symptoms, while lower NGAL may reflect vulnerability to later self-harm thoughts. Findings are exploratory and warrant confirmation in larger longitudinal studies.

Supported by National Research foundation, South Africa

#### 109. Preclinical and Translational Novel PET Imaging Methodologies for Quantification of In Vivo Neuroimmune Function

Wong, Dean, MD, PhD^1^, Tu, Z, PhD^1^, Horti , A, PhD^3^, Nai, YH, PhD^2^, Lee, J, MD, PhD^2^, Chand, G, PhD^2^, Kuwabara, H, MD, PhD^4^, Dannals, RF, PhD^3^; ^1^Kahlert Institute of Addiction Medicine , Depts of Neurosurgery & Radiology, University of Maryland, Baltimore, MD, 21201 United States. ^2^Division of Radiation Sciences, Washington University in St. Louis, St Louis, MO, 63110-1030 United States. ^3^Department of Radiology, Johns Hopkins University, Baltimore, MD, 21201 United States. ^4^Department of Psychiatry, Johns Hopkins University, Baltimore, MD, 21201 United States.

PET imaging of neuroreceptor and neurochemical systems have been available since 1983 in human brain and have dramatically led the way for the study of neuropsychiatric mechanisms in healthy and diseased states. All such PET projects arise from a long and rigorous use of novel pharmacological identification, radiolabeling with F-18 or C-11 primarily and validation in pre-clinical experiments. In this presentation a review of over 45 years of developing such PET tracers and translation will be provided. Specific attention will be given to the neuroimmune focus. Some examples will include the authors’ experience with nAchR alpha 7 tracers (F-18 ASEM) in traumatic brain injury and schizophrenia, a possible inflammatory etiology, While TSPO has been a popular target, we will show how new approaches such as imaging the S1P1 site has already shown remarkable biomodal distribution of patients with schizophrenia in postmortem tissue , corresponding to molecular markers and the in vivo published biomodal populations based on structural MRI and phenotypes with machine learning tools . We will show results in HIV AIDs dementia and amyloid PET, We will also provide preliminary data on rodent and non-human primate imaging of C-11 S1P1 in the validation towards its current use in healthy humans and Multiple Sclerosis , some of which is published and some publicly disclosed , In sum this will provide an overview of the new frontiers of neuroimmune imaging

Supported by NIMH, NIA, NINDS

#### 110. Sex-Specific Behavioral Effects of Acute CBD:THC Exposure in an EcoHIV Mouse Model

Yadav-Samudrala, BJ, PhD^1^, Palacios, M^2^, Miller, E^3^, McRae, MP, PhD^3^, Fitting, S, PhD^1^; ^1^Department of Psychology and Neuroscience, University of North Carolina Chapel Hill, Chapel Hill, NC, 27599 United States. ^2^Department of Biology and Chemistry, Liberty University, Lynchburg, VA, 24515 United States. ^3^Department of Pharmacotherapy and Outcome Science, Virginia Commonwealth University, Richmond, VA, 23284 United States.

Despite effective viral suppression with combined antiretroviral therapy, nearly 50% of people living with HIV experience HIV-associated neurocognitive disorders (HAND). The endocannabinoid system has been shown to modulate HIV neuropathogenesis by attenuating neuroinflammation and limiting viral replication. This study examined the effects of acute exposure to a low-potency CBD:THC ratio on behavior and antinociception in an EcoHIV mouse model. Adult C57BL/6J mice (N = 36; 17 females) were inoculated intraperitoneally with saline or EcoHIV. At 21 days post-inoculation, baseline behavioral testing included open field, Y-maze, elevated plus maze (EPM), tail withdrawal, and hot plate assays. Mice then received an acute CBD:THC injection (15:1; 75 mg/kg CBD, 5 mg/kg THC), followed by repeat testing. Baseline open-field testing showed significant effects of sex, genotype, and sex x genotype interaction, indicating sex- and genotype-dependent anxiety-like behavior. In the EPM, females spent more time in open arms compared to males, indicating reduced anxiety-like behavior. No effects were noted for males. EcoHIV inoculation impaired Y-maze performance, indicating memory deficits. After CBD:THC treatment, effects were observed only in females, including reduced novel arm entries and open-arm exploration, with increased tail withdrawal and hot plate latencies. Acute low-potency CBD:THC treatment produced sex-specific changes in behavior, memory, and nociception, predominantly affecting female mice.

Supported by NIDA R01 DA055523, R01 DA064394

#### 111. Neuro-Microglial Immune Circuits in HIV-Associated Neurocognitive Disorder under Substance Abuse

Yoh, S, PhD^1^; ^1^Dept of Immunology and microbiology, Scripps Research, La Jolla, CA, 92037 United States.

Microglia serve as the primary immune sentinels of the brain and are essential for CNS homeostasis and pathogen clearance. Despite effective combination antiretroviral therapy (cART), many people with HIV (PWH) continue to experience persistent neuroinflammation in the CNS, even in the absence of productive viral replication. This persistent immune activation contributes to HIV-associated neurocognitive disorders (HAND). Notably, individuals with substance use disorder and poor immune recovery (immunological non-responders) are particularly vulnerable to HAND pathogenesis. Currently, the field lacks mechanistic understanding of how psychostimulant exposure, defective proviral expression, and innate immune activation in the CNS interact to drive HAND pathogenesis. We hypothesize that persistent expression of immunogenic viral elements from microglial viral reservoirs initiates chronic innate immune activation, while psychostimulant-induced neuronal stress amplifies this state through DAMP release, creating a synergistic degenerative feedback loop that accelerates neuronal injury. Our goal is to define the key innate immune pathways through which HIV-1 and psychostimulant use synergistically promote chronic neuroinflammation.

Supported by NIH/NIAID R01 AI 177265

#### 112. Biochemical Alterations in the Thalamus of People Living With HIV

Zahr, N.M., PhD^1^, Pfefferbaum, A., MD^2^; ^1^Department of Psychiatry and Behavioral Sciences, Stanford University, Stanford, CA, 94305 United States. ^2^Neuroscience Department, SRI International, Menlo Park, CA, 94025 United States.

We previously demonstrated in HIV that higher than control striatal choline (Cho) levels were related to HCV infection. To determine whether HCV specifically or liver damage generally affects brain biochemicals, data were collected in thalamus and parietal white matter (PWM) of people with HIV (n=33, 5 women), HIV+AUD (n=21, 6 women), and healthy controls (n=80, 42 women). The serum-based FIB4 (age * aspartate aminotransferase / platelet count * alanine aminotransferase) indicated liver fibrosis (i.e., scores>1.5). Metabolites with Cramer Roa bounds <15% [i.e., Cho, mI, N-acetyl-aspartate+ N-acetyl-aspartyl-glutamate (NAA), creatine (Cr), glutamate+glutamine (Glx), macro-molecules+-lipids at 0.9ppm (MM09+Lip09)] were considered. Coil loading effects required use of metabolite ratios relative to Cr. Models including age, HCV, and FIB4 explained <10% of PWM metabolite variance. In thalamus, statistically non-significant models explained 17% of NAA, 13% of Cho, and 11% of Glx variance; HCV relative to FIB explained more of the variance. In the thalamus, ANOVAs demonstrated lower NAA (p=.0028) and higher Cho (p=.0049) in the 2 HIV groups relative to controls. Across all participants, lower NAA was related to greater lifetime alcohol consumed (r=-0.22, p=.01); higher Cho with greater past year drinking (r=.18, p=.05). These results suggest that HCV, not liver fibrosis generally, affects thalamic metabolites in HIV. Cho findings related to drinking recency are provocative because Cho increases are reliably observed in rodent brains in response to ethanol exposure.

Supported by AA017347.

#### 113. Taming Alu in the Primate Brain: Ku Suppresses RNA-Mediated Innate Immunity and Reshapes the Transcriptome

Zha, SZ, MD, PhD^1^, Zhang, C, PhD^1^, Zhu, T, PhD^1^, Yu, T, BS^1^, Yoon, J, PhD^1^, Lee, B, BS^1^; ^1^Institute for Cancer Genteics, Columbia University, New York, NY, 10032-3802 United States.

The massive Alu expansion in higher primates enables novel neuronal subtypes and function, but also generates abundant dsRNA, which can activate innate immune sensors and disrupt RNA processing. Ku70 and Ku80 form the Ku heterodimer, best known for initiating non-homologous end joining (NHEJ) at DNA double-strand breaks. Although Ku can bind dsRNA, its RNA-dependent physiological role remained unclear. Ku is dispensable for murine development but essential in human cells, and its expression increases sharply ∼ 100-fold during primate evolution in parallel with Alu expansion. Here, we show that Ku acts as a primate-specific RNA regulator that suppresses RNA-mediated innate immunity and shapes the human transcriptome. Ku depletion in human cells, unlike loss of core NHEJ factors, triggers interferon and NF-κB signaling through the dsRNA sensors MDA5 and RIG-I and the adapter MAVS, followed by activation of PKR and the OAS/RNase L pathway, leading to translational arrest and growth inhibition. Ku directly binds dsRNA stem-loops enriched in primate-specific antisense Alu elements, limiting aberrant immune activation. In parallel, Ku suppresses Aluassociated alternative splicing independently of IFN and NHEJ, affecting ∼8-10% of splicing events. Strikingly, Ku expression is lower in brain, correlating with more permissive Alu-derived splice variants critical for neuronal function. Together, these findings identify Ku as a key evolutionary adaptation that tames Alu expansion by coordinating innate immune suppression and transcriptome remodeling critical for human brain function.

Supported by NIH and National Cancer Institutes

#### 114. HIV-1 Tat Protein Exposure Alters the Morphological Characteristics and Gene Expression in the Primary Mouse Cortex Endothelial Cells and Human Brain Microvascular Endothelial Cells

Zhu, J, MD, PhD^1^, Quan, LL, PhD^2^, Manabe, I, MD^3^, Muramatsu, R, PhD^2^; ^1^Augusta University, Medical College of Georgia, Augusta, GA, 30912 United States. ^2^National Center of Neurology and Psychiatry, Department of Molecular Pharmacology, National Institute of Neuroscience, Japan, Tokyo, 187-8502 Japan. ^3^Chiba University, Graduate School of Medicine, Japan, Tokyo, 260-8670 Japan.

Damage of blood-brain barrier (BBB) may serve as an early biomarker of cognitive dysfunction in people living with HIV. This is due to the ability of HIV-1, along with infected monocytes and macrophages, to traverse the BBB via either paracellular or transcellular way. HIV-1 viral proteins have been shown to disrupt tight junctions within the BBB, thereby directly compromising its structural and functional integrity. This study determined the effects of the HIV-1 Tat protein on the morphological profiles and gene expression of mouse prefrontal cortex endothelial cells (ECs) and human brain microvascular endothelial cells (HBMVEC) 48 hours after in vitro exposure to 12.5 nM recombinant Tat1-86. After Tat treatment, cells were immunostained with CD31, anti-Tat, DAPI or phalloidin, and harvested for RNA sequencing to access changes in gene expression. Staining results showed a reduction in CD31 expression accompanied by an increase in phalloidin staining intensity in both mouse ECs and HBMVECs after Tat exposure. The phalloidin staining revealed disruption of actin cytoskeleton structure in both mouse ECs and HBMVECs after Tat exposure. RNA sequencing analysis of mouse ECs and HBMVECs exposed to Tat displayed strikingly comparable transcriptomic signatures, as confirmed by gene set enrichment analysis. Both mouse ECs and HBMVECs showed significant upregulation of hallmark inflammatory response pathways following Tat exposure. These findings provide mechanistic insight into HIV-1 Tat drives endothelial injury, leading to both morphological and transcriptional alterations.

Supported by NIH grants DA035714, DA047924 and DA057866

#### 115. Cryopreservation Preserves Cellular Integrity and Innate Immunity in Human iPSC-Derived Brain Organoids

Xiao-Long Wang, Li Song, Xu Wang, Yuan Rong, and Wen-Zhe Ho, Department of Pathology and Laboratory Medicine, Temple University Lewis Katz School of Medicine, Philadelphia, PA 19140, United States.

Human iPSC-derived brain organoids are increasingly used for neurobiological research; however, their widespread application is limited by long-term cultivation requirements, contamination risk, and high cost. Therefore, reliable storage of brain organoids is essential for biobanking to ensure reproducible studies in disease modeling. Here, we investigated whether cryopreservation preserves cellular composition and innate immune function in human iPSC-derived brain organoids containing microglia and BBB endothelial cells. We demonstrate that cryopreservation preserves the cellular composition and molecular identity of these organoids, as evidenced by comparable mRNA and protein expression levels of major brain cell markers (GFAP, IBA-1, TUJ-1, MAP2, TMEM119, and OLIG2) between cryopreserved and control organoids. Importantly, cryopreserved and subsequently revived organoids retained immunological functionality. Both freshly cultured and cryopreserved-revived organoids mounted robust innate immune responses following stimulation with the TLR3 ligand Poly(I:C), as demonstrated by significant transcriptional upregulation of IFN-β, Viperin, and ISG56. Collectively, these findings indicate that cryopreservation preserves structural and immunological integrity of human iPSC-derived brain organoids, supporting its use as a reliable biobanking strategy for scalable and reproducible neurobiological research.

